# Stoichiometrically Engineered Hydrated Ionic Liquids Enabling Reinforcement of Enzyme Cascade with Improved Thermodynamic Stability

**DOI:** 10.1021/acssuschemeng.5c13384

**Published:** 2026-03-16

**Authors:** Sagar Biswas, Dheeraj Kumar Sarkar, Aaftaab Sethi, Pranav Bharadwaj, Rakesh Sinha, Pankaj Bharmoria, Gregory Franklin, Dibyendu Mondal

**Affiliations:** † Institute of Plant Genetics, Polish Academy of Sciences, Strzeszyńska 34, Poznań 60-479, Poland; ‡ Tata Institute of Fundamental Research, Hyderabad, Telangana 500046, India; § Laboratory of Biomolecular Interactions and Transport, Department of Gene Expression, Institute of Molecular Biology and Biotechnology, Faculty of Biology, Adam Mickiewicz University, Uniwersytetu Poznanskiego 6, Poznan 61-614, Poland; ∥ Institute of Materials Science of Barcelona, ICMAB-CSIC, Barcelona 08193, Spain; ⊥ Centre for Nano and Material Sciences, 250161 Jain (Deemed-to-be University), Jain Global Campus, Kanakapura, Bangalore, Karnataka 562112, India; # Department of Chemical Engineering, Universitat Politècnica de Catalunya, EEBE, Eduard Maristany 1014, Barcelona 08019, Spain

**Keywords:** pH-switchable ionic liquids, enzymatic cascades, microenvironment modulation, protein engineering, thermo-stress tolerance, sustainable biocatalysis

## Abstract

While biocatalysis in ionic liquids (ILs) using a single enzyme is well known, the successful performance of enzyme cascade reactions (ECRs) using multiple enzymes in ILs is limited by the incompatible stabilization of more than one enzyme in a single IL. Here, we introduce an innovative approach where stoichiometric precision of ILs creates pH-switchable media that dynamically modulate multienzyme microenvironments and maintain the functional integrity of ECR without requiring any proximity-engineered scaffolds. Cholinium-based ILs, with phosphate and carboxylate anions, were synthesized with varying molar ratios of cholinium to realize pH-switchable aqueous platforms for ECR. Using glucose oxidase (GOx)–horseradish peroxidase as (HRP) an enzymatic cascade, we demonstrate that under optimized conditions aqueous solutions of ILs significantly enhance both the individual enzyme (GOx and HRP) activities and ECR (GOx–HRP) efficiencies compared to the control, phosphate-buffered saline (PBS) (pH 7.4). Molecular docking, molecular dynamics simulations, UV–vis, and circular dichroism spectroscopy studies reveal that ILs are involved in soft interactions with enzymes, stabilizing catalytically favorable conformations, and protecting enzymes against thermal-stress. Remarkably, a 25-fold increase in the ECR efficiency was achieved in 10 wt % of [Ch]_2_[PAA] through [Ch]_2_[PAA] assisted improved substrate channeling and reduced transition-state energy barriers. Moreover, an ∼16% increase in the half-life temperature (*T*_50_) of GOx–HRP cascade in the presence of 10 wt % [Ch]_2_[PAA] with an enhanced melting temperature (*T*_m_) of the enzymes suggested improved thermal stability relative to PBS. The results of improved enzyme stability in hydrated ILs were further investigated by the thermodynamic stability curves (Δ*G* vs *T*). Overall, this work provides a basis for multienzyme biocatalysis in aqueous solution of ILs with an accelerated ECR rate and improved thermodynamic stability, envisaging sustainable biocatalysis and metabolic engineering.

## Graphical abstract

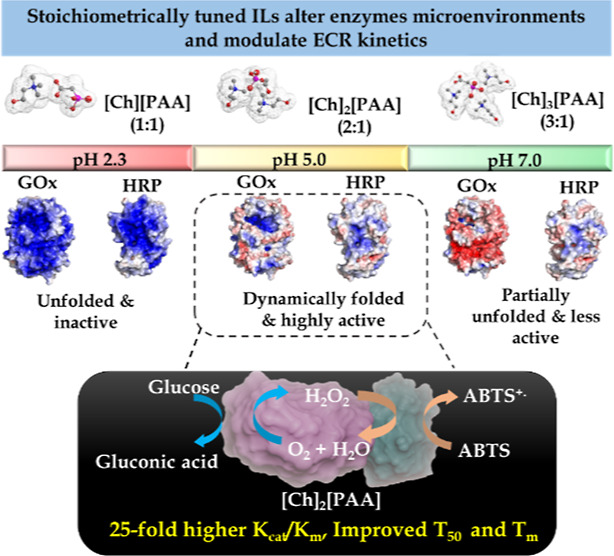

## Introduction

Enzyme cascade reactions (ECRs) play a crucial role in cell biology by facilitating complex molecular synthesis through thermodynamically optimized pathways.^1,2^ The study of these reactions in vitro enables a deep understanding of the dynamic regulation of biological communication networks, the discovery of new metabolic pathways, and gene regulation.^3–5^ For example, ECRs produced in vitro have helped to (1) identify the novel metabolic pathways of carbohydrate metabolism,^6^ (2) understand the mechanisms underlying the biosynthesis of secondary metabolites,^7^ and (3) uncover ECR kinetics in the total synthesis of natural products, drugs, and platform chemicals.^8,9^ However, replication of in vitro ECRs with improved kinetics faces challenges such as irreversible damage to the enzyme microenvironment, suboptimal substrate channeling, the proximity effect, and various biotic or abiotic stress factors.^10–13^ Substrate channeling can be improved by creating a favorable microenvironment—through membranes,^11^ molecular crowding agents,^14^ or DNA scaffolds and covalent organic frameworks^15–21^ —while reducing diffusion losses and improving the kinetics of ECRs. Previous research has emphasized the crucial role of crowding agents and biomolecular condensates in regulating protein dynamics and improving enzymatic kinetics.^22,23^ The physicochemical properties of the ECR microenvironment, including pH, temperature gradient, viscosity, and ionic strength, have a significant impact on the thermodynamic stability, conformational dynamics, and activity of enzymes.^24,25^ Therefore, to achieve a high biocatalytic yield, the microenvironment conditions must be tailored for each enzyme involved in an ECR.

The stability of proteins in cellular structures is strongly influenced by soft interactions,^26^ which play a crucial role in the folding of proteins into highly active conformations. Recent studies suggest that ionic liquids (ILs) can modulate the activity of enzymes through soft interactions such as H-bonds and ionic bonds.^27–29^ ILs offer applications in many scientific disciplines, including cell biology^30^ and biocatalysis, due to their tailored physiochemical properties.^31^ With respect to proteins, ILs are often used to purify, stabilize, and activate proteins by tailoring their microenvironment.^32^ This is due to the different polarities of ILs, which possess interaction sites that facilitate ionic interactions, hydrogen bonding, and hydrophobic associations with proteins in solution. Such interactions can alter the secondary and tertiary structural conformations of proteins, ultimately affecting the enzymatic activity, both negatively and positively due to changes in the active site. The pH can strongly influence the catalytic activity of an enzyme because key amino acid residues involved in catalysis have different ionization states, so different enzymes function best in a particular pH range.^25^ Therefore, pH-switchable ILs offer immense opportunities to alter the microenvironment of ECRs to maximize the biocatalytic efficiency.

Although ILs have been reported to stabilize various proteins (enzymes) with increased activity, investigation on protein–protein interactions and ECRs in protein–IL systems is scant.^33^ By using pH-tuned ILs, we present a novel and straightforward method to increase the rates of the glucose oxidase–horseradish peroxidase (GOx–HRP) cascade reaction without the need for complicated nanostructures, such as nearby systems like nanocages.^15–17^ Our results suggest that the strategic design of pH-switchable ILs significantly increases ECR reaction rates and enzyme thermodynamic stability by modulating the enzyme microenvironment compared to conventional phosphate buffer saline (PBS). These results elucidate the key interactions between the multienzyme cascade and the IL systems and reveal an IL-based robust platform for improved tandem biocatalysis and sustainable chemical synthesis.

## Experimental Section

Details of materials, IL synthesis, pH measurements, activity assays of horseradish peroxidase as (HRP) and GOx, second derivative UV analysis, thermodynamic stability of studies using circular dichroism (CD) spectroscopy, SDS-PAGE analysis, and computational methods are provided in the Supporting Information.

### GOx–HRP Cascade Assay at a Varying Ratio of GOx to HRP in Different ILs

The true essence of enzymatic reaction is evaluated as the enzyme-coupled system as most enzymes in biological systems function in a cascade reaction network. Hence, we build a GOx–HRP coupled system to evaluate the cascade effectiveness in the designed ILs as enzyme packaging media. Mostly enzymes in the cascade-coupled system function in an equimolar ratio. Therefore, the final assay solution of the GOx–HRP cascade reaction was constructed using 1 nM GOx and 1 nM HRP. 2 mM ABTS and 100 mM d-glucose were also used in the assay solution. To prepare the GOx–HRP cascade assay 10 μL of 100 nM GOx and 10 μL of 100 nM HRP were individually incubated for 5 min and mixed well prior to the addition of 50 μL of 40 mM ABTS solution. Finally, 50 μL of a 2 mM glucose solution was added to the incubated mixture prior to the measurement of absorbance. The final assay solution volume was maintained at 1 mL for each experiment. In all the cases, the IL concentration was 10 to 50 wt % and PBS (pH 7.4) was taken as a reference solution to obtain the relative activity. Relative activity (RA) of GOx–HRP cascade was calculated from the following formula.^16^

$$\%\;RA=\frac{\left(slope\;of\;GOx-HRP\;ECR\;in\;ILs\times 100\right)}{\left(slope\;of\;GOx-HRP\;ECR\;in\;buffer\right)}$$

The activity of HRP, GOx, and GOx–HRP cascades in 100 mM PBS (pH 7.4) at 37 °C was taken as 100% and the activity profiles of all the systems were calculated. All of the enzymatic assay experiments were replicated at least 3 times. ECR was also replicated with different batches of ILs.

### Activity Assay of HRP and GOx–HRP Cascades under Thermal Stress

The effect of temperature on the activity of HRP and GOx–HRP (1:1 mol ratio) cascades was also performed in the presence of different ILs ([Ch]_2_[Dhp], [Ch]_2_[Mal], and [Ch]_2_[PAA]) by incubating the HRP, the GOx sample in aqueous solution of 10 wt % ILs for 15 min at the temperature range of 37 to 80 °C followed by measuring the enzyme activity. The remaining activity was calculated at different temperatures by considering the activity of each solvent system at 37 °C as 100%. The half activity temperature (*T*_50_) was also evaluated upon obtaining the activity at the temperature range of 37–80 °C, wherein the activity was deduced to 50% of the initial activity. All the enzymatic assay experiments were replicated at least 3 times.

### Kinetics Assessment of HRP, GOx, and GOx–HRP Cascades in ILs

Kinetic parameters were individually evaluated for the HRP, GOx, and GOX–HRP (1:1 mol ratio) cascades in [Ch]_2_[Dhp], [Ch]_2_[Mal], and [Ch]_2_[PAA] at optimized concentrations (10 wt %) and PBS (100 mM, pH 7.4). The enzyme assay was prepared following the same protocol as described in enzyme assay preparation, and the substrate concentration was varied. The change in UV–vis absorbance at 420 nm was monitored for all of the enzymatic assays. For the HRP kinetic assay, the substrate H_2_O_2_ was added (10 μL) in separate reaction mixtures in the range of 100 nM to 1000 μM, and the concentration was increased until the reaction rate saturates. In each case, the reaction was monitored for up to 3 min. To obtain kinetic parameters of GOx, a similar strategy was employed as HRP, and d-glucose was chosen as a substrate (added volume 50 μL) with a concentration range of 0.2 to 200 mM. While considering the GOx–HRP cascade, ABTS (50 μL) was chosen as a substrate with a range of 0.005–5 mM. All the reaction velocities (*V*) were then calculated to evaluate kinetic models. We have validated the obtained kinetic data with three well-known kinetic models, such as Michaelis–Menten (M–M), Lineweaver–Burk (L–B), and Eadie–Hofstee (E–H). To obtain precise kinetic parameters, such as *V*_max_, *K*_m_, and *K*_cat_, the cascade enzymatic reaction is shown as eqs 1 and 2 when enzymes (E1 and E2) bind with a substrate (S and P), an enzyme–substrate complex (ES) is formed, which can further produce product P.

1 E1+S⇌E1S→E1+P1

2 E2+P1⇌E2P1→E2+P2

Based on the above equations, M–M (*V* vs [S]) (eq 3), L–B (1/*V* vs 1/[S]) (eq 4), and E–H (*V* vs *V*/[S]) (eq 5) plots were constructed, respectively, to corroborate the enzymatic kinetics.

3 $$V=\frac{V_{\max}\cdot \left[S\right]}{K_{m}+\left[S\right]}$$

4 $$\frac{1}{V}=\frac{K_{m}}{V_{\max}}\frac{1}{\left[S\right]}+\frac{1}{V_{\max}}$$

5 $$V={-K}_{m}\frac{V}{\left[S\right]}+V_{\max}$$

## Results and Discussion

### pH-Switchable ILs and Enzymes Cascade System

Choline ([Ch]^+^) cation-based ILs with phosphate and carboxylate anions (Figure 1A) were synthesized using a simple acid–base reaction (Supporting Information, Section S2). The ^1^H NMR data show no detectable impurities within the sensitivity of the measurements, supporting the high purity of the prepared ILs (Figures S1–S8). Choline was chosen as a cation because of its known bio-origin and it is the part of phospholipid biomembrane.^34^ Choline-based ILs with dihydrogen phosphate anions ([Ch]­[Dhp]) is a well-studied for protein stabilization, where [Dhp]^−^ plays a key role in stabilizing the protein via dual hydrogen bonding with the protein surface.^32^ Similarly, carboxylate anions (RCOO^–^) were chosen because of their demonstrated role in boosting the activity of enzyme cytochrome *c*.^34^ Additionally, the phosphate and carboxylate anions can also assist in modulating the pH of aqueous solution.^34^ Three series of ILs were synthesized using [Ch]^+^ combined with phosphate ([Ch]­[Dhp], [Ch]_2_[Dhp] and [Ch]_3_[Dhp]); malonate ([Ch]­[Mal] and [Ch]_2_[Mal]); and phosphonoacetate ([Ch]­[PAA], [Ch]_2_[PAA] and [Ch]_3_[PAA]) anions. The choline was used in different stoichiometries with respect to various anions (1:1, 2:1, and 3:1) to switch the pH state of the aqueous media upon IL dissolution. The variation of pH (pH 2.3 to 8.1) was regulated by optimizing the stoichiometry of the ILs with concentration 10 to 50 wt % in the deionized water (Figure 1C and Table S1). All ILs with 1:1 molar composition [Ch]­[Dhp], [Ch]­[Mal], and [Ch]­[PAA], exhibited low pH values (pH = 2.3–3.08), whereas [Ch]_3_[Dhp] and [Ch]_3_[PAA] having 3:1 molar composition showed higher pH values (7.45 to 8.14). Moreover, ILs [Ch]_2_[Dhp], [Ch]_2_[Mal], and [Ch]_2_[PAA] with a 2:1 molar ratio showed a moderate pH range of 4.82 to 7.01. The score plots of the first two principal components (PCs), which together account for 99.9% of the total variability (Figure 1D), expose clear differences among the ILs across different molar ratios and pH values. Three distinct groups can be identified: group-1: [Ch]­[Dhp], [Ch]­[Mal], and [Ch]­[PAA]; group-2: [Ch]_2_[PAA] and [Ch]_2_[Mal]; and group-3: [Ch]_3_[Dhp] and [Ch]_3_[PAA], which show marginal differences compared to [Ch]_2_[Dhp]. The pH of these ILs impacts side chains of amino acids such as Asp, Glu, Lys, and Arg that substantially alter the residual charge of enzymes and impact their solubility and folding propensity as well as stability.

**Figure 1:**
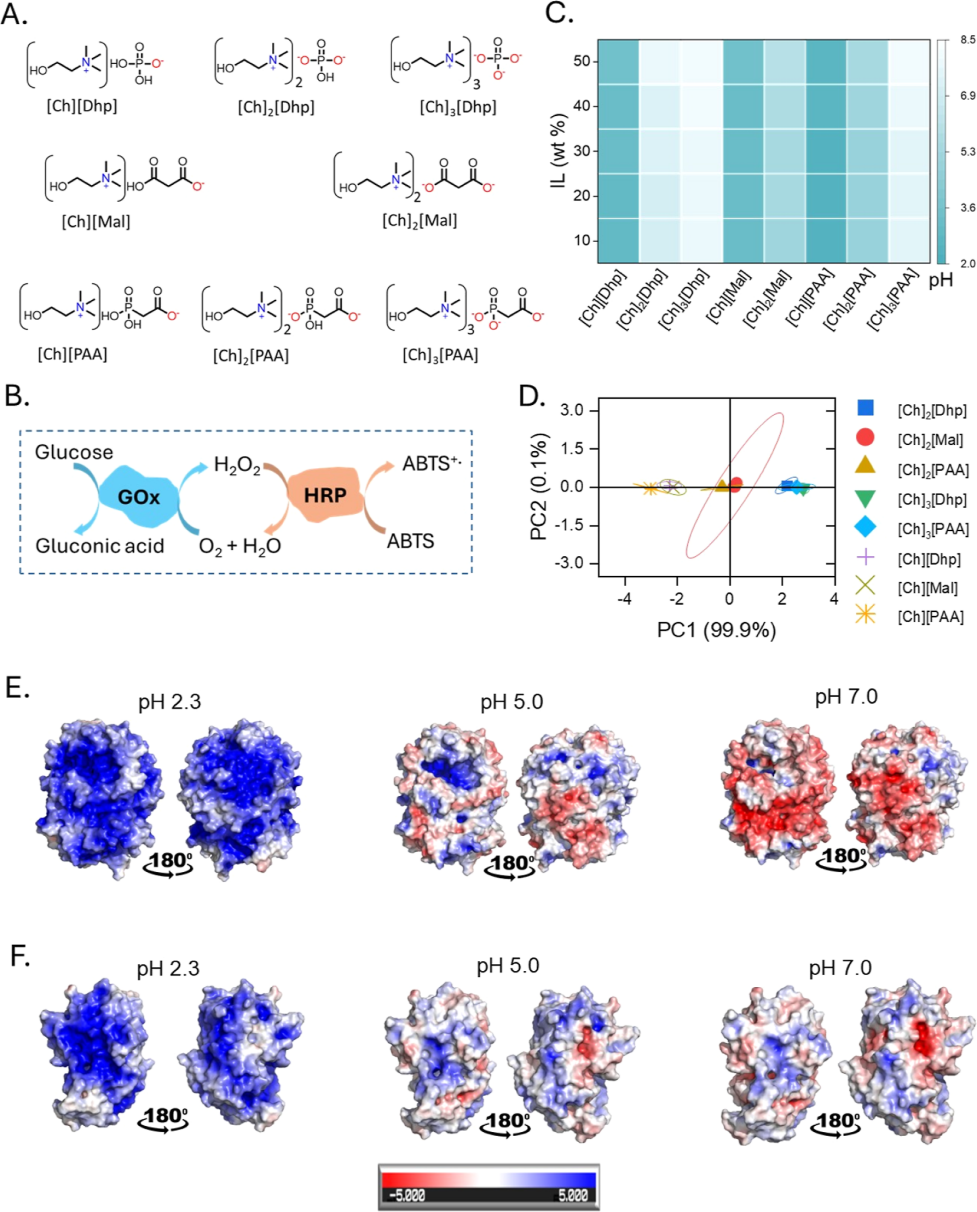
(A) Chemical structures of the different ILs. (B) Schematic representation of the GOx–HRP cascade reaction using glucose and ABTS as substrates for GOx and HRP, respectively. (C) Heat map showing the pH mapping of all ILs at different IL concentrations. (D) Principal Component Analysis (PCA) to analyze the pH values of the different ILs at concentrations from 10 to 50 wt %. In the dot plot, the same data sets are close to each other, while the different data sets are further apart. (E,F) Plots of the electrostatic potential surface of GOx and HRP, showing the electrostatic potential surface at pH 2.3, 5.0, and 7.0, respectively. The following applies here: +5 *KT*/*E* = +129.3 MV, −5 *KT*/*E* = −129.3 MV.

The HRP–GOx was chosen as a model enzyme cascade reaction system because of their variable surface charge density at different pH revealed from electrostatic surface potential (ESP) modeling (Figure 1E,F). Notably, residues such as His, Asp, and Glu were affected near the catalytic region of HRP and GOx. The effect is more prominent in GOx than in HRP, as the ESP is almost neutralized in the case of HRP and more negative in GOx (Figure 1E,F). The total charges of the GOx system are +54.0, −2.9, and −24.0 units at pH 2.3, 5.0, and 7.0, respectively. In contrast, for HRP, the total charge varies from +28.5, +5.5, and +0.5 units at pH 2.3, 5.0, and 7.0, respectively. As observed from the EPS, the catalytic residue His516 undergoes a charge shift of 4 units when the pH changes from 2.3 to 7.0 in GOx. Similarly, for HRP, the charge difference for the catalytic residue His42 is also 4 units. Thus, pH of ILs has a significant impact on EPS, the enzymes that would significantly impact the stability and activity of GOx and HRP. In general, GOx and HPR show an optimum surface charge distribution in the pH ranges of 5–7 and result in maximal catalytic turnover, which also corresponds to the optimal surface charge distribution of the enzymes.^35,36^ Thus, we investigated the impact of different pH-tuned ILs and their composition on the biological activity of the HRP, GOx, and GOx–HRP cascades as discussed below.

### Investigations on Synergistic Interplay between IL Structure, pH, and Enzyme Ratios in the Cascade Activity

To elucidate the impact of ILs on the activity of an individual enzyme and in the cascade system, systematic investigations of the effect of pH-switchable ILs (conc. = 10–50 wt %) on the HRP, GOx, and GOx–HRP cascade activity were carried out. The GOx–HRP cascade activity was monitored using a standard protocol involving ABTS oxidation observed at the absorption maximum, λ_max_ = 420 nm (Supporting Information, Section S4).^37,38^ The relative peroxidase activity of the enzyme was monitored over time in reference to PBS buffer (pH 7.4) as the control (Figures S9–S11). First, the activity of enzymes was monitored in 10 wt % of choline cations and Dhp anion-based ILs, which show different pH values in water upon changing the number of choline cations and IL concentration. HRP showed negligible activity in [Ch]­[Dhp] (pH 2.8 to 3.5; Figure 2A). Whereas upon increasing the choline cation, [Ch]_2_[Dhp] (10 wt %, pH 7.01), the HRP activity was found to be ∼60% compared to PBS, which declined further upon increasing the concentration above 10 wt % (Figure 2A). A similar activity trend was also observed for [Ch]_3_[Dhp] (pH 7.7 to 8.1) upon increasing the IL concentration (10 to 50 wt %) (Figure 2A). A comparable activity trend with single choline cations was also observed for the malonate anion-based IL, [Ch]­[Mal]. The [Ch]­[Mal], with a more acidic pH (3 to 3.5), showed negligible HRP activity (Figure 2B). However, further increasing the number of choline cations to two; [Ch]_2_[Mal] (10 wt %; pH 5.2), resulted in a 4.4-fold rise in relative activity HRP, compared to PBS (Figure 2B). Further replacement of malonate anions with PAA anions, [Ch]_2_[PAA] (10 wt %; 4.82 ± 0.015) resulted in a significant jump in HRP activity (6.4-fold more than PBS) (Figure 2C). In contrast, a negligible HRP activity was observed in [Ch]­[PAA], (10 wt %; pH ∼ 2.3).

**Figure 2:**
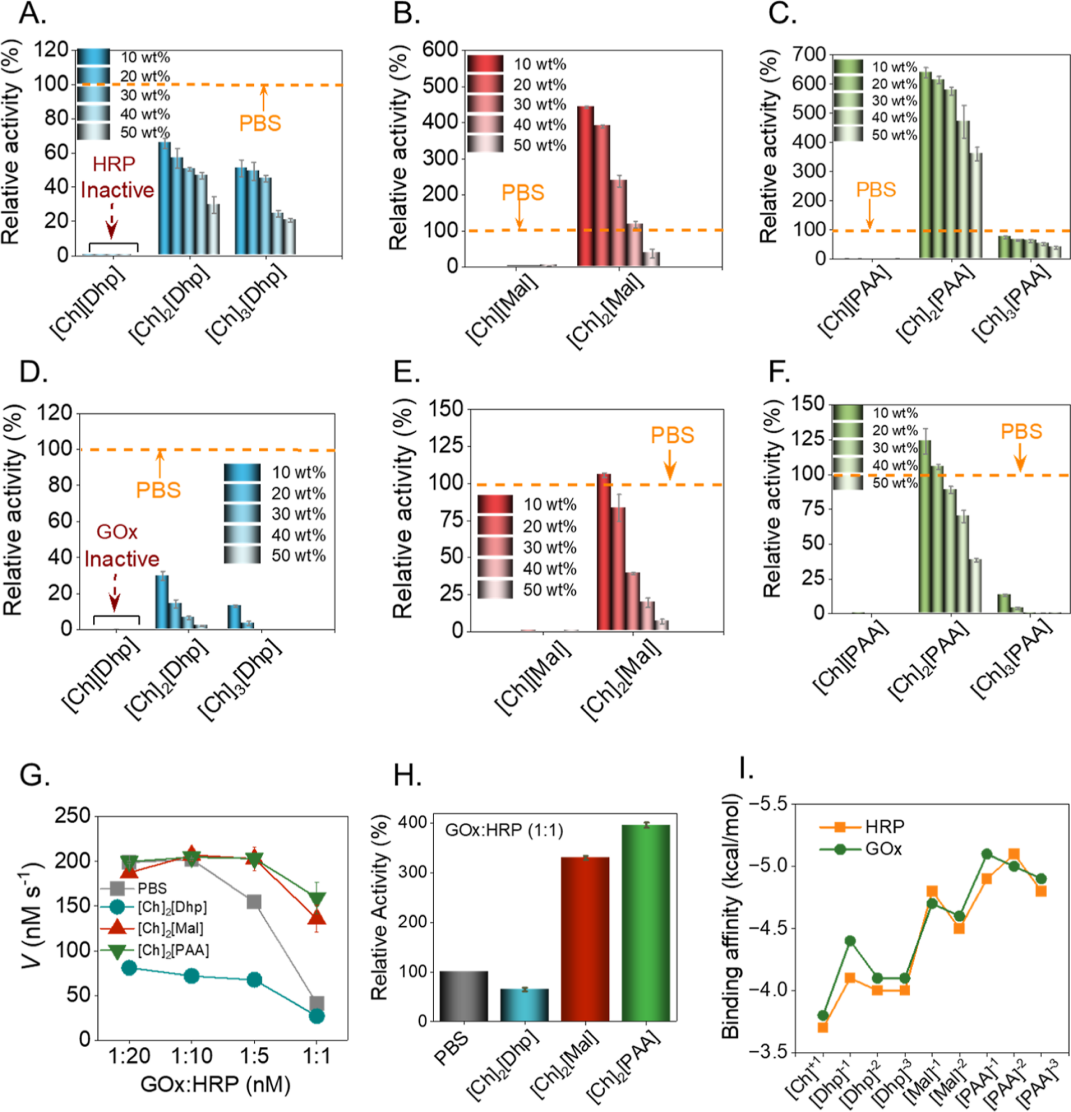
Relative activity of individual enzymes viz HRP and GOx and GOx–HRP tandem biocatalysis in all ILs. (A–C) Relative activity plots of HRP in various ILs such as [Ch]­[Dhp], [Ch]_2_[Dhp], and [Ch]_3_[Dhp] (A); [Ch]­[Mal] and [Ch]_2_[Mal] (B); and [Ch]­[PAA], [Ch]_2_[PAA], and [Ch]_3_[PAA] (C). (D–F) Relative activity plots of GOx in various ILs as represented by [Ch]­[Dhp], [Ch]_2_[Dhp], and [Ch]_3_[Dhp] (D); [Ch]­[Mal] and [Ch]_2_[Mal] (E); [Ch]­[PAA], [Ch]_2_[PAA], and [Ch]_3_[PAA] (F). In all the above cases, the standard PBS (pH 7.4) was taken as a control and considered as 100% activity; the relative activity in ILs was calculated accordingly. (G) Velocity of the GOx–HRP tandem biocatalysis with a different GOx–HRP ratio (1:20, 1:10, 1:5, and 1:1) in all the optimized ILs (10 wt %) [Ch]_2_[Dhp], [Ch]_2_[Mal], and [Ch]_2_[PAA]. (H) Relative activity plot GOx–HRP (1:1) cascade in various optimized 10 wt % ILs. (I) The binding affinities of top ranked docked poses for individual IL cations and anions are represented for GOx and HRP. The docking was performed using Autodock Vina for three protonation states of HRP and GOx at pH 2.3, 5, and 7 to understand the interaction preferences for specific IL.

Following this, the activity of GOx was monitored. The GOx activity assay was carried out in a solution comprising 1 nM GOx, 20 nM HRP, 100 mM d-glucose, and 2 mM ABTS (Supporting Information, Section S4.2). Similar to HRP, GOx also remained inactive in all the ILs having a single choline cation; [Ch]­[Dhp], [Ch]­[Mal], and [Ch]­[PAA]. The significantly low pH coupled with higher positive ESP values for GOx and HRP (Figure 1E,F) could be the reason for their inactivity, as it can induce the unfolding of the enzymes. ILs with a 2:1 molar ratio of [Ch]^+^ to anion—[Ch]_2_[Dhp], [Ch]_2_[Mal], and [Ch]_2_[PAA] at 10 wt % showed higher GOx activity. Among these, [Ch]_2_[PAA] (10 wt %) was found to be the best media with a relative activity of 123.74 ± 9.1% (Figure 2D–F). However, as the IL concentration increased above 10 wt %, a steady deterioration in GOx activity was observed across all ILs. A similar trend was also observed for ILs with a 3:1 [Ch]^+^ to anion ratio. In general, both GOx and HRP showed maximum activity at 10 wt % of [Ch]_2_[Dhp], [Ch]_2_[Mal], and [Ch]_2_[PAA], suggesting that this particular concentration provides a favorable microenvironment for enzyme activity likely due to an optimal pH range (4.8 to 7.0). Based on these findings, 10 wt % was selected as the optimal concentration for enzymatically active ILs to monitor the GOx–HRP ECR activity.

### GOx–HRP ECR in ILs

Before setting up the ECR in ILs, we reviewed various pros and cons that affects the ECR kinetics. (1) Reports show that the lag phase before reaching steady state poses a significant obstacle to achieving optimal cascade reaction kinetics.^39^ This issue can be addressed by improving the substrate channeling and modulating enzyme proximity.^12,39^ (2) Enzyme ratios in ECR are another critical factor that influence the overall efficiency and intermediate flux. Therefore, ECRs are usually constructed with a higher ratio of enzyme that leads to substrate channeling to omit the lag phase and minimizes diffusion losses.^39^ (3) However, high enzyme loading results in the overall ECR process being costly and thus achieving a higher ECR rate with a less enzymatic ratio is desirable. Therefore, to setup the economically best performing ECR in ILs, the efficiency of the GOx–HRP ECR was evaluated at varying molar ratios of GOx–HRP (1:20, 1:10, 1:5, and 1:1), while maintaining a constant GOx concentration of 1 nM (Figure S11). A comparison of the ECR velocities across different enzyme ratios revealed a pronounced decrease in catalytic efficiency in PBS and [Ch]_2_[Dhp] media (Figure 2G). Among the tested ILs, [Ch]_2_[PAA] emerged as the most effective medium with a 285% higher ECR velocity as compared to PBS even at a 1:1 ratio of GOx to HRP, suggesting the initial lag phase in this system was minimal (Figure 2H). The observed ECR velocity trend follows the order; ([Ch]_2_[PAA] ≥ [Ch]_2_[Mal] ≫ PBS ≥ [Ch]_2_[Dhp]). This suggests that in addition to pH, the nature of the IL anion significantly influences enzyme interactions in an ECR system. These results prompted further investigation of the change in the secondary structure and microenvironment of enzymes in each IL. To obtain a detailed molecular level understanding of ECR reaction mechanism in the used ILs, molecular docking, spectroscopy, and molecular dynamics studies were performed to establish the enzyme–stability–activity relationship.^40^

### Molecular Insights into Enzyme–IL Interactions and Enzyme Structural Stability

First, the binding affinities of IL moieties ([Ch]^+^, [Dhp]^−^, [Dhp]^2–^, [Dhp]^3–^, [Mal]^−^, [Mal]^2–^, [PAA]^−^, [PAA]^2–^, and [PAA]^3–^) with enzymes (HRP and GOx) in different protonation states were investigated using the AutoDock Vina program.^41,42^ The docking results summarized in Tables S2–S10 depict the differences in the electrostatic potential which assisted in the understanding of preferential binding of IL moieties at enzyme surfaces. Docking results revealed different binding affinities of diverse anionic charges of ILs, wherein the binding affinity of [Dhp]^–^ was found to be higher than [Dhp]^2–^ and [Dhp]^3–^ for both GOx and HRP (Figure 2I). Similarly, the binding affinity of [Mal]^–^ was more than [Mal]^2–^ with GOx and HRP. However, the binding affinities of [PAA]^2–^ with HRP and GOx were higher than [PAA]^–^ and [PAA]^3–^. Among the different anionic counterparts (Dhp, Mal, or PAA), the highest binding affinities were observed upon docking with [PAA]^−^, [PAA]^2–^, and [PAA]^3–^ anions for both the GOx and HRP. The binding affinities can be further ranked as [PAA] > [Mal] > [Dhp] for the GOx and HRP molecular systems (Tables S2–S10). [Ch]^+^ interacts with the His170 residue of HRP as well as with Tyr30, Phe564, His170, and Glu50 residues of GOx (Figures 3A and S12). The binding poses of HRP with the highest affinity, as facilitated by [PAA]^−^, [PAA]^2–^, and [PAA]^3–^, are similar, with consistent polar interactions involving residues such as Lys174 and Rrg31 (Figures 3B,C and S13–S15). Similar polar interactions were observed with HRP and other IL moieties such as [Mal]^−^, [Mal]^2–^, [Dhp]^−^, [Dhp]^2–^, and [Dhp]^3–^ (Figures 3D,E and S16–S20). Studies revealed that Lys174 was targeted to modulate the thermal stability of the HRP enzyme.^43^ For GOx, the polar residues involved in interactions vary across [PAA]^−^ to [PAA]^3–^, [Mal]^−^ to [Mal]^2–^ (Figures 3B–D and S13–S17). Ser291 near the FAD-binding domain of GOx made the most favorable interactions with [PAA]^2–^ and [Mal]^2–^, while for [Dhp]^2–^, His158, Arg176, Gly347, and Phe215 are the highest binding polar residues in GOx (Figure 3E).

**Figure 3:**
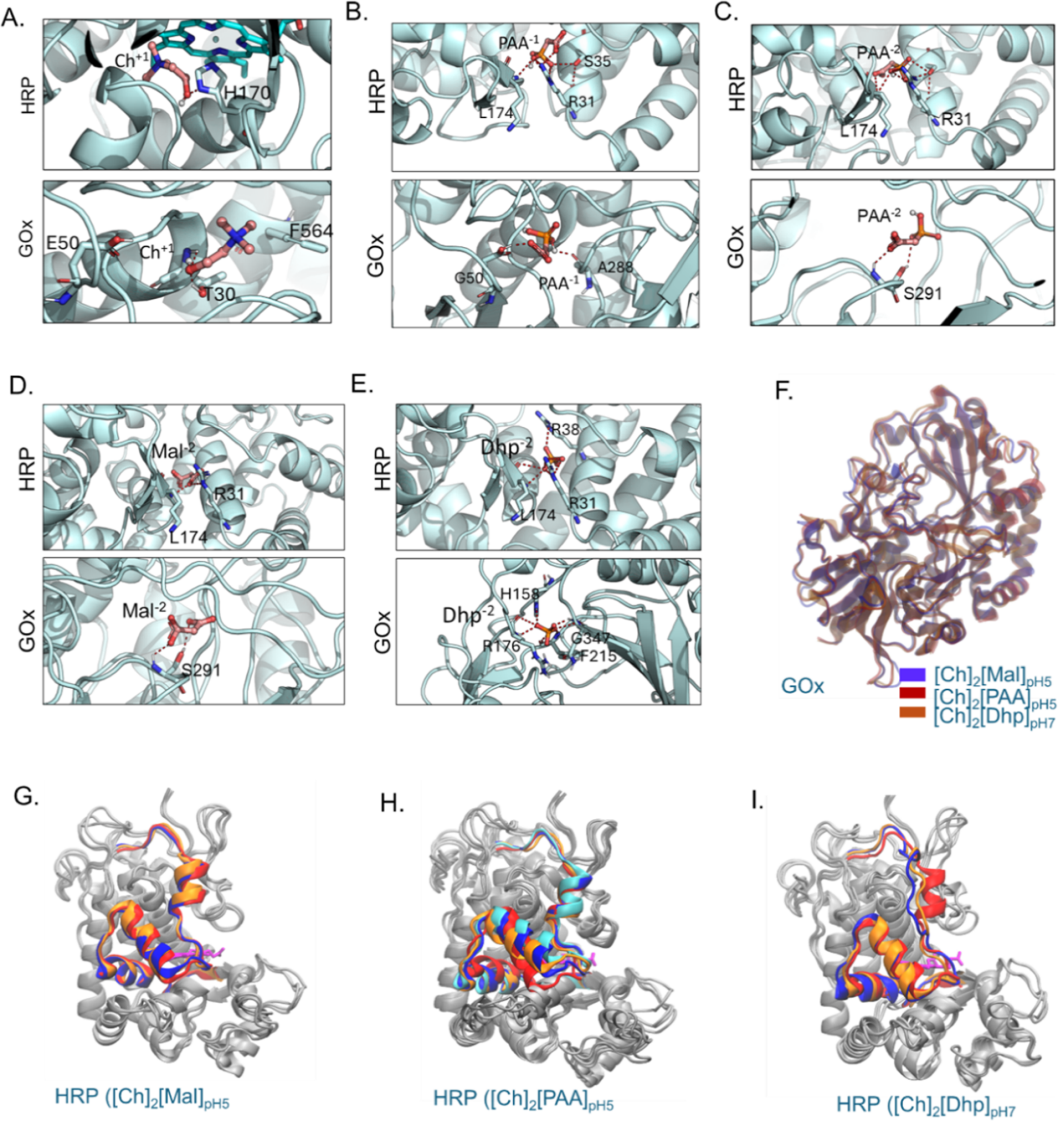
(A) The zoomed-in configuration represents the docked pose with the highest affinity binding sites of HRP and GOx, respectively, with Ch^+^. (B) The highest affinity binding sites of PAA^–^ and the pocket for HRP and GOx. (C) The highest affinity binding sites of PAA^2–^ and the pocket for HRP and GOx. (D) The highest affinity binding sites of Mal^2–^ and the pocket for HRP and GOx. (E) The highest affinity binding sites of Dhp^2–^ and the pocket for HRP and GOx. (F) Representative frames of GOx based on RMSD-based clustering depicting the regions with higher dynamics located near the catalytic site of GOx. (G,H) Representative frames of HRP based on RMSD-based clustering depicting the regions with higher dynamics located near the catalytic site of HRP in different ILs such as [Ch]_2_[Mal] (G), [Ch]_2_[PAA] (H), and [CH]_2_[Dhp] (I). The representative frames and highly dynamic regions (near residues 130–180) from different clusters are shown in red, orange, blue, and cyan and all other regions are shown in gray.

The pH-induced charge variations (Figure 1E,F), especially in the catalytic regions of HRP, influence the binding affinities of ILs, with HRP showing more energetically favorable interactions with certain ILs, such as [PAA]^2–^, compared to GOx. Considering the stabilizing effect posed by 2:1 IL composition, we performed unbiased molecular dynamics (MD) simulations at 2:1 ratio ILs to understand their effects on the structural and dynamics of GOx and HRP. RMSD results suggest that [Ch]_2_[PAA] and [Ch]_2_[Dhp] exerted a much better stability effect to the GOx (Figure S21a), on the other hand, [Ch]_2_[Mal] and [Ch]_2_[PAA] solvent mixtures were seen to exert more structural stability to HRP (Figure S21b). RMSD-based clustering conformations of HRP represent the regions with a dynamic microenvironment located near the catalytic site of HRP (Figure 3G–I), whereas no such prominent differences were observed for structural dynamics of GOx (Figure 3F). The higher RMSD of HRP further suggests that there were some reversible openings of the region near the catalytic site of the enzyme (Figures 3G–I and S21c). Especially, it was observed that the ILs could stabilize specific functional regions of HRP such as residue region 125–175. These residues are known to gate the active site and restrict substrate access to the heme catalytic center.^43^ Additionally, H_2_O_2_ is known to penetrate through fluctuating regions, particularly at Phe68 and Phe142.^43^ However, this stabilization effect was not observed for GOx, where the ILs were found to stabilize all regions (Figures 3F and S21d). RMSF results suggest that the functional stability of HRP was most prominent in the 125–175 residue region in the 2:1 [Ch]_2_[Dhp] and [Ch]_2_[PAA] ILs (Figure S21c). This observation aligns with the RMSD analysis, where the overall dynamics of the structures were well-maintained. Collectively, the MD results suggest that the nature of the anion (Dhp, Mal, and PAA) and pH conditions can strongly influence enzyme stability and flexibility, with [Ch]_2_[PAA] generally inducing greater conformational fluctuations in HRP, which is crucial for accessing the active site. In contrast, GOx exhibited a more uniform stabilization across all of the regions.

The alterations in the enzyme structure in response to variations in the solvent microenvironment have been determined by observing changes in their secondary structure conformations using CD spectroscopy. Furthermore, the alterations in the solvation-induced microenvironment were examined from second derivative UV–vis spectra (d^2^ *A*/dλ^2^) of tyrosine and tryptophan residues within proteins.^44,45^ The absorption spectra of Tyr and Trp in both HRP and GOx exhibit peaks at 285 and 295 nm, respectively.^45,46^ In 1:1 ILs [Ch]­[Dhp], [Ch]­[Mal], and [Ch]­[PAA]), a marked decrease in the derivative absorbance at 285 nm (d^2^ *A*/dλ^2^) was observed (Figure S22a), which implies a drastic alteration in the HRP microenvironment due to increased protein solvation.^45^ For GOx, ILs, [Ch]­[Dhp], and [Ch]­[PAA] induced a hypsochromic shift of absorbance corresponding to Tyr and Trp, indicating an increase in microenvironmental polarity around proteins upon solvation with IL ions (Figure S22b).^37^ In contrast, other ILs with higher choline cations showed minimal solvation of enzymes due to IL–IL interactions, which is in line with retention of active enzyme conformation (Figure 2). Further analysis of the d^2^ *A*_Tyr_/d^2^ *A*_Trp_ values for HRP in 10 wt % of [Ch]­[Dhp], [Ch]­[Mal], and [Ch]­[PAA] as compared to other ILs showed enhancement of d^2^ *A*_Tyr_/d^2^ *A*_Trp_ values indicating reduced aromatic packing and significant unfolding of HRP (Figure 4A).^45^ The GOx also displayed similar behavior of d^2^ *A*_Tyr_/d^2^ *A*_Trp_ values for 10 wt % of [Ch]­[Dhp] and [Ch]­[PAA] (Figure 4B). We also analyzed the change in the absorbance of the Soret band of HRP at 409 nm. The Soret band showed a drastic hypochromic shift in the presence of 10 wt % of [Ch]­[Dhp], [Ch]­[Mal], and [Ch]­[PAA] due to the change in solvent micropolarity around the heme cleft (Figure S23). These results indicate a significant alteration in the structure of HRP and GOx in [Ch]­[Dhp], [Ch]­[Mal], and [Ch]­[PAA] ILs, which is consistent with the negligible activity of HRP in these ILs. To further confirm the structural alterations in enzymes, we analyzed their secondary structure from CD spectroscopy.

**Figure 4:**
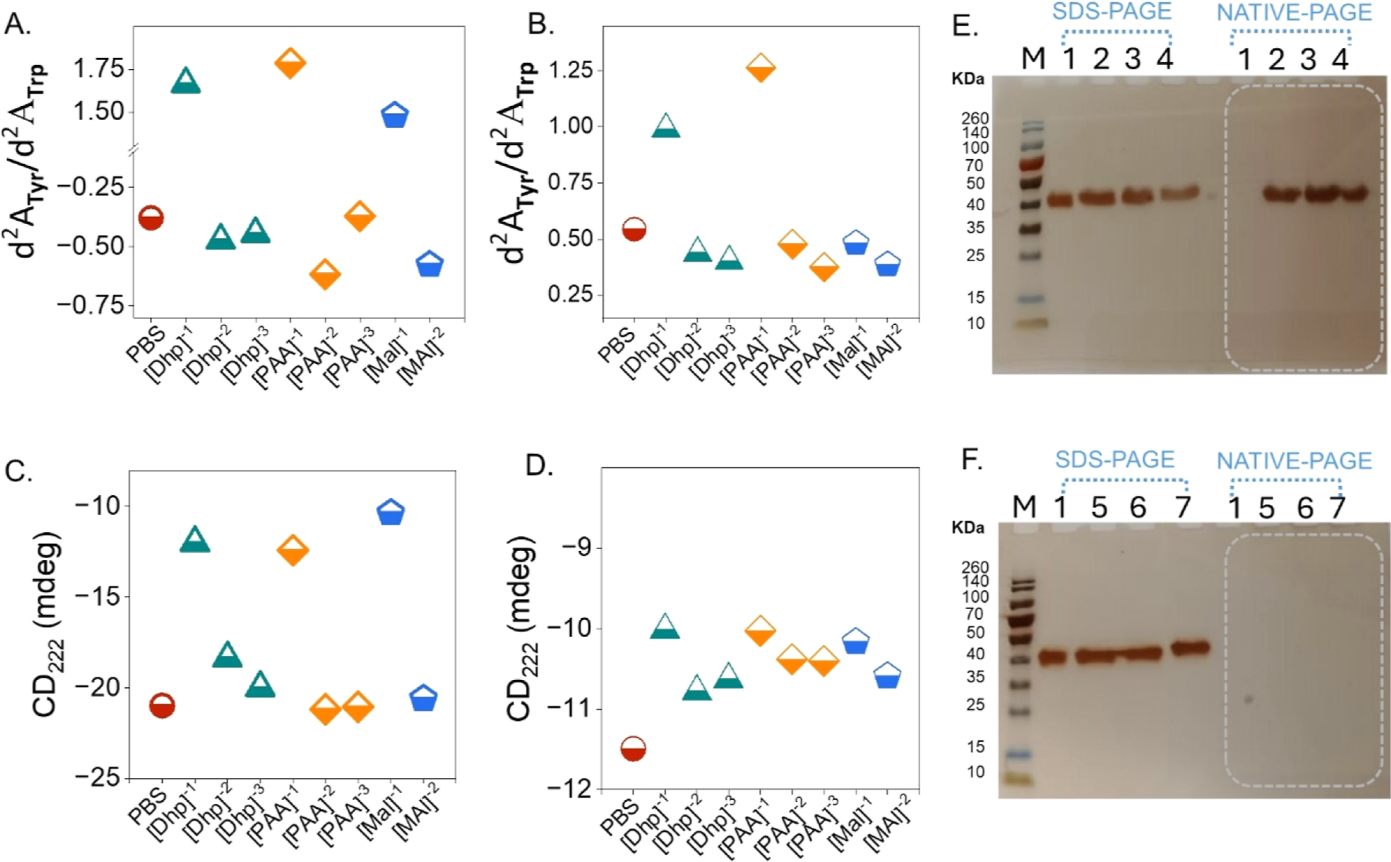
(A) The second derivative of the tyrosine to tryptophan ratio (d^2^ *A*_Tyr_/d^2^ *A*_Trp_) plot of HRP in all IL media. (B) The second derivative of the tyrosine to tryptophan ratio (d^2^ *A*_Tyr_/d^2^ *A*_Trp_) plot of GOx in all IL media. Enzyme concentration for both HRP and GOx was fixed to 0.125 mg/mL, and IL concentration was 10 wt %. (C) The plot of CD value at the peak 222 nm vs ILs, including PBS (pH 7.4) for HRP. (D) Peak at 222 nm of GOx vs ILs. Enzyme concentration for both HRP and GOx was fixed to 0.125 mg/mL, and IL concentration was 1 wt %. (E,F) Reduced and native polyacrylamide gel electrophoresis (PAGE) of HRP and GOx in different ILs (10 wt %) and PBS (pH 7.4). (E) Image of reduced and native PAGE of HRP in (1:1) ILs. M-marker, 1-PBS, 2-[Ch]­[Dhp], 3-[Ch]­[Mal], and 4-[Ch]­[PAA]. (F) Reduced and native PAGE image of HRP in (2:1) ILs. 1-PBS, 5-[Ch]_2_[Dhp], 6-[Ch]_2_[Mal], and 7-[Ch]_2_[PAA].

The CD spectra of HRP and GOx clearly showed a negative absorption band at ellipticity minima of 209 and 222 nm (−θ_209nm_ and −θ_222nm_), characteristic of all-α secondary structures (Figure S24).^47^ HRP showed a significant decrease in both −θ_209nm_ and −θ_222nm_ bands in the presence of [Ch]­[Dhp], [Ch]­[Mal], and [Ch]­[PAA], indicating a significant decrease in the α-helical content of HRP (Figures 4C and S24a).^47^ This result corroborates with the d^2^ *A*/dλ^2^ and activity results, thus demonstrating structure–function correlation of HRP in these ILs. This is also consistent with the fact that low pH solutions cause protein denaturation, a phenomenon well exploited to induce protein fibrillation.^38^ Interestingly, the secondary structure of HRP showed a minimal change at 10 wt % of other ILs with a higher ratio of choline cation, which supports the HRP activity in these ILs. Unlike HRP, the secondary structures of GOx remained largely intact in all of the studied ILs, as indicated by minimal changes in −θ_209nm_ and −θ_222nm_ (Figures 4C and S24b), which is also consistent with the UV–vis results (Figure 4B). Therefore, the UV–vis and CD results confirmed that ILs with two choline cations (2:1 molar ratio) provide a more stable microenvironment to the studied enzymes. Hence, further studies were carried out with ILs having a 2:1 molar ratio of choline:anion.

Further, we utilized denaturing SDS-PAGE (with β-mercaptoethanol) and native PAGE assay to confirm the stability of both the enzyme (HRP and GOx) dissolved in ILs (10 wt %) (Figures 4E,F and S25). Enzymes dissolved in PBS were used as a control. Both reduced and native PAGE assays show similar migration rates of HRP dissolved in [Ch]­[Dhp], [Ch]­[Mal], and [Ch]­[PAA] (Figure 4E), whereas no band was observed in the native PAGE assay for HRP when dissolved in PBS, [Ch]_2_[Dhp], [Ch]_2_[Mal], and [Ch]_2_[PAA] (Figure 4F). GOx is a dimeric protein (160 kDa), which upon denaturation undergoes monomerization into two subunits (80 kDa). The GOx monomers migrate at a faster rate than the dimer in the PAGE setup.^19^ The SDS and native PAGE of GOx dissolved in PBS and [Ch]­[Dhp], [Ch]­[Mal], and [Ch]­[PAA] showed denaturation (Figure S25). Whereas, GOx dissolved in [Ch]_2_[Dhp], [Ch]_2_[Mal], and [Ch]_2_[PAA] displayed no denaturation, indicated by a single subunit (dimeric form). These results further confirm that ILs, [Ch]­[Dhp], [Ch]­[Mal], and [Ch]­[PAA] denature, whereas [Ch]_2_[Dhp], [Ch]_2_[Mal], and [Ch]_2_[PAA] (2:1) stabilize both HRP and GOx when dissolved in 10 wt % of these ILs in water.

### Mechanistic Understandings toward Improved ECR Kinetics in ILs

Further mechanistic insights of improved ECR kinetics in the studied ILs were obtained by investigating the dynamic catalytic activities of HRP, GOx, and GOx–HRP cascades at varying substrate concentrations (see the Experimental Section for details). For this, 10 wt % solutions of ILs ([Ch]_2_[Dhp], [Ch]_2_[Mal], and [Ch]_2_[PAA]) were chosen as media because of their demonstrated stabilizing action toward these enzymes. The plots showing reaction velocity vs substrate concentration for HRP, GOx, and GOx–HR ECR cascades are shown in Figures S26–S28. Furthermore, we also calculated the *V*_max_, *K*_m_, *K*_cat_, and *K*_eff_ (*K*_cat_/*K*_m_) using three standard enzyme kinetics models: M–M, L–B, and E–H to validate the obtained data with maximum accuracy (Figures S26–S28 and Tables S11–S22). The results of *V*_max_, *K*_m_, and *K*_cat_ of GOx, HRP, and GOx–HRP cascades suggested that all three kinetic models well fitted to the kinetic parameters. We observed a noticeable scaling between the *K*_m_ and *K*_cat_, and it could be expressed as a linear free energy relationship (LFER).^48,49^ Accordingly, a power-law correlation between the normal logarithms of the kinetic parameters (*K*_m_, and *K*_cat_) was established. The dynamic catalytic activity of HRP is enhanced in the presence of ILs, [Ch]_2_[Mal] and [Ch]_2_[PAA], when compared with that of [Ch]_2_[Dhp] and PBS (Figure 5A). However, in the case of GOx, only [Ch]_2_[PAA] exhibited a significant enhancement in activity compared to PBS (Figure 5B). In both enzymes, the kinetics data followed a LFER for various ILs and PBS. In contrast, the GOx–HRP ECR kinetics deviated from LFER (Figure 5C), driven by a marked reduction in the apparent *K*_m_ values observed in [Ch]_2_[PAA] and [Ch]_2_[Mal] compared to [Ch]_2_[Dhp] and PBS. This nonlinear response suggests an improved substrate affinity in these IL environments.^48,49^ This result is consistent with the conventional knowledge that enzyme kinetics can be accelerated by stronger ground-state substrate binding, which lowers *K*_m_ and increases *K*_cat_, thereby enhancing the kinetic efficiency.^50^ Notably, the GOx–HRP cascade in [Ch]_2_[PAA] demonstrated this effect most effectively. We further investigated the dynamic property of the enzymatic reactions in different ILs by plotting Ln­(*K*_cat_/*K*_cat‑ref_) vs Ln­(*K*_m_/*K*_m‑ref_) (Figure 5D–F). The obtained value of control (PBS pH 7.4) was set to zero, and for [Ch]_2_[Dhp], the obtained value was similar to PBS for HRP, GOx, and GOx–HRP cascade systems (Figure 5D–F). Remarkably, the GOx–HRP ECR system did not show a linear correlation (Figure 5F); however, an increase in Ln­(*K*_cat_/*K*_cat‑ref_) was observed in [Ch]_2_[PAA] and [Ch]_2_[Mal], while [Ch]_2_[PAA] also displayed a marked decrease in the Ln­(*K*_m_/*K*_m‑ref_). These findings further suggest an enhanced substrate affinity and catalytic efficiency along with a reduced kinetic barrier, particularly in the presence of [Ch]_2_[PAA].

**Figure 5:**
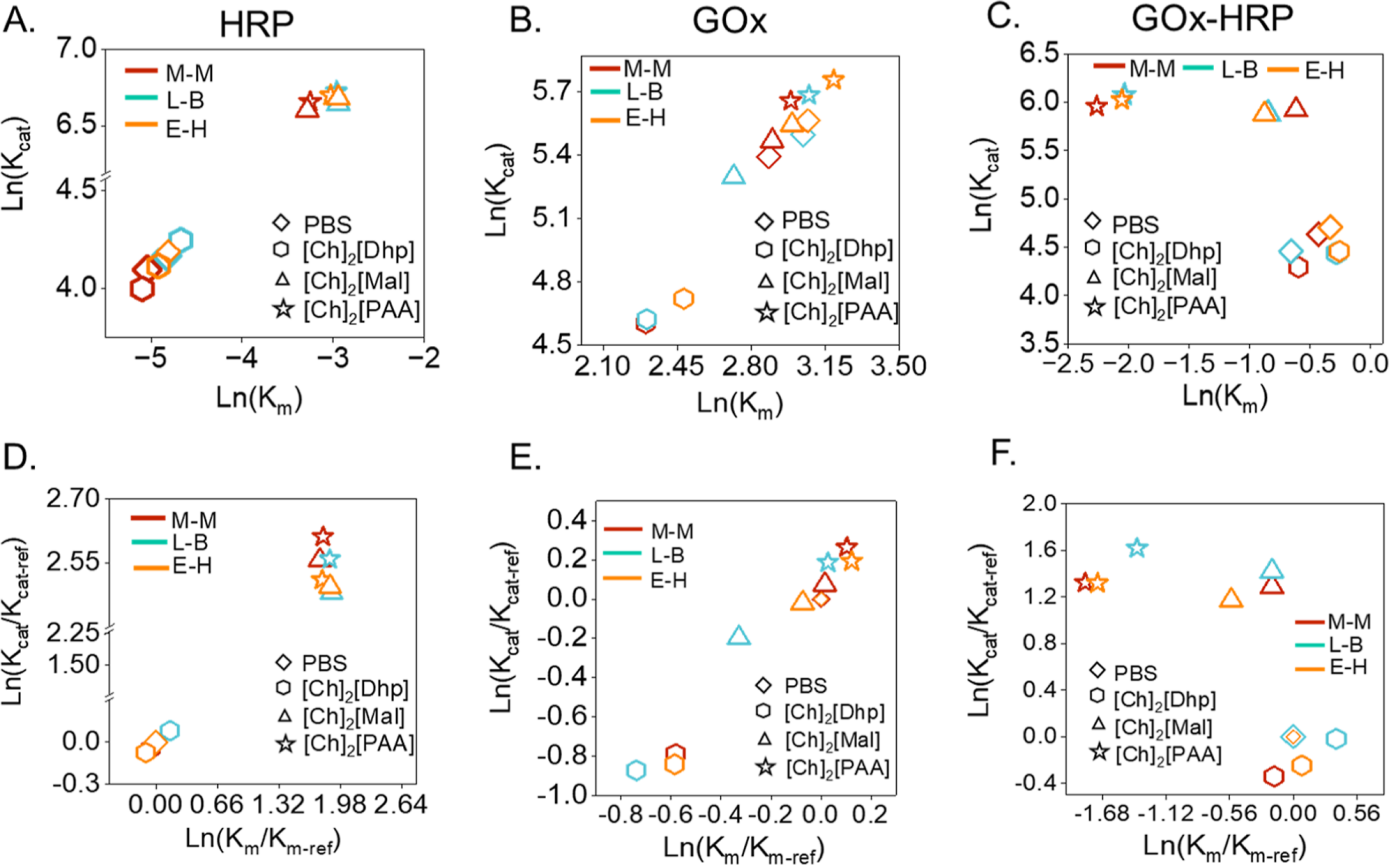
(A–C) Kinetic parameter *K*_m_ vs *K*_cat_ plotted with a normal logarithmic value with different kinetic model fittings. (A) The Ln­(*K*_cat_) vs Ln­(*K*_m_) plot for the HRP catalyzed reaction. (B) The Ln­(*K*_cat_) vs Ln­(*K*_m_) plot for GOx. (C) The Ln­(*K*_cat_) vs Ln­(*K*_m_) plot for GOx–HRP cascade reactions. (D–F) Comparison of kinetic parameters *K*_cat_, *K*_m_, and *K*_cat_/*K*_m_ was shown with a normal logarithmic value with different kinetic model fittings. (D–F) Ln­(*K*_cat_/*K*_cat‑ref_) vs Ln­(*K*_m_/*K*_m‑ref_) plot of HRP, GOx, and GOx–HRP cascade reactions, respectively.

The free energy of substrate binding affinity to the enzymes was experimentally evaluated using *K*_m_ and *K*_cat_ values using eqs 6 and 7. The transition-state free energy (ΔΔ*G* ^⧧^) and standard free energy of ligand binding (ΔΔ*G*_B_^o^) of the HRP, GOx, and GOx–HRP cascade reactions are obtained using eqs 6 and 7.^49–51^

6 $$\Delta \Delta G_{B}^{o}=RT\;\ln\left(\frac{K_{m}}{K_{m‐ref}}\right)$$

7 $$\Delta \Delta G^{‡}=-RT\;\ln\left(\frac{K_{cat}}{K_{cat‐ref}}\right)$$

As the *K*_m_ and free energy of ligand binding are inversely proportional to each other, increasing *K*_m_ leads to lesser binding reflected and vice versa (Figure 6A–C). The plot of ΔΔ*G* ^⧧^ vs ΔΔ*G*_B_^o^ for the HRP-catalyzed reaction was found to be linearly decreasing from [Ch]_2_[Dhp], PBS 7.4, [Ch]_2_[Mal] to [Ch]_2_[PAA] due to significantly higher *K*_cat_ and stabilization of transition-state but the ligand binding affinity was lower because of higher *K*_m_ values (Figure 6A). A similar observation was also noted for GOx activity in ILs, and from [Ch]_2_[Dhp], [Ch]_2_[Mal], PBS 7.4 to [Ch]_2_[PAA], a decreasing trend in ΔΔ*G* ^⧧^ was followed (Figure 6B) due to the higher *K*_cat_ value. Interestingly, for the GOx–HRP cascade system, due to higher *K*_cat_ and moderate *K*_m_ values, the ΔΔ*G* ^⧧^ becomes lower for [Ch]_2_[Mal], which signified stabilization of transition-state complex but due to a lower ligand binding affinity, as it did not show a significant decrease in ΔΔ*G*_B_^o^. Contrary to [Ch]_2_[Mal], a higher transition state stability and ligand binding affinity was concluded for [Ch]_2_[PAA] as we observed a significant decrease in both ΔΔ*G*_B_^o^ and ΔΔ*G* ^⧧^ (Figure 6C). The catalytic efficiency, derived as *K*_cat_/*K*_m_, is correlated with data obtained from the M–M, L–B, and E–H kinetic models and is associated with the ΔΔ*G*_B_^o^ and ΔΔ*G* ^⧧^. The HRP-catalyzed reaction was observed with a higher catalytic efficiency of about 2-fold for both [Ch]_2_[Mal] to [Ch]_2_[PAA] compared to that of the control PBS (7.4) and [Ch]_2_[Dhp] (Figure 6D). The kinetic efficiency of GOx was also noted, with an increase in *K*_cat_/*K*_m_ up to 1.2 times for [Ch]_2_[PAA] (Figure 6E). The cascade reaction in PBS (pH 7.4) showed a catalytic efficiency of about 158 mM^–1^ s^–1^, which shoots up to around 3738 mM^–1^ s^–1^ in the [Ch]_2_[PAA] (Figure 6F). Although the individual enzymes were unsuccessful in showing a substantial enhancement of *K*_cat_/*K*_m_, the GOx–HRP cascade resulted with about 25-fold enrichment of *K*_cat_/*K*_m_ in [Ch]_2_[PAA] IL. Such an enrichment in the *K*_cat_/*K*_m_ of the GOx–HRP ECR competes with the highest activities reported to date (Table S23). Previous studies have largely focused on complex designs that modulate the spatial proximity between GOx and HRP, while relatively less attention has been given to the dynamic modulation of the enzyme microenvironment surrounding the catalytic sites. Our results highlight that the enhanced ECR efficiency in [Ch]_2_[PAA] is facilitated by the contributions from lower binding energy, transition state stabilization, and local microenvironmental effects, which collectively enable the enzymes involved in the ECR system to realize their full catalytic potential.

**Figure 6:**
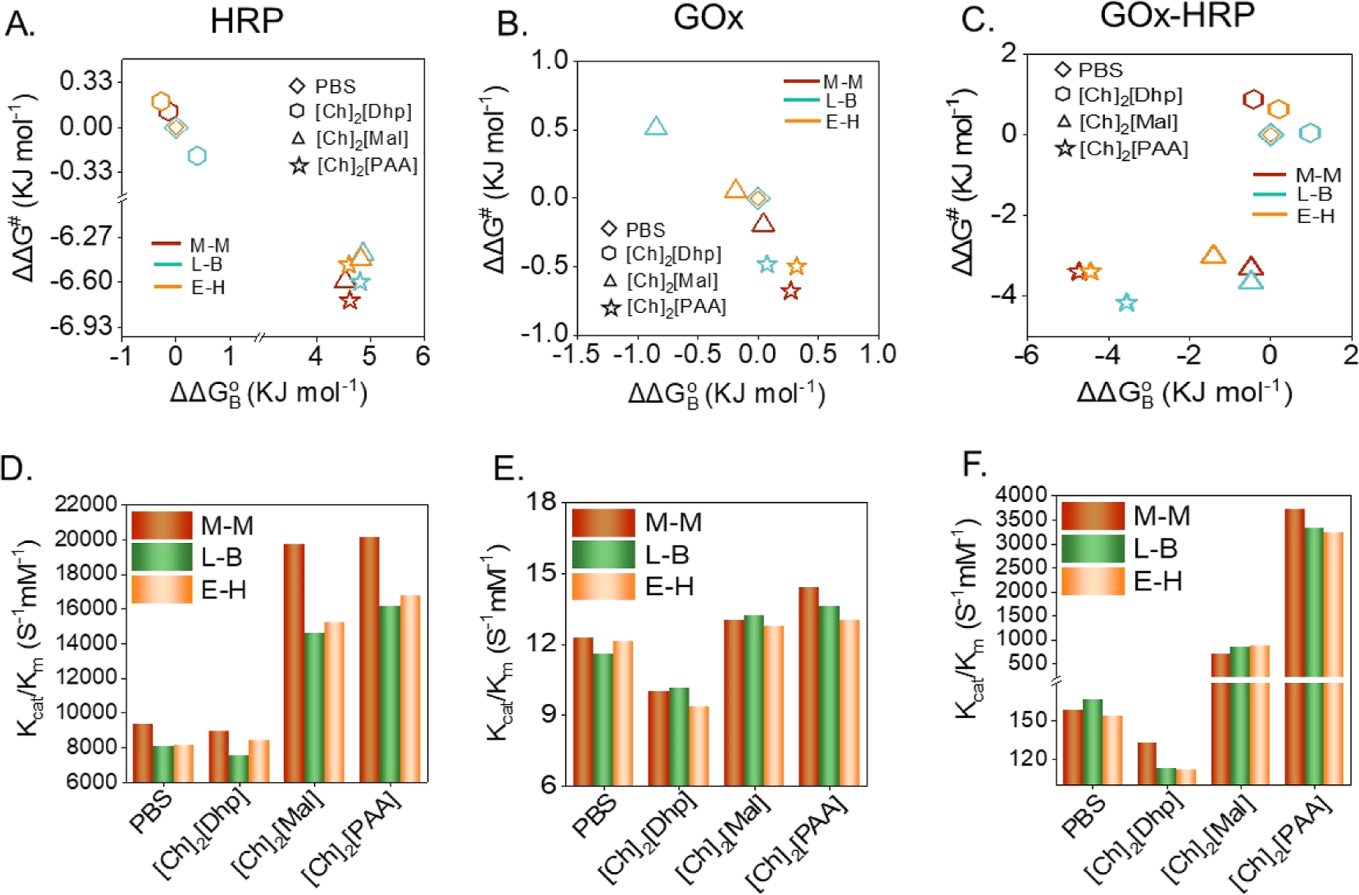
(A–C) The change in standard free energy of ligand binding (ΔΔ*G*_B_^o^) as well as transition-state free energy (ΔΔ*G* ^⧧^) of HRP, GOx, and GOx–HRP cascade reaction, respectively. (D–F) The comparison plots between *K*_cat_/*K*_m_ and various IL with different kinetic models for HRP, GOx, and GOx–HRP cascade reactions, respectively. All the kinetic parameters were individually evaluated for HRP, GOx, and GOx–HRP (1:1 mol ratio) cascades in [Ch]_2_[Dhp], [Ch]_2_[Mal], and [Ch]_2_[PAA] at optimized concentrations (10 wt %) and PBS (pH 7.4). We have validated the obtained kinetic data with four well-known kinetic models such as Michaelis–Menten (M–M), Lineweaver–Burk (L–B), and Eadie–Hofstee (E–H).

The improved kinetic efficiency of the GOx–HRP cascade in [Ch]_2_[Mal] and [Ch]_2_[PAA] prompted further investigation using molecular docking to understand the interactions with the IL component and GOx–HRP complex. To obtain a consistent model of the GOx–HRP complex, docking was performed using both HDOCK,^52^ and GRAMM (detailed methods provided in the Supporting Information).^53^ Although AlphaFold was additionally employed,^54^ the low score (ipTM = 0.35) indicated poor confidence in the predicted assembly; therefore, only physics-based docking approaches were followed. Analysis of the top ten poses from each tool revealed that both tools consistently placed GOx near the HRP active site, although the orientations were not identical. Pose 2 from HDOCK was ultimately selected for further investigation based on the docking score, overlap with alternative poses, and favorable interactions (Figure 7A). The HRP active site residues (Arg38, His42, and His170) are positioned in the favorable orientation to the catalytic triad of GOx (Glu412, His516, and His559) for the cascade reaction. The GOx–HRP complex was then used as the receptor for the docking of the common IL cation ([Ch]^+^) with three different anions: [PAA]^2–^, [Mal]^2–^, and [Dhp]^2–^. The resulting interaction diagrams are shown in Figure 7B–E. The docking results suggest that the anion component plays a more dominant role than the cation in modulating interactions with the GOx–HRP complex. Specifically, the binding modes indicate that the anions are capable of forming H-bonding networks in proximity to the HRP heme site, potentially stabilizing catalytically favorable conformations. Such stabilization could limit local structural fluctuations and help preserve the GOx–HRP complex efficiency under reaction conditions. Among the anions, [PAA]^2–^ and [Mal]^2–^ displayed preferential binding in the HRP active site. Common interactions were observed with Arg31, Ser35, and Lys174, with [PAA]^2–^ additionally forming a hydrogen bond with Arg38 (Figure 7B). In contrast, the smaller [Dhp]^2–^ anion failed to engage the backbone nitrogen of Lys174 (Figure 7D). [Ch]^+^ showed a different preference, largely binding at the GOx–HRP interface (with a docking score of −3.654 kcal/mol) or occupying the GOx pocket near the interface, rather than at the HRP active site (Figure 7E). Docking scores further reflected a similar trend as observed for GOx–HRP ECR velocity, with [PAA]^2–^ exhibiting the strongest binding energy (−5.076 kcal/mol), followed by [Mal]^2–^ (−4.452 kcal/mol) and [Dhp]^2–^ (−4.151 kcal/mol). These observations agree with experimental findings, supporting qualitatively that specific anion–residue interactions contribute to the stabilization of catalytically active conformations of the GOx–HRP complex.

**Figure 7:**
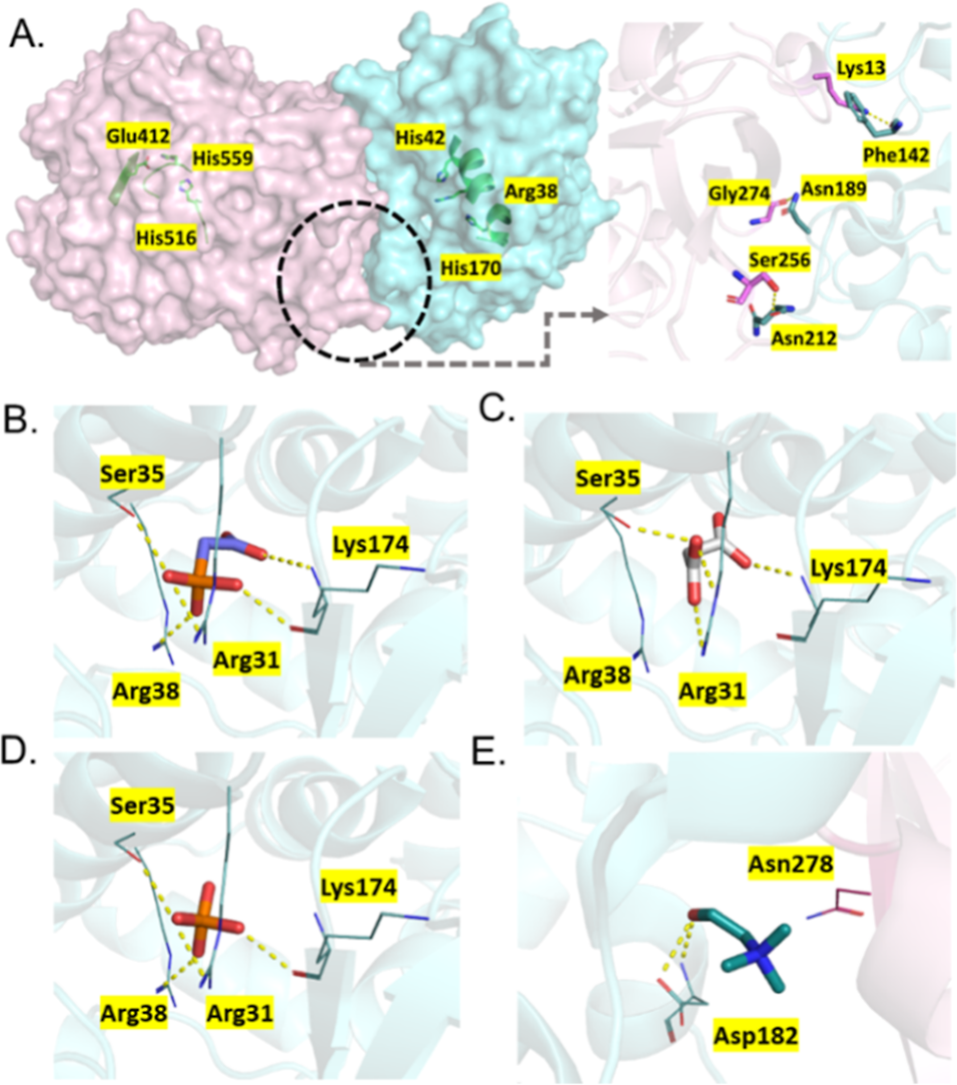
(A) Docked GOx–HRP complex shows GOx (pink) and HRP (cyan) with catalytic residues in green. The interface (black circle) highlights hydrogen bonds (yellow dashes) stabilizing the complex. (B–E) Binding modes of IL components within the GOx–HRP complex with [PAA]^2–^, [Mal]^2–^, [Dhp]^2–^, and [Ch]^+^, respectively. Residues are labeled in black with a yellow background and hydrogen bonds are indicated by yellow dashed lines.

### Improved Thermo-Stress Tolerance and Thermodynamic Stability of GOx–HRP in ILs

We also evaluated the thermal stability of HRP and the GOx–HRP enzymatic cascade over a temperature range of 37 to 80 °C (Figure 8A–C). In the presence of 10 wt % [Ch]_2_[PAA], both HRP and the GOx–HRP cascades exhibited the highest relative activities at 37 °C, reaching 403% and 377% (Figure 8A,B), respectively. Enzymatic activity declined gradually with increasing temperatures, where a significant loss was observed beyond 70 °C. The half-life temperature (*T*_50_), indicating the temperature corresponding to 50% of the initial enzymatic activity, was comparable for HRP between the control and the 10 wt % IL solutions. However, the GOx–HRP cascade exhibited a 16.3% increase in *T*_50_ in the presence of 10 wt % [Ch]_2_[PAA] (Figure 8C), indicating improved thermal stability relative to both PBS and other IL systems. The *T*_50_ follows the same trend of the melting temperature (*T*_m_) of HRP and GOx (Figure 8C). To explain the observed improvement in *T*_50_ of the GOx–HRP cascade, we examined the thermal stability of HRP and GOx using temperature-dependent (25 to 90 °C) CD spectroscopy (Figure 8D–I).

**Figure 8:**
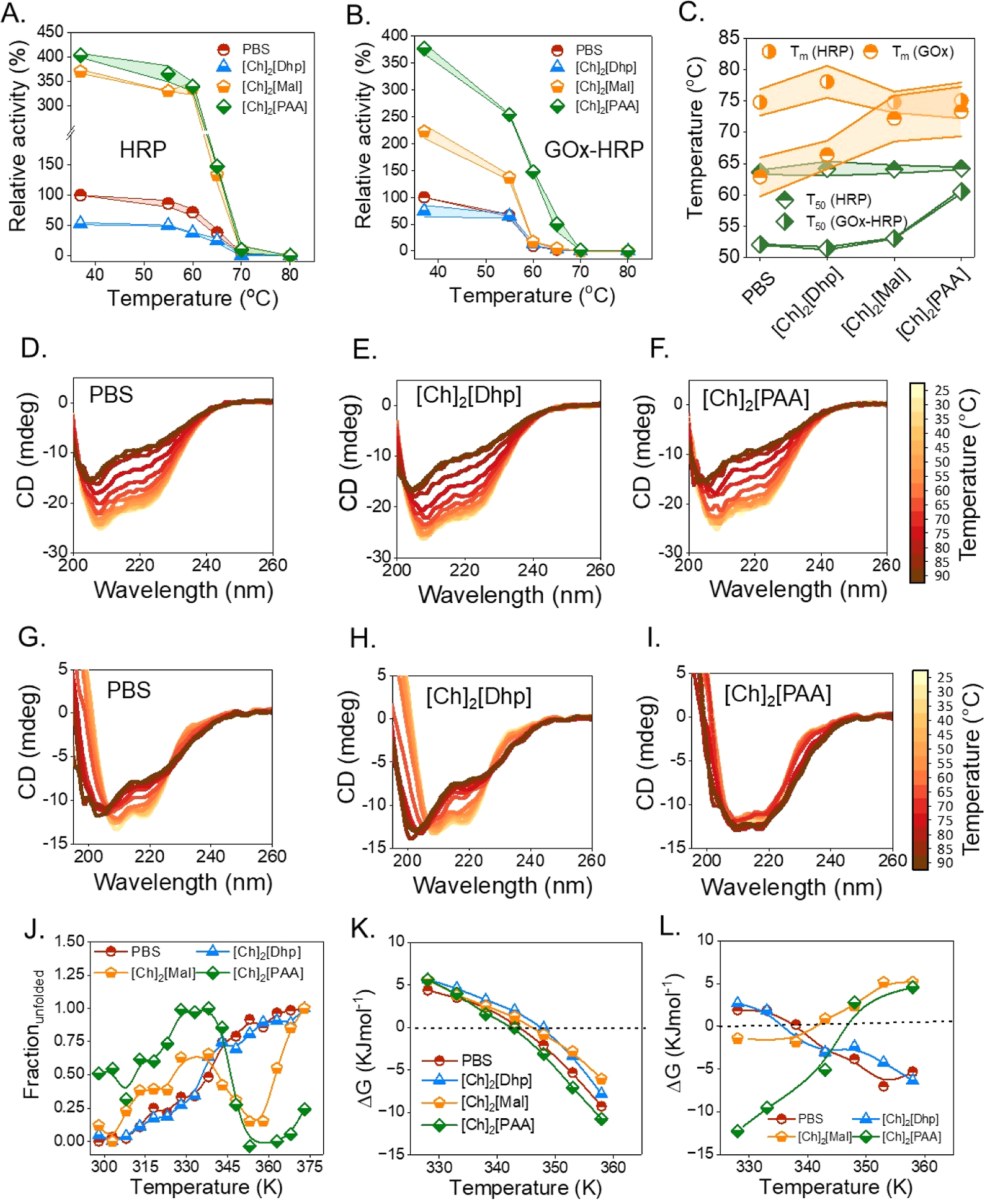
(A) Relative activity of HRP under thermal stress. PBS (pH 7.4) was taken as control media and relative activity was measured assuming the cascade activity in PBS as 100%. (B) The relative activity plot of GOx–HRP cascade in optimized 10 wt % ILs. (C) Melting temperature (*T*_m_) of HRP and GOx and half-life (*T*_50_) of HRP and GOx–HRP cascade system in ILs and PBS. (D–F) Temperature-dependent CD spectra within the temperature range 25–90 °C of HRP in PBS 7.4, [Ch]_2_[Dhp], and [Ch]_2_[PAA], respectively. (G–I) Temperature-dependent CD spectra within the temperature range 25–90 °C of GOx in PBS 7.4, [Ch]_2_[Dhp], and [Ch]_2_[PAA], respectively. (J) Fraction unfolded vs temperature for GOx in different solvents. (K,L) Stability curve (changes in free energy (Δ*G*) upon thermal denaturation) of HRP and GOx, respectively. For CD analysis, concentration of both the enzymes was fixed at 0.25 mg/mL and IL was 1 wt %.

The unfolding profiles of HRP in PBS and in all tested ILs showed similar temperature-dependent CD spectral patterns, indicative of a similar unfolding mechanism (Figures 8D–F and S29a). In contrast, GOx exhibited distinct unfolding behaviors depending on the solvent environment. While GOx unfolded likewise in PBS and [Ch]_2_[Dhp], markedly different unfolding patterns were observed in [Ch]_2_[PAA] and [Ch]_2_[Mal] (Figures 8G–I and S29b). Upon increasing the temperature beyond the melting temperature (*T*_m_) of HRP, the −θ_222nm_ diminished in a sigmoidal pattern across all solvents, consistent with protein denaturation via the loss of α-helix structure and simultaneous increase in β-sheet and random coil content (Figure S29c).^47^ A comparable denaturation profile was observed for GOx in PBS and [Ch]_2_[Dhp], whereas GOx in [Ch]_2_[PAA] and [Ch]_2_[Mal] exhibited a different unfolding mechanism (Figure S29d). To further understand the thermal stability, the *T*_m_ values of HRP and GOx were determined by plotting the fraction of unfolded protein versus temperature (Figures 8J and S29e). The *T*_m_ of HRP and GOx in PBS were 74.79 and 62.89 °C, respectively. Notably, the *T*_m_ of HRP remained mostly unaffected across all ILs. Conversely, GOx exhibited a significant increase in thermal stability in IL systems, with the highest *T*_m_ observed in [Ch]_2_[PAA] (73.31 °C), a 16.5% increase relative to PBS. This trend in *T*_m_ mirrors the enhancement in *T*_50_ values for both HRP and the GOx–HRP cascades (Figure 8C, Table S24). Further support for improved enzyme stability in ILs as investigated by the thermodynamic stability curves (Figure 8K,L, Table S25). The intersection points where Δ*G* = 0 denote the temperature at which folded and unfolded states are equally populated. HRP and GOx in PBS and [Ch]_2_[Dhp] exhibited similar slopes and crossed Δ*G* = 0 at lower temperatures than in [Ch]_2_[Mal] and [Ch]_2_[PAA], suggesting fast unfolding in the former environments. Overall, [Ch]_2_[Mal] and [Ch]_2_[PAA] induce alteration of GOx unfolding pathways and increase *T*_m_ values, which corroborates well with the improved half-life of GOx–HRP cascade stability, supporting their potential in robust tandem biocatalysis with accelerated activity and stability.

## Conclusions

In summary, this study demonstrates a transformative strategy to accelerate the ECR efficacy using pH-switchable ILs. In contrast to commonly studied methods that rely on complex scaffolds or proximity-based systems, pH-switchable ILs provide a versatile platform to enhance the biological activity of the multienzyme cascade with improved thermodynamic stability and tolerance to thermal stress. The significant improvement (25-fold) in the kinetics of the GOx–HRP cascade in 10 wt % [Ch]_2_[PAA] IL demonstrates the suitability of [Ch]_2_[PAA] for stabilizing multienzyme structures and facilitating catalytically active conformational states. The ability to tailor the microenvironment of multiple enzymes in a single IL suggests that the full potential of ECR could be realized in a cell-free system, opening a new avenue for efficient and sustainable biosynthetic cascade processes. In addition to improved tandem biocatalysis, this innovative approach could also lead to improvements in metabolic engineering and artificial cell design, where precise modulation of enzyme function is important.

## Supplementary Material



## Data Availability

Resources availability will be fulfilled by the lead contact, Dr. D. Mondal upon reasonable request.

## Notes

Detail of materials, characterizations, molecular docking, molecular modeling, HNMR, pH of ILs, HRP catalyzed reaction, GOx catalyzed reaction, GOx**-**HRP cascade reaction, docked scores, docking configurations, RMSF and RMSD plots, UV-Vis spectra, CD spectra, reduced and native gel electrophoresis, kinetic plots, kinetic parameters, analysis of thermal properties, and comparison of GOx-HRP kinetic and thermal stability (PDF)

## References

[1] Bornscheuer U. T., Huisman G., Kazlauskas R., Lutz S., Moore J., Robins K. (2012). Engineering the third wave of biocatalysis. Nature.

[2] Benítez-Mateos A. I., Roura
Padrosa D., Paradisi F. (2022). Multistep enzyme cascades as a route
towards green and sustainable pharmaceutical syntheses. Nat. Chem..

[3] Zhang Y., Tsitkov S., Hess H. (2018). Complex dynamics
in a two-enzyme
reaction network with substrate competition. Nat. Catal..

[4] Zhao X., Palacci H., Yadav V., Spiering M. M., Gilson M. K., Butler P. J., Hess H., Benkovic S. J., Sen A. (2018). Substrate-driven
chemotactic assembly in an enzyme cascade. Nat.
Chem..

[5] Zhao S., Kumar R., Sakai A., Vetting M. W., Wood B. M., Brown S., Bonanno J. B., Hillerich B. S., Seidel R. D., Babbitt P. C. (2013). Discovery
of new enzymes
and metabolic pathways by using structure and genome context. Nature.

[6] Stack T. M., Gerlt J. A. (2021). Discovery of novel
pathways for carbohydrate metabolism. Curr.
Opin. Chem. Biol..

[7] Kumar A., Wang L., Ng C. Y., Maranas C. D. (2018). Pathway design using
de novo steps through uncharted biochemical spaces. Nat. Commun..

[8] Chen X., Gao C., Guo L., Hu G., Luo Q., Liu J., Nielsen J., Chen J., Liu L. (2018). DCEO biotechnology:
tools to design, construct, evaluate, and optimize the metabolic pathway
for biosynthesis of chemicals. Chem. Rev..

[9] Shi J., Zang X., Zhao Z., Shen Z., Li W., Zhao G., Zhou J., Du Y.-L. (2023). Conserved Enzymatic
Cascade for Bacterial Azoxy Biosynthesis. J.
Am. Chem. Soc..

[10] Giannakopoulou A., Gkantzou E., Polydera A., Stamatis H. (2020). Multienzymatic nanoassemblies:
recent progress and applications. Trends Biotechnol..

[11] Vázquez-González M., Wang C., Willner I. (2020). Biocatalytic cascades operating on
macromolecular scaffolds and in confined environments. Nat. Catal..

[12] Wheeldon I., Minteer S. D., Banta S., Barton S. C., Atanassov P., Sigman M. (2016). Substrate channelling
as an approach to cascade reactions. Nat. Chem..

[13] Castellana M., Wilson M. Z., Xu Y., Joshi P., Cristea I. M., Rabinowitz J. D., Gitai Z., Wingreen N. S. (2014). Enzyme clustering
accelerates processing of intermediates through metabolic channeling. Nat. Biotechnol..

[14] Alfano C., Fichou Y., Huber K., Weiss M., Spruijt E., Ebbinghaus S., De Luca G., Morando M. A., Vetri V., Temussi P. A. (2024). Molecular crowding: the history and development
of a scientific paradigm. Chem. Rev..

[15] Ouyang Y., Dong J., Willner I. (2023). Dynamic DNA
Networks-Guided Directional
and Orthogonal Transient Biocatalytic Cascades. J. Am. Chem. Soc..

[16] Zhang Y., Tsitkov S., Hess H. (2016). Proximity
does not contribute to
activity enhancement in the glucose oxidase–horseradish peroxidase
cascade. Nat. Commun..

[17] Linko V., Eerikäinen M., Kostiainen M. A. (2015). A modular DNA origami-based enzyme
cascade nanoreactor. Chem. Commun..

[18] You M., Wang R.-W., Zhang X., Chen Y., Wang K., Peng L., Tan W. (2011). Photon-regulated DNA-enzymatic nanostructures
by molecular assembly. ACS Nano.

[19] Chu G.-B., Li W. Y., Han X. X., Sun H. H., Han Y., Zhi G. Y., Zhang D. H. (2023). Co-Immobilization of GOD & HRP
on Y-Shaped DNA Scaffold and the Regulation of Inter-Enzyme Distance. Small.

[20] Kahn J. S., Xiong Y., Huang J., Gang O. (2022). Cascaded Enzyme
Reactions
over a Three-Dimensional, Wireframe DNA Origami Scaffold. JACS Au.

[21] Zhang Y., Xing C., Mu Z., Niu Z., Feng X., Zhang Y., Wang B. (2023). Harnessing Self-Repairing
and Crystallization
Processes for Effective Enzyme Encapsulation in Covalent Organic Frameworks. J. Am. Chem. Soc..

[22] Chapanian R., Kwan D. H., Constantinescu I., Shaikh F. A., Rossi N. A. A., Withers S. G., Kizhakkedathu J. N. (2014). Enhancement of biological reactions
on cell surfaces via macromolecular crowding. Nat. Commun..

[23] Saini B., Mukherjee T. K. (2023). Biomolecular condensates regulate enzymatic activity
under a crowded milieu: synchronization of liquid–liquid phase
separation and enzymatic transformation. J.
Phys. Chem. B.

[24] Zhang Y., Hess H. (2017). Toward Rational Design
of High-efficiency Enzyme Cascades. ACS Catal..

[25] Lancaster L., Abdallah W., Banta S., Wheeldon I. (2018). Engineering
enzyme
microenvironments for enhanced biocatalysis. Chem. Soc. Rev..

[26] Rivas G., Minton A. P. (2022). Influence of nonspecific
interactions on protein associations:
implications for biochemistry in vivo. Annu.
Rev. Biochem..

[27] Bhakuni K., Bisht M., Venkatesu P., Mondal D. (2019). Designing biological
fluid inspired molecularly crowded ionic liquid media as a sustainable
packaging platform for cytochrome c. Chem. Commun..

[28] Bharadwaj P., Sarkar D. K., Bisht M., Shet S. M., Kotrappanavar N. S., Lokesh V., Franklin G., Brezovsky J., Mondal D. (2023). Nano-structured hydrotrope-caged cytochrome c with
boosted stability in harsh environments: a molecular insight. Green Chem..

[29] Thayallath S. K., Shet S. M., Bisht M., Bharadwaj P., Pereira M. M., Franklin G., Nataraj S., Mondal D. (2023). Designing
protein nano-construct in ionic liquid: a boost in efficacy of cytochrome
C under stresses. Chem. Commun..

[30] Veríssimo N. V., Vicente F. A., de Oliveira R. C., Likozar B., Oliveira R. P. d. S., Pereira J. F. B. (2022). Ionic liquids as protein stabilizers
for biological and biomedical applications: A review. Biotechnol. Adv..

[31] Brogan A. P., Bui-Le L., Hallett J. P. (2018). Non-aqueous
homogenous biocatalytic
conversion of polysaccharides in ionic liquids using chemically modified
glucosidase. Nat. Chem..

[32] Bharmoria P., Tietze A. A., Mondal D., Kang T. S., Kumar A., Freire M. G. (2024). Do ionic liquids exhibit the required
characteristics
to dissolve, extract, stabilize, and purify proteins? Past-present-future
assessment. Chem. Rev..

[33] Hallamaa M., Naapuri J. M., Silva R. A. L., Rosatella A. A., Deska J. (2025). Choline Oxidase and Choline Ionic Liquids in Biocatalytic Heme Peroxidase
Cascades. ChemCatChem.

[34] Bisht M., Mondal D., Pereira M. M., Freire M. G., Venkatesu P., Coutinho J. (2017). Long-term protein packaging in cholinium-based Bio-ionic
liquids: improved catalytic activity and enhanced stability of cytochrome
c against multiple stresses. Green Chem..

[35] Vogt S., Schneider M., Schäfer-Eberwein H., Nöll G. (2014). Determination
of the pH dependent redox potential of glucose oxidase by spectroelectrochemistry. Anal. Chem..

[36] Morales-Urrea D., López-Córdoba A., Contreras E. M. (2023). Inactivation
kinetics of horseradish peroxidase (HRP) by hydrogen peroxide. Sci. Rep..

[37] Bharmoria P., Trivedi T. J., Pabbathi A., Samanta A., Kumar A. (2015). Ionic liquid-induced
all-α to α+β conformational transition in cytochrome
c with improved peroxidase activity in aqueous medium. Phys. Chem. Chem. Phys..

[38] Bharmoria P., Mondal D., Pereira M. M., Neves M. C., Almeida M. R., Gomes M. C., Mano J. F., Bdikin I., Ferreira R. A., Coutinho J. A., Freire M. G. (2020). Instantaneous fibrillation of egg
white proteome with ionic liquid and macromolecular crowding. Commun. Mater..

[39] Sweetlove L. J., Fernie A. R. (2018). The role of dynamic
enzyme assemblies and substrate
channelling in metabolic regulation. Nat. Commun..

[40] Yu H., Dalby P. A. (2018). Exploiting correlated molecular-dynamics networks to
counteract enzyme activity–stability trade-off. Proc. Natl. Acad. Sci. U.S.A..

[41] Eberhardt J., Santos-Martins D., Tillack A. F., Forli S. (2021). AutoDock Vina 1.2.0:
New Docking Methods, Expanded Force Field, and Python Bindings. J. Chem. Inf. Model..

[42] Trott O., Olson A. J. (2010). AutoDock Vina: Improving the speed and accuracy of
docking with a new scoring function, efficient optimization, and multithreading. J. Comput. Chem..

[43] Mogharrab N., Ghourchian H., Amininasab M. (2007). Structural Stabilization and Functional
Improvement of Horseradish Peroxidase upon Modification of Accessible
Lysines: Experiments and Simulation. Biophys.
J..

[44] Bharadwaj P., Barua A., Bisht M., Sarkar D. K., Biswas S., Franklin G., Mondal D. (2024). Understanding
the Effect of Ionic
Liquid–Mediated Solvent Engineering on the Kinetics and Thermodynamic
Stability of Phenylalanine Ammonia-Lyase. J.
Phys. Chem. B.

[45] Sanchez-Fernandez A., Basic M., Xiang J., Prevost S., Jackson A. J., Dicko C. (2022). Hydration in Deep Eutectic
Solvents Induces Non-monotonic Changes
in the Conformation and Stability of Proteins. J. Am. Chem. Soc..

[46] Ragone R., Colonna G., Balestrieri C., Servillo L., Irace G. (1984). Determination
of tyrosine exposure in proteins by second-derivative spectroscopy. Biochemistry.

[47] Greenfield N. J. (2006). Using circular
dichroism collected as a function of temperature to determine the
thermodynamics of protein unfolding and binding interactions. Nat. Protoc..

[48] Wolfenden R., Snider M. J. (2001). The Depth of Chemical
Time and the Power of Enzymes
as Catalysts. Acc. Chem. Res..

[49] Kari J., Molina G. A., Schaller K. S., Schiano-di-Cola C., Christensen S. J., Badino S. F., Sørensen T. H., Røjel N. S., Keller M. B., Sørensen N. R. (2021). Physical constraints and functional plasticity of cellulases. Nat. Commun..

[50] Warshel A. (1998). Electrostatic
origin of the catalytic power of enzymes and the role of preorganized
active sites. J. Biol. Chem..

[51] Sousa S. F., Calixto A. R., Ferreira P., Ramos M. J., Lim C., Fernandes P. A. (2020). Activation Free Energy, Substrate Binding Free Energy,
and Enzyme Efficiency Fall in a Very Narrow Range of Values for Most
Enzymes. ACS Catal..

[52] Abramson J. (2024). Accurate structure prediction of biomolecular
interactions with AlphaFold
3. Nature.

[53] Yan Y. (2020). The HDOCK server for
integrated protein–protein docking. Nat.
Protoc..

[54] Singh, A. ; GRAMM web server for protein docking. In Computational Drug Discovery and Design; Springer US: New York, NY, 2023; pp 101–112. 10.1007/978-1-0716-3441-7_5 37676594

