# Supplementary material for: Stoichiometrically Engineered Hydrated Ionic Liquids Enabling Reinforcement of Enzyme Cascade with Improved Thermodynamic Stability
Source: ACS Sustain Chem Eng. 2026 Mar 16;14(12):6115–28. doi: 10.1021/acssuschemeng.5c13384 (PMC13041476; doi:10.1021/acssuschemeng.5c13384)
Supplement: Supplementary file 1 [file sc5c13384_si_001.pdf]

Electronic Supplementary Information (ESI)

Stoichiometrically Engineered Hydrated Ionic Liquids Enabling Reinforcement of Enzyme Cascades with Improved Thermodynamic Stability

Sagar Biswas,[1] Dheeraj Kumar Sarkar,[2] Aaftaab Sethi,[1,3] Pranav Bharadwaj,[1] Rakesh Sinha,[1] Pankaj Bharmoria,[4,5] Gregory Franklin,[1] Dibyendu Mondal*[1,6]

[1]*Institute of Plant Genetics, Polish Academy of Sciences, Strzeszyńska 34, 60-479 Poznań, Poland.*

[2]*Tata Institute of Fundamental Research, Hyderabad, Telangana, India-500046.*

[3]*Laboratory of Biomolecular Interactions and Transport, Department of Gene Expression, Institute of Molecular Biology and Biotechnology, Faculty of Biology, Adam Mickiewicz University, Uniwersytetu Poznanskiego 6, Poznan 61-614, Poland.*

[4]*Institute of Materials Science of 765 Barcelona, ICMAB-CSIC, Barcelona 08193, Spain*

[5] *Department of Chemical Engineering, Universitat Politècnica de Catalunya, EEBE, Barcelona 08019, Spain*

[6]*Centre for Nano and Material Sciences, Jain (Deemed-to-be University), Jain Global Campus, Kanakapura, Bangalore, Karnataka 562112, India*

**Corresponding author, DM: dmon@igr.poznan.pl; m.dibyendu@jainuniversity.ac.in*

Table of Contents

Sr. No.	Title	Page No.
1.	Materials and Methods	S3
2.	^{1}H NMR of ILs (Figure S1-S8)	S3-S7
3.	Table S1: pH of ILs at concentrations in DI water	S7
3.	Figure S9: HRP catalyzed reaction monitored by the change in absorption of ABTS at 420 nm.	S11
4.	Figure S10: GOx catalyzed reaction monitored by the change in absorption of ABTS at 420 nm.	S12
5.	Figure S11: GOx-HRP cascade reaction monitored by the change in absorption of ABTS at 420 nm.	S12
6.	Table S2-S10: Docked scores of $[Ch]^{+1}$, $[Dhp]^{-1}$, $[Dhp]^{-2}$, $[Dhp]^{-3}$, $[Mal]^{-1}$, $[Mal]^{-2}$, $[PAA]^{-1}$, $[PAA]^{-2}$ and $[PAA]^{-3}$ for HRP and GOx	S13-S15
7.	Figure S12-S20: All docking configurations of $[Ch]^{+1}$, $[PAA]^{-1}$, $[PAA]^{-2}$, $[PAA]^{-3}$, $[Mal]^{-1}$, $[Mal]^{-2}$, $[Dhp]^{-1}$, $[Dhp]^{-2}$ and $[Dhp]^{-3}$ at different putative binding sites of HRP and GOx	S16-S20
8.	Figure S21: RMSF and RMSD plots of GOx and HRP in presence of 2:1 IL composition	S20
9.	Figure S22: Second derivative UV-Vis spectra of HRP and GOx in all ILs (10 wt%) including PBS (7.4)	S21
10.	Figure S23: UV-Vis spectra of HRP and GOx in all ILs (10 wt%) including PBS (7.4)	S21
11.	Figure S24: Analysis of CD spectra of HRP and GOx in all synthesized ILs (10 wt%) including PBS (7.4)	S22
12.	Figure S25: Reduced and native gel electrophoresis of HRP and GOx in different ILs	S22
13.	Figure S26: Kinetic plots of HRP catalyzed reaction	S23
14.	Table S11-S14: Kinetic parameters of HRP catalyzed reaction	S24
15.	Figure S27: Kinetic plots of GOx catalyzed reaction	S25
16.	Table S15-S18: Kinetic parameters of GOx catalyzed reaction	S26
17.	Figure S28: Kinetic plots of GOx-HRP Cascade reaction	S27
18.	Table S19-22: Kinetic parameters of GOx-HRP Cascade reaction	S28
19.	Figure S29: Analysis of Thermal properties of GOx and HRP in ILs using CD spectra	S29
20.	Table S23: Comparison of GOx-HRP kinetic and thermal stability from the current work with the literature	S30
21.	Table S24-25	S31
22.	References	S31- S33

1. Materials and methods

Peroxidase from *Horseradish (HRP)* (CAS: 9003-99-0), Glucose Oxidase (GOx) from *Aspergillus niger* (CAS: 9001-37-0), choline bicarbonate (80 wt% in water), phosphoric acid, malonic acid and phosphonoacetic acid, hydrogen peroxide, 2-Mercaptoethanol, 2,2’-Azino-bis(3-ethylbenzothiazoline-6-sulfonic acid) diammonium salt (ABTS), sodium phosphate monobasic monohydrate and sodium phosphate dibasic were purchased from Merck. PageRuler™ pre-stained protein ladder, 10 to 180 kDa (Catalog number: 26616), 2x Laemmli sample buffer for protein gels (Catalogue no: #1610737); native sample buffer for protein gels, 30 mL(Catalogue no: #1610738); 10x Tris/Glycine/SDS (cat: #1610732 pkg of 1, 1 L, 10x premixed electrophoresis buffer, contains 25 mM Tris, 192 mM glycine, 0.1% SDS, pH 8.3 following dilution to 1x with water), Mini-Protein TGX Gels 12%, 10 well comb were obtained from Bio-Rad. Ultrapure Milli-Q water was used for preparing enzyme (HRP and GOx) and ILs solutions to observe the catalytic activity.

2. Synthesis of pH-switchable cholinium-based Ionic Liquids (ILs)

Cholinium-based ILs were synthesized following a standard protocol through a simple acid-base reaction.[1] Different molar ratios (1:1, 2:1, and 3:1) of aqueous choline bicarbonate and the corresponding phosphoric acid, malonic acid, and phosphonoacetic acid acids were slowly added in portions with continuous stirring at 75 °C. The reaction mixture was kept stirring for 12 h. The ILs were dried in rotavapor and ILs with 3±1 wt% of water were then collected and further used to study the enzymatic reaction. Purity of the ILs was confirmed by ^{1}H-NMR spectroscopy. The chemical structure and composition of the ILs employed for the current study are shown in Figure 1A.

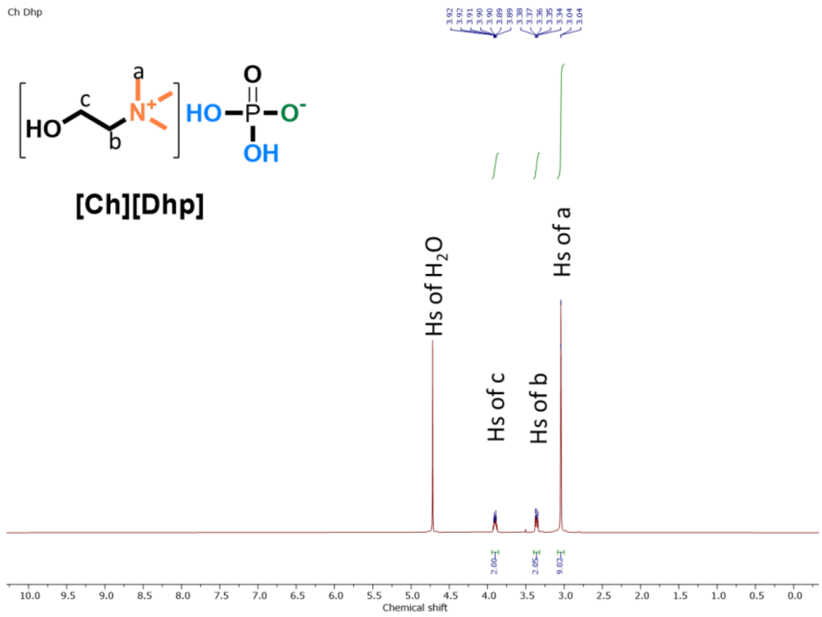

Figure S1: ^{1}H NMR spectra of [Ch][Dhp] in D_2O.

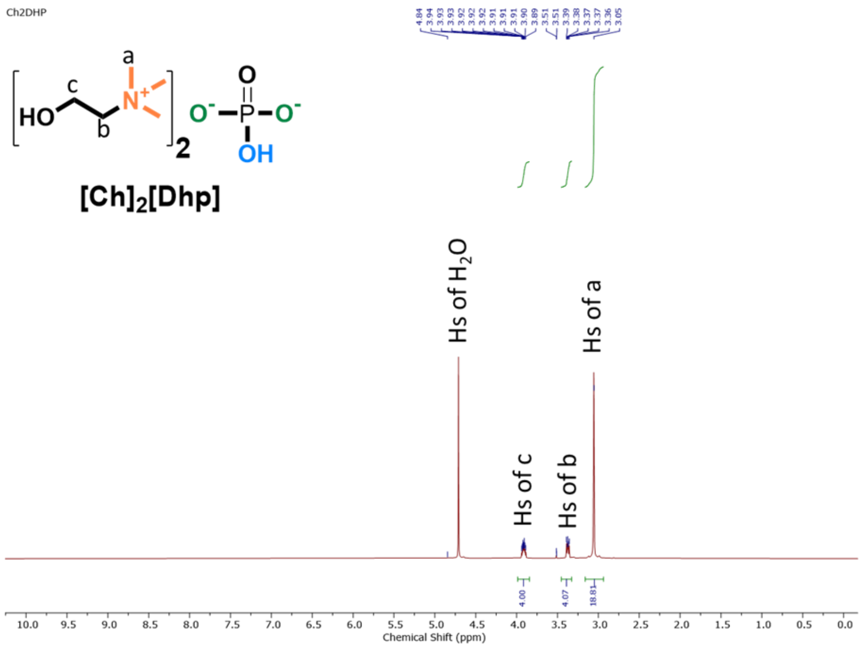

Figure S2: ^{1}H NMR spectra of $[Ch]_2[Dhp]$ in D_2O.

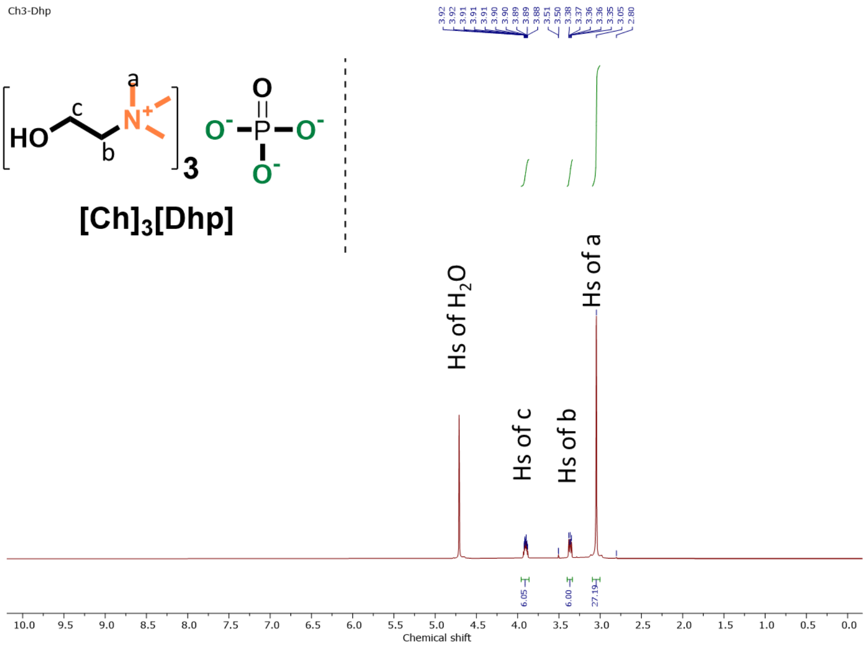

Figure S3: ^{1}H NMR spectra of $[Ch]_3[Dhp]$ in D_2O.

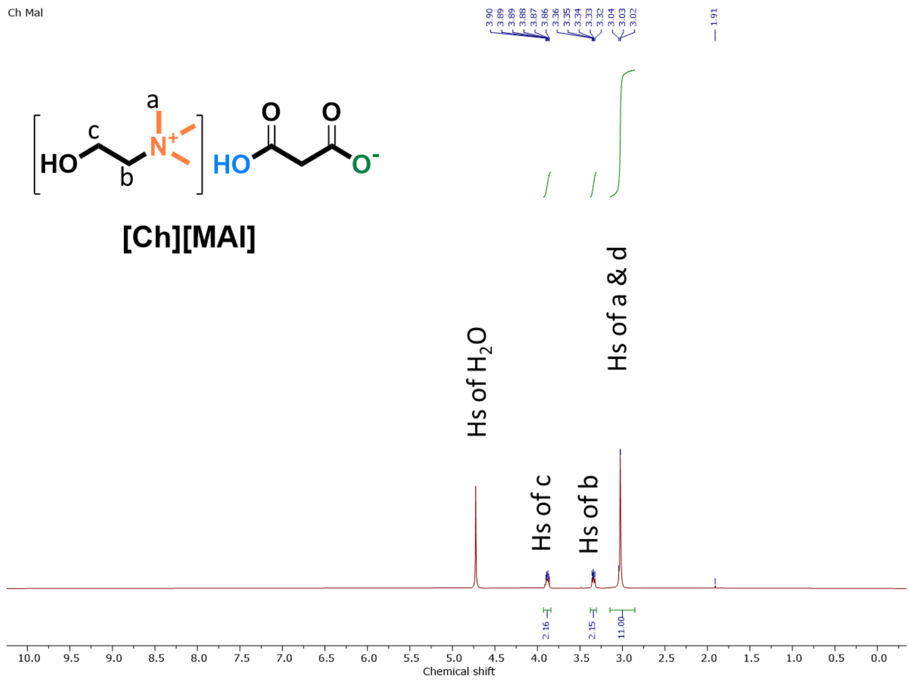

Figure S4: ^{1}H NMR spectra of [Ch][Mal] in D_2O.

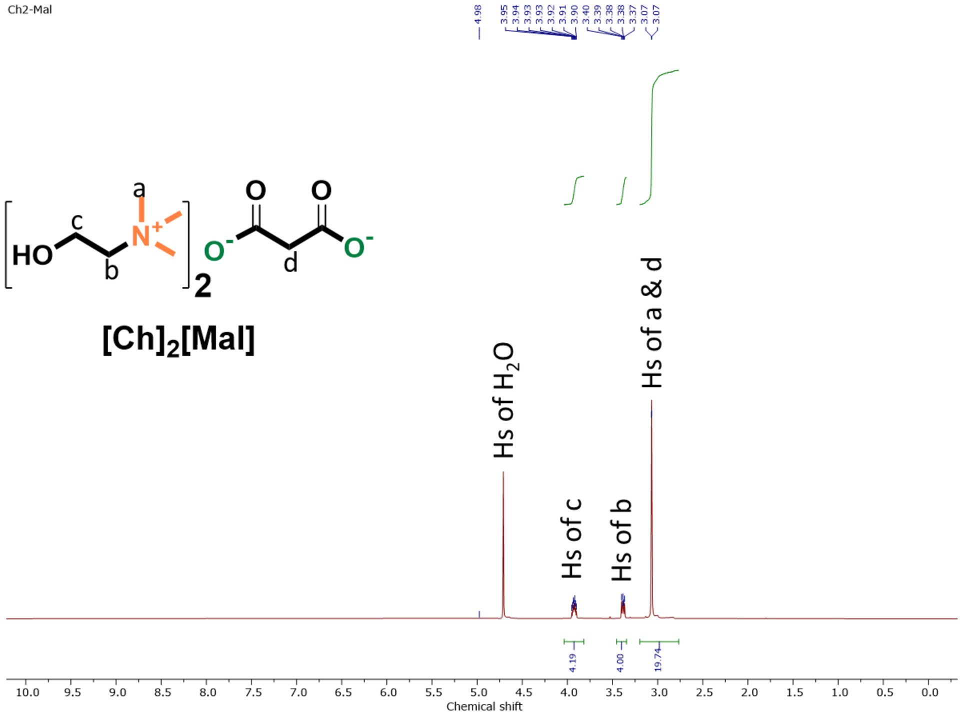

Figure S5: ^{1}H NMR spectra of $[Ch]_2$[Mal] in D_2O.

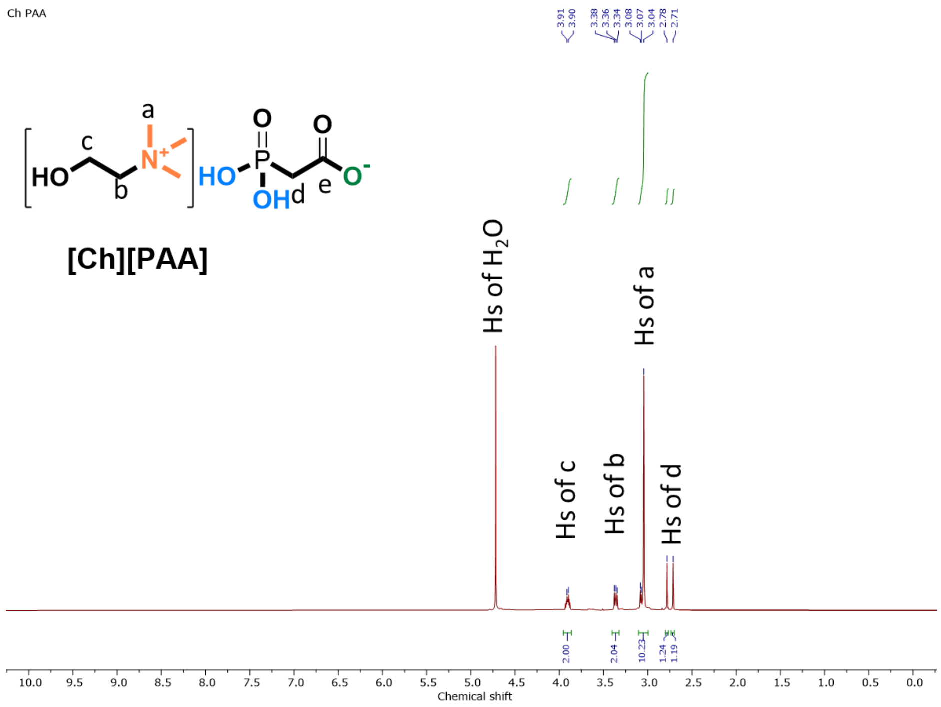

Figure S6: ^{1}H NMR spectra of [Ch][PAA] in D_2O.

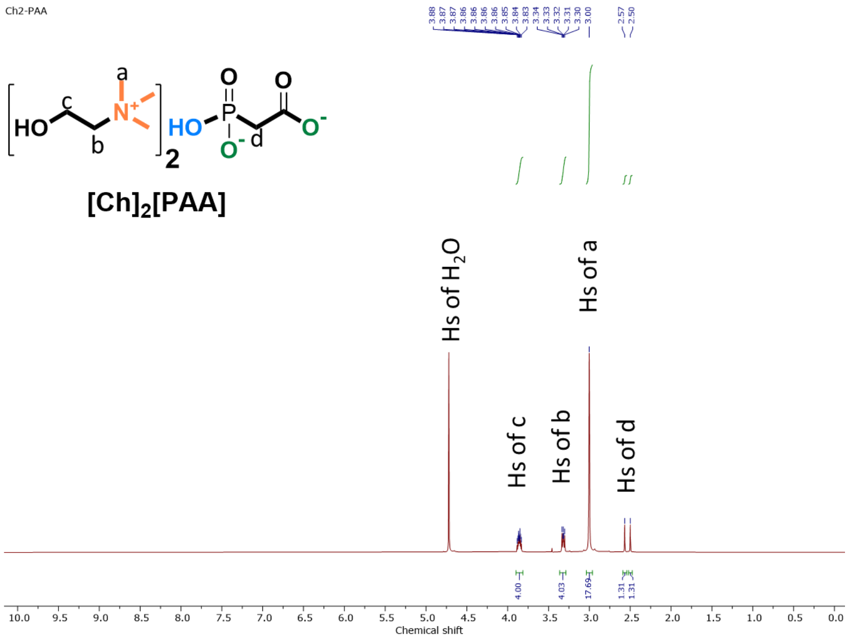

Figure S7: ^{1}H NMR spectra of $[Ch]_2[PAA]$ in D_2O.

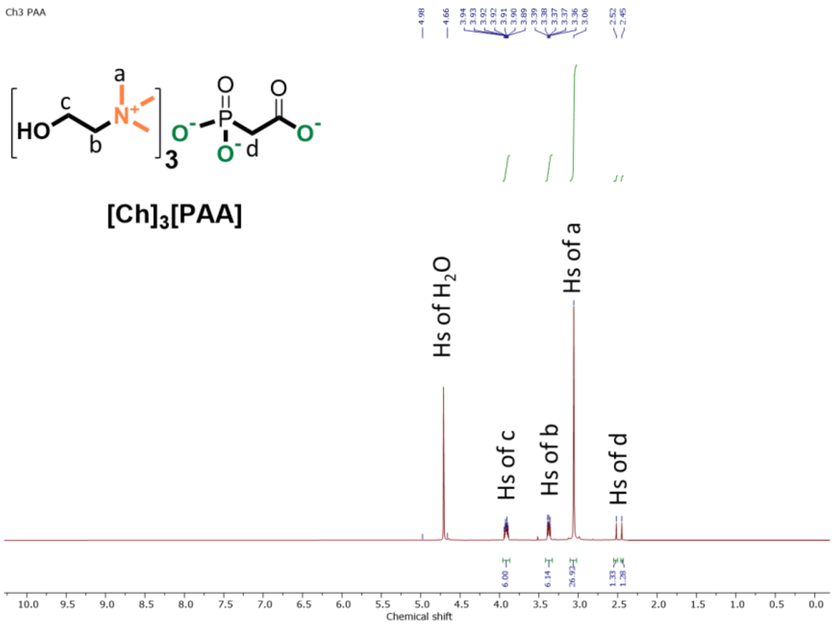

Figure S8: ^{1}H NMR spectra of $[Ch]_3[PAA]$ in D_2O.

3. Measurement pH of the ILs

The pH of all the ILs solutions was measured using Eutech pH 700 pH meter having the working range of 2 to 16 with an accuracy of ±0.01 pH. The ILs solution from 10 wt% to 50 wt% was prepared by taking the synthesized ILs into milli-Q water. Then, all the solutions were individually used to observe the pH of the solution and were tabulated in supplementary Table S1. All the experiments were replicated thrice.

Table S1: pHs of ILs at different percentages diluted in DI water

wt. (%) of ILs	[Ch][DHP]	$[Ch]_2$[DHP]	$[Ch]_3$[DHP]	[Ch][Mal]	$[Ch]_2$[Mal]	[Ch][PAA]	$[Ch]_2$[PAA]	$[Ch]_3$[PAA]
50	3.46 ± 0.015	7.72 ± 0.02	8.14 ± 0.015	3.51 ± 0.02	5.85 ± 0.189	2.59 ± 0.01	5.26 ± 0.03	7.97 ± 0.025
40	3.28 ± 0.01	7.41 ± 0.05	7.98 ± 0.01	3.35 ± 0.05	5.60 ± 0.092	2.54 ± 0.015	5.11 ± 0.021	7.77 ± 0.02
30	3.12 ± 0.017	7.25 ± 0.012	7.85 ± 0.005	3.25 ± 0.017	5.42 ± 0.03	2.46 ± 0.021	4.96 ± 0.021	7.62 ± 0.021
20	2.96 ± 0.01	7.12 ± 0.015	7.79 ± 0.006	3.15 ± 0.025	5.28 ± 0.023	2.34 ± 0.03	4.90 ± 0.03	7.51 ± 0.02
10	2.82 ± 0.02	7.01 ± 0.012	7.74 ± 0.01	3.08 ± 0.015	5.20 ± 0.055	2.30 ± 0.021	4.82 ± 0.015	7.45 0.023

4. Enzymatic assays preparation

Quantifications of all the enzymes were carried out on a Shimadzu UV-1900i spectrophotometer with a quartz cuvette of 1 cm path length. The extinction coefficients used to calculate the concentration of GOx at 280 and HRP at 403 nm are 2.67×10^5 M^{-1} cm^{-1}, 1.0×10^5 M^{-1} cm^{-1}, respectively.[2] In each case, the enzymatic activities were calculated by observing the changes in absorbance at 420 nm using the spectrophotometer considering the final product $ABTS^{+\cdot}$ (ε_{420} nm=3.6×10^4 M^{-1} cm^{-1}).[2]

4.1 HRP activity assay

HRP activity assay was developed using 10 µL of 100 nM HRP, 10 µL of 10 mM H_2O_2 and 50 µL of 40 mM 2,2′-azino-bis(3-ethylbenzothiazoline-6-sulponic acid)-diammonium salt (ABTS) solution was added to 930 µL of PBS buffer (100 mM, pH 7.4) and to all the ILs solution (10 wt% to 50 wt%). The assay solution was incubated for 5 minutes before adding H_2O_2, and the reaction progress was monitored using UV-Vis spectroscopy. The absorbance at 420 nm was observed for the first 3 min and the activity was calculated from the slope plotting the absorbance at 420 nm against time with the first 1 min duration (Figure S1). Relative activity of HRP was calculated from the following formula.[2]

$$\% \, Relative\ activity = \frac{(Slope\ of\ HRP\ catalysed\ reaction\ in\ ILs\ \times\ 100)}{(Slope\ of\ HRP\ catalysed\ reaction\ in\ buffer)}$$

4.2 GOx activity assay

GOx kinetic assay was prepared using 10 µL of 100 nM GOx, 20 µLof 1 µM HRP, 50 µL of 40 mM 2,2′-azino-bis(3-ethylbenzothiazoline-6-sulponic acid)-diammonium salt (ABTS) and 50 µL of substrate solution was added to 870 µL (PBS pH 7.4 and all the ILs with different pH with the concentration of 10mM to 100 mM) to initiate the reaction. The final assay solution contains 2 mM ABTS, 100 mM glucose, 1 nM GOx and 20 nM HRP. The increase in absorbance at 420 nm was recorded. The activity was calculated from the slope which was obtained after plotting absorbance at 420 nm against time with duration of the first 1 min (Figure S2). Relative activity of GOx was calculated from the following formula.[2]

$$\% \, Relative\ activity = \frac{(Slope\ of\ GOx\ catalysed\ reaction\ in\ ILs\ \times\ 100)}{(Slope\ of\ GOx\ catalysed\ reaction\ in\ buffer)}$$

5. Second derivative of UV-Visible spectra

Structural aspect of HRP and GOx in different ILs (10 wt%) as well as in PBS (pH 7.4) was elucidated using Shimadzu UV-1900i spectrophotometer with a quartz cuvette of 1 cm path length with an observed range of 200 nm to 800 nm. All the spectra were recorded using quartz cuvette of 1 cm path length. The enzyme concentration was fixed to 0.125 mg/mL for all the samples. The UV-Vis spectra were converted mathematically to 2nd derivative using OriginPro 2023 software. The structural changes of both HRP and GOx were analyzed for both tyrosine and tryptophan residues within the range of around 280-285 and 290-295nm respectively.[3] For precise understanding the ratio of difference between their individual peaks were calculated and inferred mathematically using the second derivative as follows.

$$\frac{d^2A_{Tyr}}{d^2A_{Trp}} = \frac{(Tyr_{\ min} - Tyr_{\ max})}{(Trp_{\ min} - Trp_{\ max})}$$

Tyr_{min} and Tyr_{max} represent the lowest and highest peaks of negative and positive $d^2A/d\lambda^2$ values, respectively, within the 280-285 nm range. Similarly, Trp_{min} and Trp_{max} denote the lowest and highest peaks of negative and positive $d^2A/d\lambda^2$ values, respectively, within the 290-295 nm range.

6. Native and SDS-PAGE of HRP and GOx in the presence of ILs

The stability of GOx and HRP in PBS and various ILs was evaluated using SDS-PAGE and native PAGE. The ILs studied included 1:1 and 2:1 cholinium-based ILs: [Ch][Dhp], [Ch][Mal], [Ch][PAA], $[Ch]_2[Dhp]$, $[Ch]_2[Mal]$, and $[Ch]_2[PAA]$.

For SDS-PAGE analysis, GOx and HRP were individually dissolved in 10 wt% solutions of each IL. Each enzyme-IL mixture was then combined with 2× Laemmli sample buffer containing β-mercaptoethanol (prepared by adding 50 µL β-mercaptoethanol to 950 µL of 2× Laemmli buffer). Prior to loading onto the gel, the samples were heated at 90 °C for 5 minutes to ensure complete denaturation.[4]

For native PAGE, GOx or HRP was mixed with 2× native sample buffer in a 1:2 volume ratio (enzyme:buffer), without heating.

For both SDS-PAGE and native PAGE, 30 µL of each prepared sample—containing 5 µg of enzyme—was loaded into the wells of a 12% precast polyacrylamide gel (Mini-Protein TGX). Electrophoresis was carried out at 100 V for 4 hours using 10× Tris-Glycine/SDS running buffer. After electrophoresis, the gels were stained with silver nitrate for 20 seconds. Following destaining, protein bands were visualized and analyzed by comparison with a pre-stained protein ladder (PageRuler).

7. Thermodynamic stability of HRP and GOx

The thermodynamic stability of HRP and GOx were assessed using circular dichroism (CD) spectroscopy for which a Jasco-1500 spectrophotometer equipped with Peltier system for temperature control was used in presence of all ILs (1 wt%) as well as in PBS. The CD spectra were recorded using quartz cuvettes of path length 1 cm with the chosen concentration of 0.125 mg/mL for both HRP and GOx solutions. For the thermal stability, a temperature range of 25 °C to 90 °C was chosen with a heating rate of 2 °C min^{-1}, nitrogen flow rate of 4 L min^{-1}, data interval of 5 °C, and 100 nm min^{-1} scan speed. Each CD spectrum was corrected by subtracting the corresponding reference samples and accumulated in triplicates.

Further, a two-state model was considered to determine the melting temperature (T_m), unfolding pattern and the corresponding fraction of native state (f_N) and unfolded state (f_U) was evaluated using the following relation.

$$\text{Protein (folded)} \rightleftharpoons \text{Protein (unfolded)}$$

$$f_N = \frac{(\theta-\theta_0)}{(\theta_{max}-\theta_0)} \quad \& \quad f_U = 1 - f_N$$

Where, θ is CD signal of corresponding temperature at 220 nm; θ_0 is the signal of minimum intensity correlating to the denatured state and θ_{max} is the signal with maximum magnitude (in the negative axis) indicating native state of HRP or GOx.[5]

The melting temperature (T_m) of both HRP and GOx for a given system was derived mathematically using the derivative of sigmoidal curve that was obtained from the plot of $f_{Unfolded}$ vs temperature (T).

Gibbs free energy of unfolding was calculated from experimentally obtained equilibrium constant K at all the temperature as follows.

$$K = \frac{f_U}{f_N} = \frac{1 - f_N}{f_N}$$

$$\Delta G = -RT\ln K$$

Where R is the universal gas constant = 8.314 J $K^{-1}mol^{-1}$

8. System setup and Molecular docking

The crystal structures of HRP from *Armoracia rusticana* origin (PDB ID: 1HCH) and GOx from *Aspergillus niger* (PDB ID: 1CF3) were retrieved from PDB database. The three ionic liquids pairs viz. [Ch][Dhp], [Ch][Mal] and [Ch][PAA] were individually docked at HRP and GOx for generating the binding affinities at putative binding sites. The docking was performed for three protonation states of HRP and GOx at pH 2.3, 5 and 7 to understand the interaction preferences for specific IL. Blind dockings were performed for all 18 enzyme-ligand complexes using the Autodock Vina program.[6,7] The electrostatics potential surface was calculated using the Adaptive Poisson-Boltzmann Solver (APBS) method from PyMOL[8] and the charges were converted to millivolt from the relation:

$$V = \left(\frac{k \cdot T}{e}\right) \times 1000 \times X \quad (1)$$

V is the electrostatic potential in millivolts (*mV*), k = Boltzmann constant, T is the temperature in Kelvin, e = elementary charge, X is the charge value in kT/e obtained from PyMol.

9. Molecular dynamics simulations and trajectory analysis

The pKa values of titratable residues and electrostatics for HRP and GOx at pH 2.3, 5, and 7 were calculated using the PropKa server with default settings. Six molecular systems (HRP and GOx at a 2:1 ratio of ILs) were prepared using $[Ch]_2[Mal]$ at pH 5, $[Ch]_2[PAA]$ at pH 5, and $[Ch]_2[Dhp]$ at pH 7 for molecular dynamics simulations. ILs were set to 10 wt% concentration. Simulation boxes of 10 nm in each xyz - axis directions were defined, and the systems were solvated with TIP3P water using the CHARMM-GUI webserver.[9] The IL structures were obtained from PubChem (IDs: 305 [Ch], 1003 [Dhp], 867 [Mal], 546 [PAA]) and protonation states were modelled using PyMOL.[8] Charmm36 force field parameters for ILs were derived using GAAMP.[10] After initial energy minimization via steepest descents, the system was equilibrated for 10 ns in an NVT ensemble, followed by 20 ns in an NPT ensemble with position restraints on protein, lipid, and ligand heavy atoms. A Verlet cutoff scheme (1.2 nm) was used for Lennard-Jones[11] and short-range electrostatic interactions, with long-range interactions treated by PME.[12] Hydrogen bonds were constrained using LINCS,[13] while TIP3P water bonds and angles were constrained with SETTLE.[14] A 2 fs time step was used throughout. Temperature and pressure were maintained at 300 K and 1 bar during equilibration with Berendsen thermostat and barostat (1 ps and 5 ps time constants). Production runs used the Nose-Hoover thermostat and Parrinello-Rahman barostat. All simulations were performed for 1 µs time for each molecular systems and with given ratio of ILs. After the MD simulations, root mean square deviation (RMSD) and root mean square fluctuations (RMSF) were derived using MDAnalysis. To quantify structural differences between frames within each trajectory, a pairwise root mean square deviation (RMSD) matrix was computed. For each pair of frames i and j, the protein atoms were aligned, and the RMSD was calculated as the square root of the mean squared distance between corresponding atom positions (Equation 6):

$$RMSD_{i,j} = \sqrt{\frac{1}{N}\sum_{k=1}^{N}\left\|r_i^k - r_j^k\right\|^2} \quad (6)$$

where N is the number of protein atoms, and r_i^k and r_j^k are the coordinates of atom k in frames i and j, respectively. The RMSD values were stored in a symmetric n × n matrix, where n is the total number of frames in the trajectory. The DBSCAN algorithm,[15] implemented in the scikit-learn library,[16] was used with default parameters to cluster frames based on the precomputed RMSD matrix. Finally, structurally similar groups of frames were generated.

10. Computational methods for the study of GOx-HRP complex interaction with ILs

The crystal structures of HRP (PDB ID: 1HCH) and GOx (PDB ID: 1CF3) were retrieved from the Protein Data Bank. All crystallographic water molecules and heteroatoms were removed prior to complex modeling. Protein sequences of HRP (UniProt ID: P00433) and GOx (UniProt ID: P13006) were also submitted to the AlphaFold server to obtain predictions of the complex.[17]

Complex formation between HRP and GOx was evaluated using two independent docking platforms: HDOCK,[18] and GRAMM.[19] In HDOCK, the hybrid approach combining template-based and free docking was employed with default parameters. In GRAMM, both template-based and free docking strategies were applied to validate the binding orientation.

The top-scoring GOx-HRP complex derived was further prepared for IL docking. Protonation states at three different pH values (4.80, 5.20 and 7.01) were generated using the H++ server,[20] and the resulting complexes were converted into pdbqt format with appropriate charge assignments using MGL tools.[21]

Ionic liquids (ILs) were prepared from their SMILES strings, converted to pdbqt format using OpenBabel,[22] and subsequently minimized using the MMFF94 force field. Docking of ILs was performed using AutoDock Vina with the following grid parameters: size_x = 80, size_y = 80, size_z = 80, grid center at x = 5.896, y = 2.373, z = 1.983, exhaustiveness set to 32, maximum output modes = 10 and spacing = 0.40 Å.[6-7] Docking was carried out for the GOx-HRP receptor for each of the three protonation states.

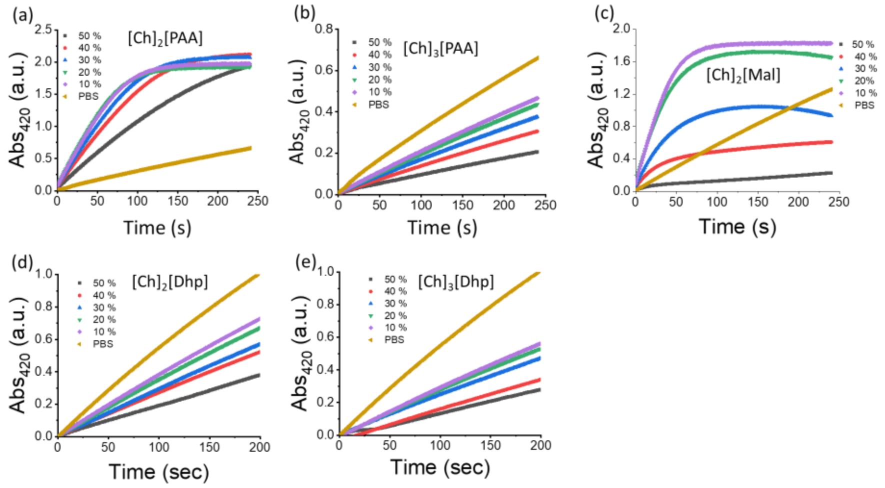

Figure S9: HRP catalyzed reaction monitored by the change in absorption of ABTS at 420 nm. Real time kinetics plot of Absorbance at 420 nm vs time up to 240 sec is shown in figure (a), (b) for $[Ch]_2[PAA]$, $[Ch]_3[PAA]$; (c) for $[Ch]_2[Mal]$; and (d), (e) for $[Ch]_2[Dhp]$, $[Ch]_3[Dhp]$ respectively. In all the cases HRP and H_2O_2 concentration was fixed at 1 nM and 100 µM.

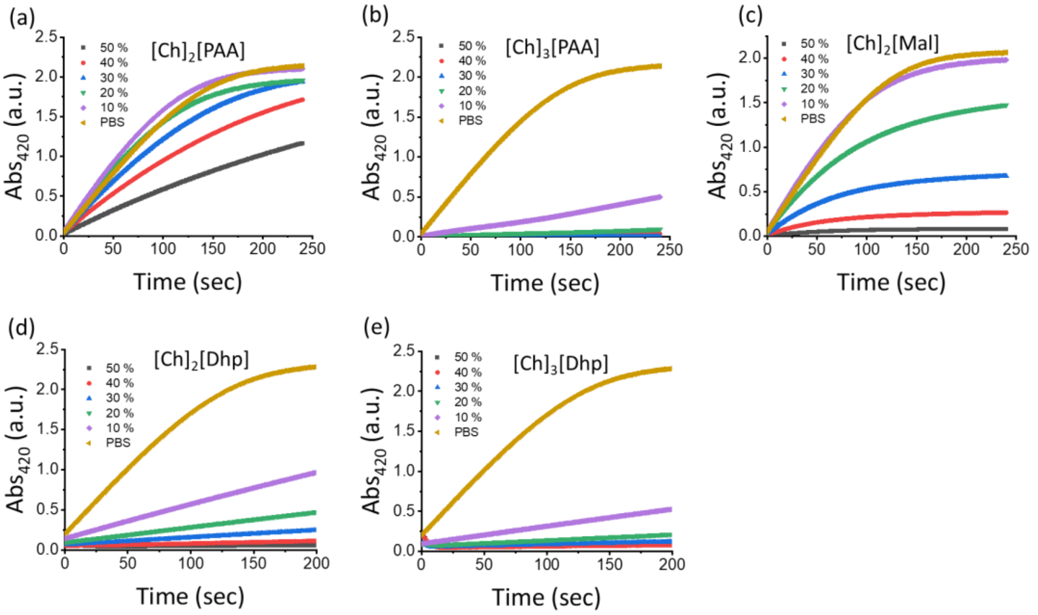

Figure S10: GOx catalyzed reaction monitored by the change in absorption of ABTS at 420 nm. Real time kinetics plot of Absorbance at 420 nm vs time is shown in figure (a), (b) for $[Ch]_2[PAA]$, $[Ch]_3[PAA]$; (c) for $[Ch]_2[Mal]$; and (d), (e) for $[Ch]_2[Dhp]$, $[Ch]_3[Dhp]$ respectively. In all the cases HRP and GOx concentration was fixed at 20 nM and 1 nM, respectively.

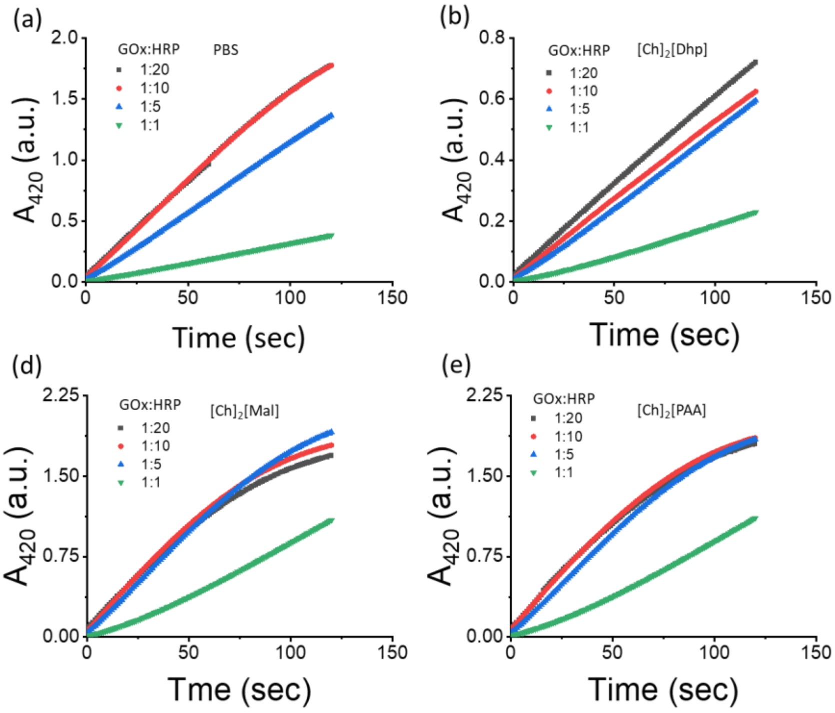

Figure S11: GOx-HRP cascade reaction monitored by the change in absorption of ABTS at 420 nm. (a), (b), (c) and (d) shows cascade activity in PBS, $[Ch]_2[Dhp]$, $[Ch]_2[Mal]$ and $[Ch]_2[PAA]$. In all the cases ILs concentration was fixed to 10 wt%. For both GOx concentration was fixed at 1 nM and HRP concentration was varied from 1-20 nM.

Table S2. Docked scores of $[Ch]^{+1}$ for GOx and HRP.

GOx				HRP			
Rank	Name	Pose	SCORE	Rank	Name	Pose	Score
1	$[Ch]^{+1}$	1	-3.8	1	$[Ch]^{+1}$	1	-3.7
2	$[Ch]^{+1}$	2	-3.7	2	$[Ch]^{+1}$	2	-3.6
3	$[Ch]^{+1}$	3	-3.7	3	$[Ch]^{+1}$	3	-3.6
4	$[Ch]^{+1}$	4	-3.7	4	$[Ch]^{+1}$	4	-3.5
5	$[Ch]^{+1}$	5	-3.5	5	$[Ch]^{+1}$	5	-3.5
6	$[Ch]^{+1}$	6	-3.5	6	$[Ch]^{+1}$	6	-3.4
7	$[Ch]^{+1}$	7	-3.5	7	$[Ch]^{+1}$	7	-3.3
8	$[Ch]^{+1}$	8	-3.4	8	$[Ch]^{+1}$	8	-3.3
9	$[Ch]^{+1}$	9	-3.4	9	$[Ch]^{+1}$	9	-3.2
10	$[Ch]^{+1}$	10	-3.4	10	$[Ch]^{+1}$	10	-3.2

Table S3. Docked scores of $[Dhp]^{-1}$ for GOx and HRP.

GOx				HRP			
Rank	Name	Pose	Score	Rank	Name	Pose	Score
1	$[Dhp]^{-1}$	1	-4.4	1	$[Dhp]^{-1}$	1	-4.3
2	$[Dhp]^{-1}$	2	-4.4	2	$[Dhp]^{-1}$	2	-4.3
3	$[Dhp]^{-1}$	3	-4.2	3	$[Dhp]^{-1}$	3	-3.9
4	$[Dhp]^{-1}$	4	-4.2	4	$[Dhp]^{-1}$	4	-3.9
5	$[Dhp]^{-1}$	5	-4.1	5	$[Dhp]^{-1}$	5	-3.9
6	$[Dhp]^{-1}$	6	-4.1	6	$[Dhp]^{-1}$	6	-3.7
7	$[Dhp]^{-1}$	7	-4.0	7	$[Dhp]^{-1}$	7	-3.7
8	$[Dhp]^{-1}$	8	-3.9	8	$[Dhp]^{-1}$	8	-3.4
9	$[Dhp]^{-1}$	9	-3.9	9	$[Dhp]^{-1}$	9	-3.3
10	$[Dhp]^{-1}$	10	-3.8	10	$[Dhp]^{-1}$	10	-3.2

Table S4. Docked scores of $[Dhp]^{-2}$ for GOx and HRP.

GOx				HRP			
Rank	Name	Pose	Score	Rank	Name	Pose	Score
1	$[Dhp]^{-2}$	1	-4.1	1	$[Dhp]^{-2}$	1	-4.0
2	$[Dhp]^{-2}$	2	-4.1	2	$[Dhp]^{-2}$	2	-4.0
3	$[Dhp]^{-2}$	3	-4.0	3	$[Dhp]^{-2}$	3	-3.9
4	$[Dhp]^{-2}$	4	-4.0	4	$[Dhp]^{-2}$	4	-3.4
5	$[Dhp]^{-2}$	5	-4.0	5	$[Dhp]^{-2}$	5	-3.4
6	$[Dhp]^{-2}$	6	-3.7	6	$[Dhp]^{-2}$	6	-3.3
7	$[Dhp]^{-2}$	7	-3.7	7	$[Dhp]^{-2}$	7	-3.2
8	$[Dhp]^{-2}$	8	-3.7	8	$[Dhp]^{-2}$	8	-3.2
9	$[Dhp]^{-2}$	9	-3.7	9	$[Dhp]^{-2}$	9	-3.1
10	$[Dhp]^{-2}$	10	-3.5	10	$[Dhp]^{-2}$	10	-3.1

Table S5. Docked scores of $[Dhp]^{-3}$ for GOx and HRP.

GOx				HRP			
Rank	Name	Pose	Score	Rank	Name	Pose	Score
1	$[Dhp]^{-3}$	1	-4.1	1	$[Dhp]^{-3}$	1	-4.0
2	$[Dhp]^{-3}$	2	-4.1	2	$[Dhp]^{-3}$	2	-4.0
3	$[Dhp]^{-3}$	3	-4.0	3	$[Dhp]^{-3}$	3	-3.9
4	$[Dhp]^{-3}$	4	-4.0	4	$[Dhp]^{-3}$	4	-3.9
5	$[Dhp]^{-3}$	5	-4.0	5	$[Dhp]^{-3}$	5	-3.9
6	$[Dhp]^{-3}$	6	-3.7	6	$[Dhp]^{-3}$	6	-3.4
7	$[Dhp]^{-3}$	7	-3.7	7	$[Dhp]^{-3}$	7	-3.2
8	$[Dhp]^{-3}$	8	-3.7	8	$[Dhp]^{-3}$	8	-3.2
9	$[Dhp]^{-3}$	9	-3.7	9	$[Dhp]^{-3}$	9	-3.2
10	$[Dhp]^{-3}$	10	-3.5	10	$[Dhp]^{-3}$	10	-3.2

Table S6. Docked scores of $[Mal]^{-1}$ for GOx and HRP.

GOx				HRP			
Rank	Name	Pose	Score	Rank	Name	Pose	Score
1	$[Mal]^{-1}$	1	-4.7	1	$[Mal]^{-1}$	1	-4.8
2	$[Mal]^{-1}$	2	-4.7	2	$[Mal]^{-1}$	2	-4.7
3	$[Mal]^{-1}$	3	-4.7	3	$[Mal]^{-1}$	3	-4.7
4	$[Mal]^{-1}$	4	-4.6	4	$[Mal]^{-1}$	4	-4.5
5	$[Mal]^{-1}$	5	-4.5	5	$[Mal]^{-1}$	5	-4.2
6	$[Mal]^{-1}$	6	-4.4	6	$[Mal]^{-1}$	6	-4.2
7	$[Mal]^{-1}$	7	-4.4	7	$[Mal]^{-1}$	7	-4.1
8	$[Mal]^{-1}$	8	-4.4	8	$[Mal]^{-1}$	8	-4.1
9	$[Mal]^{-1}$	9	-4.4	9	$[Mal]^{-1}$	9	-4.1
10	$[Mal]^{-1}$	10	-4.3	10	$[Mal]^{-1}$	10	-4.0

Table S7. Docked scores of $[Mal]^{-2}$ for GOx and HRP.

GOx				HRP			
Rank	Name	Pose	Score	Rank	Name	Pose	Score
1	$[Mal]^{-2}$	1	-4.6	1	$[Mal]^{-2}$	1	-4.5
2	$[Mal]^{-2}$	2	-4.5	2	$[Mal]^{-2}$	2	-4.5
3	$[Mal]^{-2}$	3	-4.5	3	$[Mal]^{-2}$	3	-4.4
4	$[Mal]^{-2}$	4	-4.5	4	$[Mal]^{-2}$	4	-4.4
5	$[Mal]^{-2}$	5	-4.5	5	$[Mal]^{-2}$	5	-4.4
6	$[Mal]^{-2}$	6	-4.4	6	$[Mal]^{-2}$	6	-4.3
7	$[Mal]^{-2}$	7	-4.3	7	$[Mal]^{-2}$	7	-4.2
8	$[Mal]^{-2}$	8	-4.3	8	$[Mal]^{-2}$	8	-4.0
9	$[Mal]^{-2}$	9	-4.0	9	$[Mal]^{-2}$	9	-4.0
10	$[Mal]^{-2}$	10	-4.0	10	$[Mal]^{-2}$	10	-4.0

Table S8. Docked scores of $[PAA]^{-1}$ for (a) GOx and (b) HRP.

GOx				HRP			
Rank	Name	Pose	Score	Rank	Name	Pose	Score
1	$[PAA]^{-1}$	1	-5.1	1	$[PAA]^{-1}$	1	-4.9
2	$[PAA]^{-1}$	2	-5.1	2	$[PAA]^{-1}$	2	-4.7
3	$[PAA]^{-1}$	3	-5,0	3	$[PAA]^{-1}$	3	-4.7
4	$[PAA]^{-1}$	4	-4.8	4	$[PAA]^{-1}$	4	-4.4
5	$[PAA]^{-1}$	5	-4.8	5	$[PAA]^{-1}$	5	-4.4
6	$[PAA]^{-1}$	6	-4.7	6	$[PAA]^{-1}$	6	-4.3
7	$[PAA]^{-1}$	7	-4.7	7	$[PAA]^{-1}$	7	-4.3
8	$[PAA]^{-1}$	8	-4.7	8	$[PAA]^{-1}$	8	-4.3
9	$[PAA]^{-1}$	9	-4.6	9	$[PAA]^{-1}$	9	-4.2
10	$[PAA]^{-1}$	10	-4.6	10	$[PAA]^{-1}$	10	-4.2

Table S9. Docked scores of $[PAA]^{-2}$ for GOx and HRP.

GOx				HRP			
Rank	Name	Pose	Score	Rank	Name	Pose	Score
1	$[PAA]^{-2}$	1	-5.0	1	$[PAA]^{-2}$	1	-5.1
2	$[PAA]^{-2}$	2	-4.9	2	$[PAA]^{-2}$	2	-5.0
3	$[PAA]^{-2}$	3	-4.9	3	$[PAA]^{-2}$	3	-4.9
4	$[PAA]^{-2}$	4	-4.9	4	$[PAA]^{-2}$	4	-4.7
5	$[PAA]^{-2}$	5	-4.9	5	$[PAA]^{-2}$	5	-4.4
6	$[PAA]^{-2}$	6	-4.8	6	$[PAA]^{-2}$	6	-4.2
7	$[PAA]^{-2}$	7	-4.8	7	$[PAA]^{-2}$	7	-4.2
8	$[PAA]^{-2}$	8	-4.6	8	$[PAA]^{-2}$	8	-4.2
9	$[PAA]^{-2}$	9	-4.5	9	$[PAA]^{-2}$	9	-4.1
10	$[PAA]^{-2}$	10	-4.5	10	$[PAA]^{-2}$	10	-4.1

Table S10. Docked scores of $[PAA]^{-3}$ for GOx and HRP.

GOx				HRP			
Rank	Name	Pose	Score	Rank	Name	Pose	Score
1	$[PAA]^{-3}$	1	-4.9	1	$[PAA]^{-3}$	1	-4.8
2	$[PAA]^{-3}$	2	-4.9	2	$[PAA]^{-3}$	2	-4.7
3	$[PAA]^{-3}$	3	-4.8	3	$[PAA]^{-3}$	3	-4.6
4	$[PAA]^{-3}$	4	-4.8	4	$[PAA]^{-3}$	4	-4.5
5	$[PAA]^{-3}$	5	-4.7	5	$[PAA]^{-3}$	5	-4.3
6	$[PAA]^{-3}$	6	-4.7	6	$[PAA]^{-3}$	6	-4.3
7	$[PAA]^{-3}$	7	-4.6	7	$[PAA]^{-3}$	7	-4.3
8	$[PAA]^{-3}$	8	-4.5	8	$[PAA]^{-3}$	8	-4.1
9	$[PAA]^{-3}$	9	-4.5	9	$[PAA]^{-3}$	9	-4.1
10	$[PAA]^{-3}$	10	-4.5	10	$[PAA]^{-3}$	10	-3.8

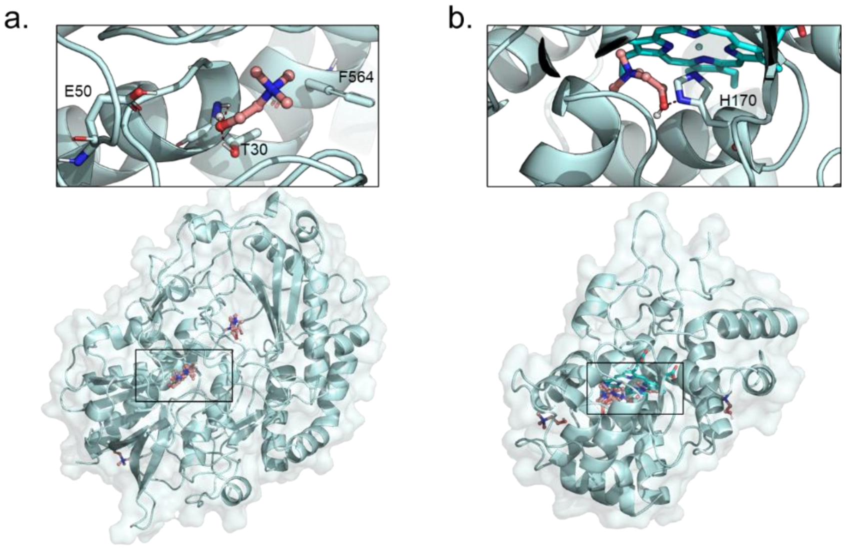

Figure S12. (a, b) All docked configurations of $[Ch]^{+1}$ at different putative binding sites of GOx and HRP respectively. The zoomed in configuration represents the docked pose with highest binding affinity and interacting residues.

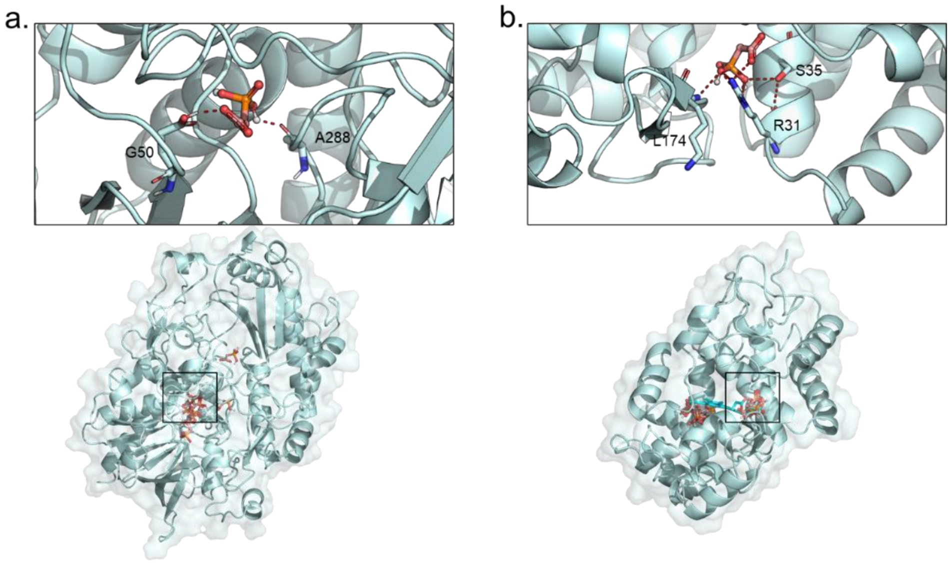

Figure S13. (a, b) All docked configurations of $[PAA]^{-1}$ at different putative binding sites of GOx and HRP respectively. The zoomed in configuration represents the docked pose with highest binding affinity and interacting residues.

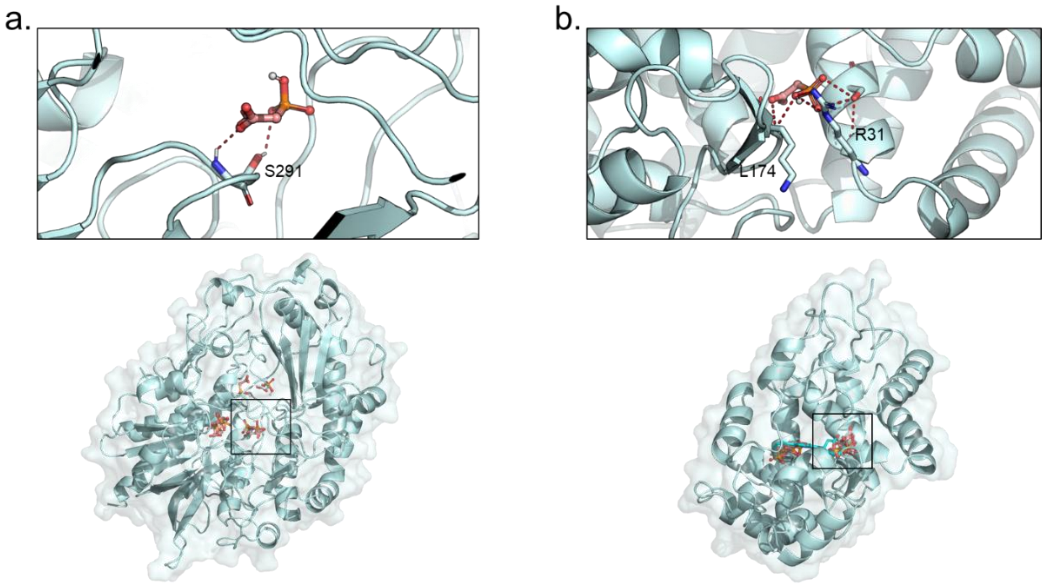

Figure S14. (a, b) All docked configurations of $[PAA]^{-2}$ at different putative binding sites of GOx and HRP respectively. The zoomed in configuration represents the docked pose with highest binding affinity and interacting residues.

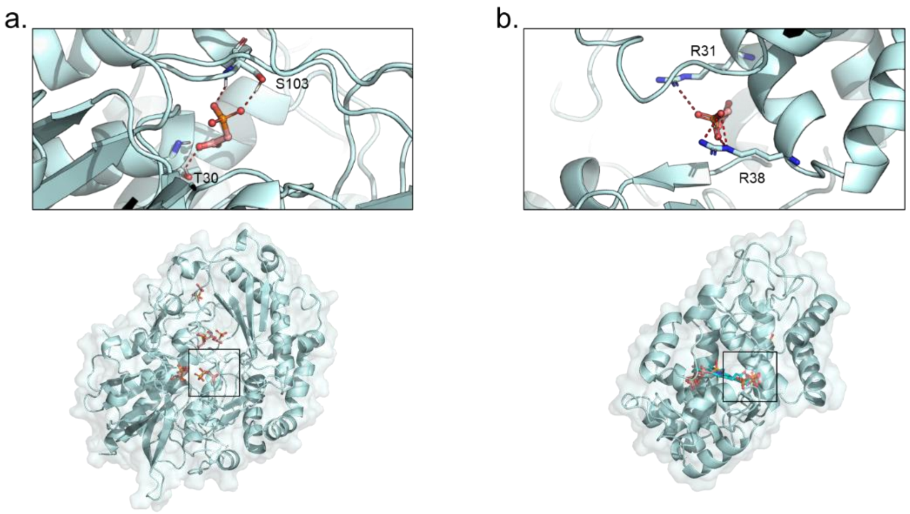

Figure S15. (a, b) All docked configurations of $[PAA]^{-3}$ at different putative binding sites of GOx and HRP respectively. The zoomed in configuration represents the docked pose with highest binding affinity and interacting residues.

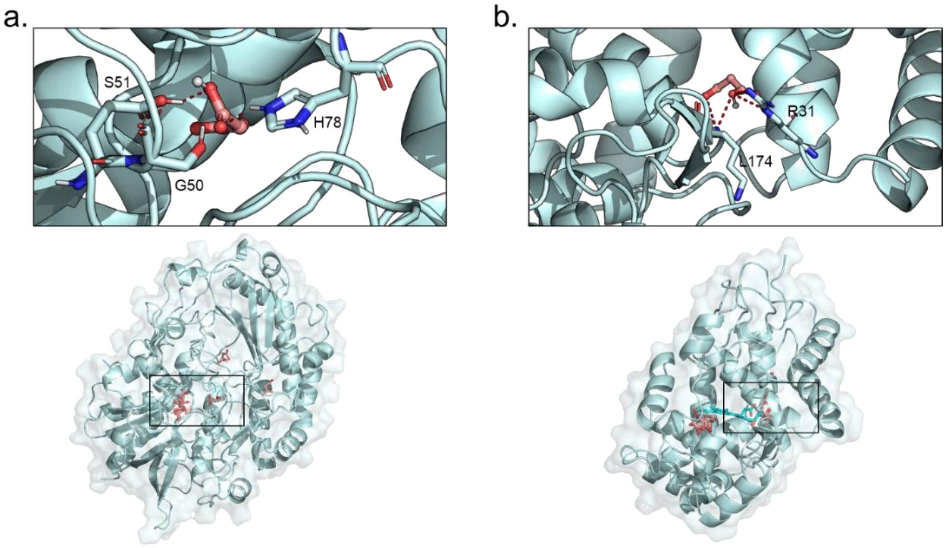

Figure S16. (a, b) All docked configurations of $[Mal]^{-1}$ at different putative binding sites of GOx and HRP respectively. The zoomed in configuration represents the docked pose with highest binding affinity and interacting residues.

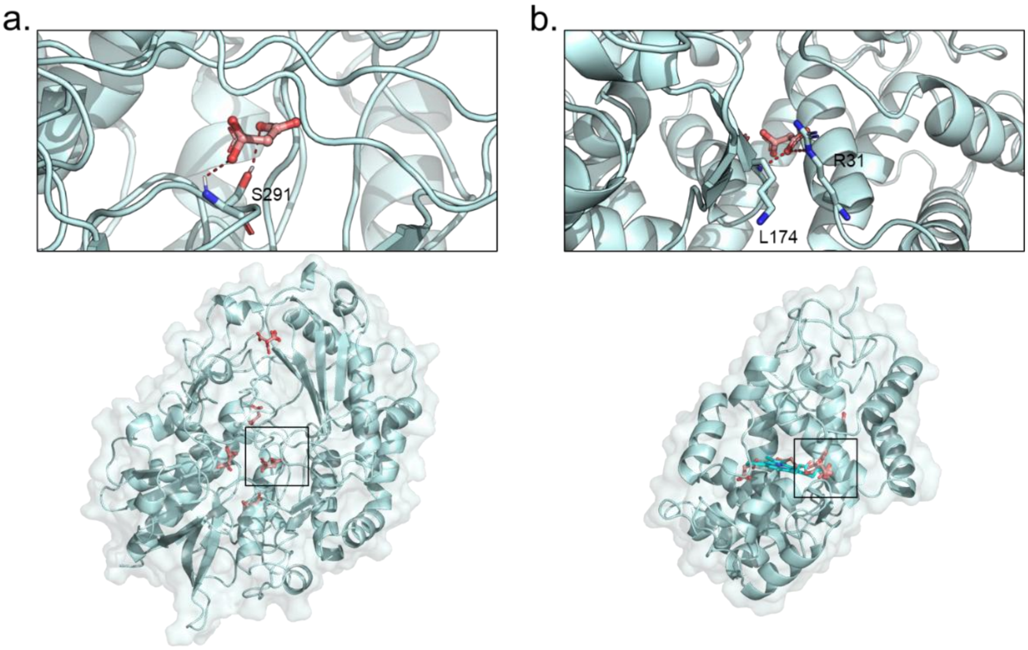

Figure S17. (a, b) All docked configurations of $[Mal]^{-2}$ at different putative binding sites of GOx and HRP respectively. The zoomed in configuration represents the docked pose with highest binding affinity and interacting residues.

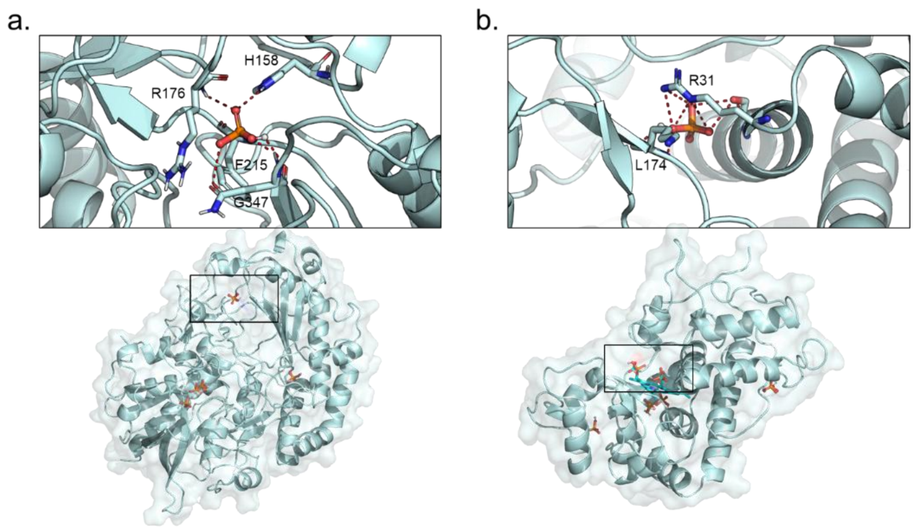

Figure S18. (a, b) All docked configurations of $[Dhp]^{-1}$ at different putative binding sites of GOx and HRP respectively. The zoomed in configuration represents the docked pose with highest binding affinity and interacting residues.

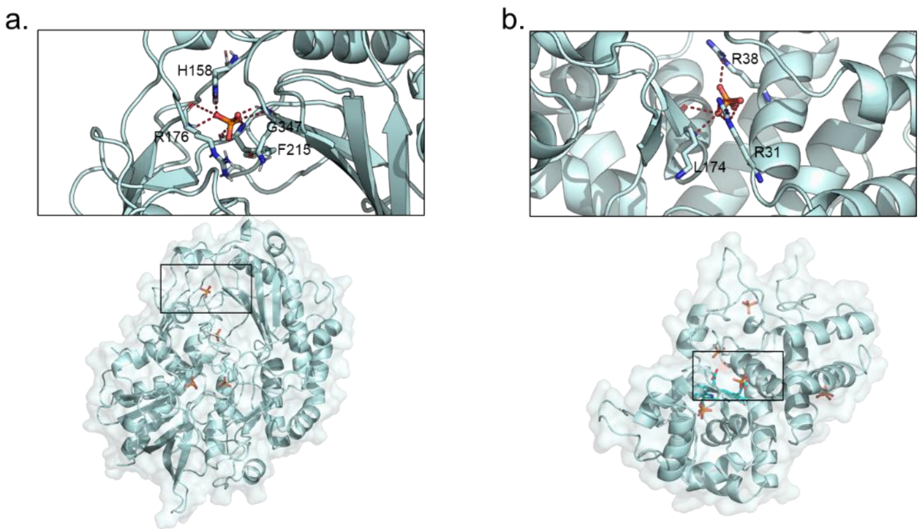

Figure S19. (a, b) All docked configurations of $[Dhp]^{-2}$at different putative binding sites of GOx and HRP respectively. The zoomed in configuration represents the docked pose with highest binding affinity and interacting residues.

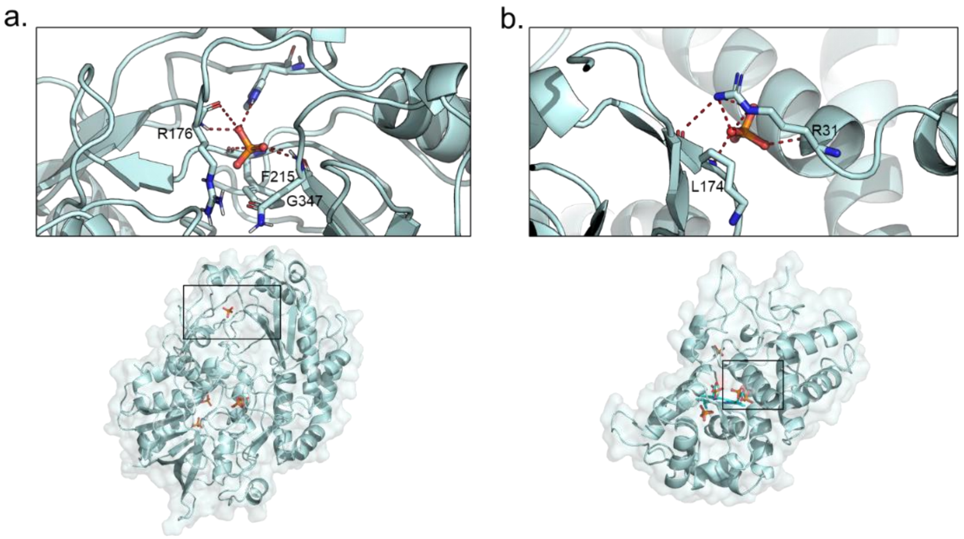

Figure S20. (a, b) All docked configurations of $[Dhp]^{-3}$ at different putative binding sites of GOx and HRP respectively. The zoomed in configuration represents the docked pose with highest binding affinity and interacting residues.

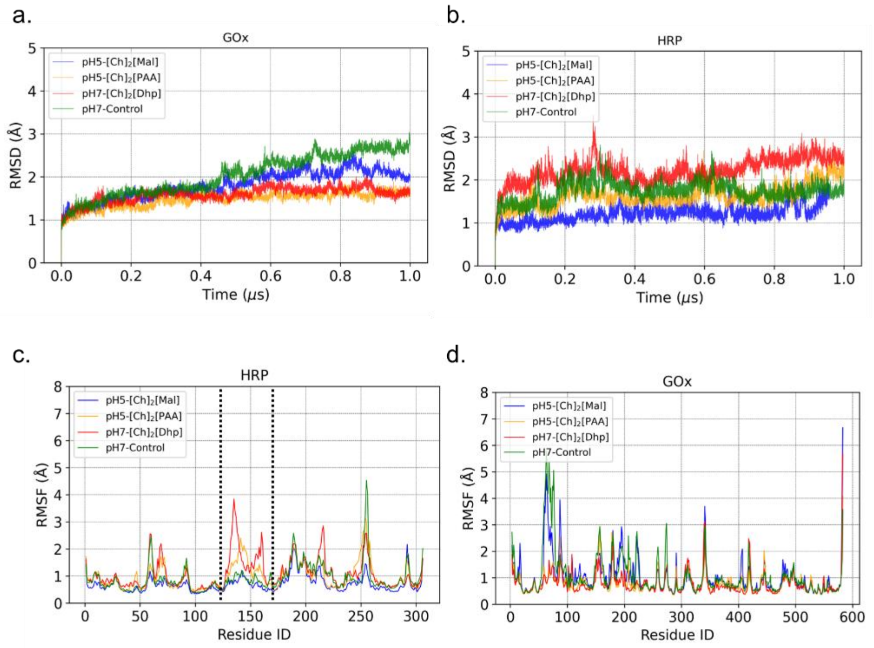

Figure S21. Dynamics of GOx and HRP in presence of 2:1 IL composition. (a-b) RMSD of GOx and HRP from 1 µs of unbiased MD simulations obtained from four different conditions: pH 5 with $[Ch]_2[Mal]$ (blue), pH 5 with $[Ch]_2[PAA]$ (orange), pH 7 with $[Ch]_2[Dhp]$ (red), and pH 7 control without IL (green). (c-d) RMSF plot of HRP and GOx under four simulation conditions: pH 5 with $[Ch]_2[Mal]$ (blue), pH 5 with $[Ch]_2[PAA]$ (orange), pH 7 with $[Ch]_2[Dhp]$ (red), and pH 7 control without IL (green). The region near residues 130-180 are highlighted by a vertical dashed lines for HRP, these regions correspond to the highly dynamics regions (c).

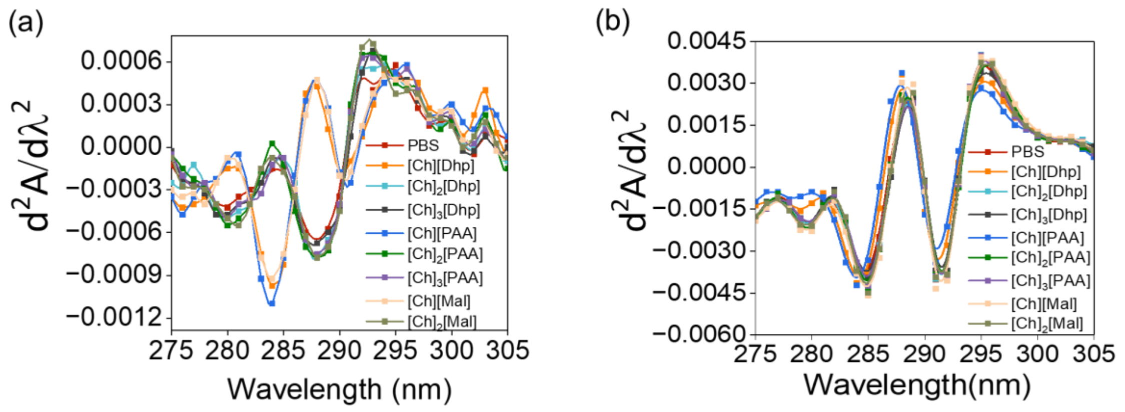

Figure S22: Analysis of UV-Vis absorption spectra of HRP and GOx in all ILs (10 wt%) including PBS (pH 7.4). Enzyme concentration for both HRP and GOx was fixed to 0.125 mg/mL. (a) Shows the second derivative $d^2A/d\lambda^2$ UV spectra of HRP in the range 275-305 nm in all IL media. (b) Represents the second derivative $d^2A/d\lambda^2$ UV spectra of GOx in the range 275-305 nm in all IL.

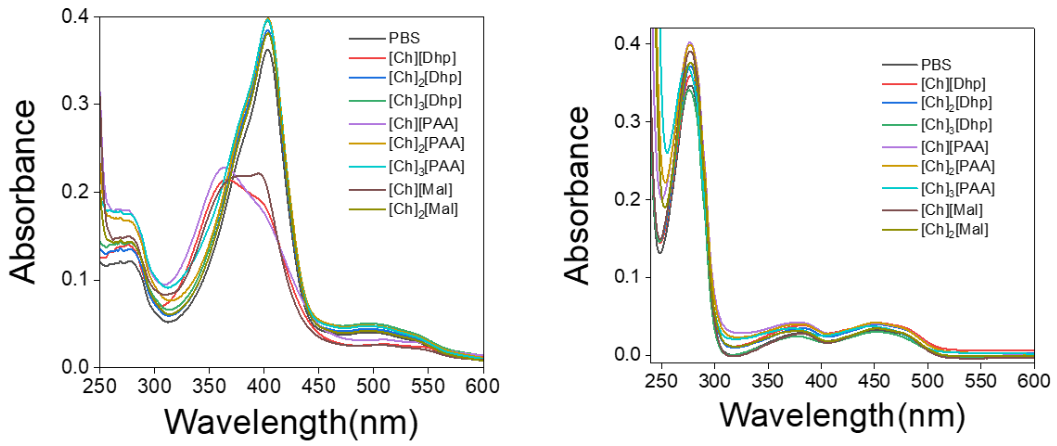

Figure S23: UV-Vis spectra of HRP (left side) and GOx (right side) in different ILs with 10 wt% ILs and control PBS (pH 7.4). The concentration of the enzyme was fixed to 0.125 mg/mL. Prior to the experiment all the enzyme containing solution was incubated for 5 minutes at 37 ^{o}C.

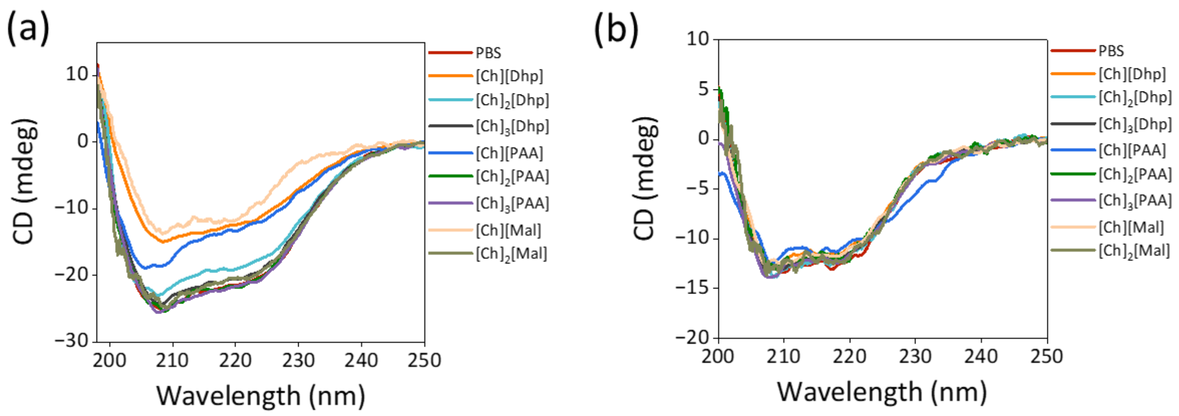

Figure S24: Analysis of CD spectra of HRP and GOx in all synthesized ILs (1 wt%) including PBS (pH 7.4). (a) shows CD spectra of HRP in all the ILs media, (b) depicts CD spectra of GOx in all the ILs. Enzyme concentration for both HRP and GOx was fixed to 0.125 mg/mL.

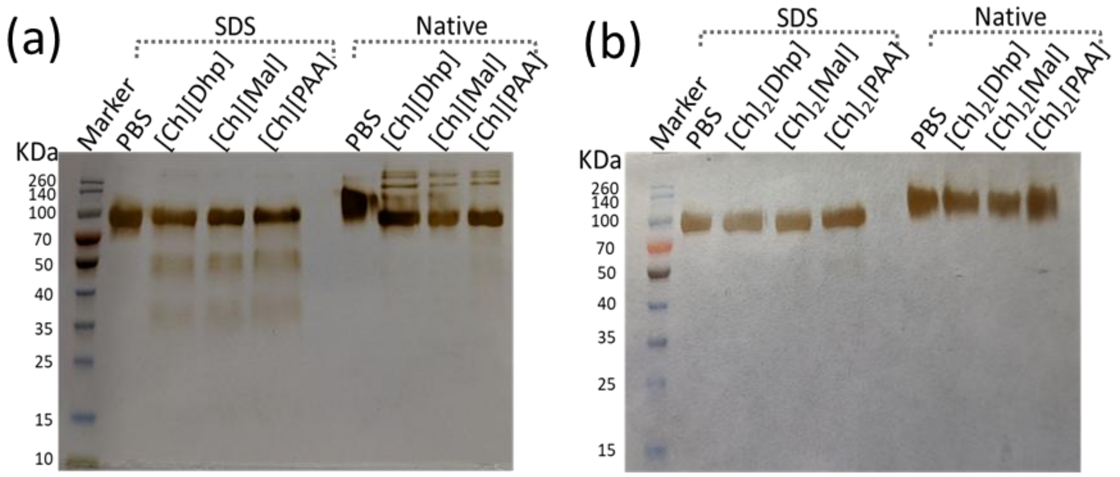

Figure S25: (a) Represents reduced and native PAGE image of GOx in (1:1) ILs. (b) Pictorial representation of reduced and native PAGE of GOx in (2:1) ILs showing stability in [Ch]2[Dhp], [Ch]2[Mal], and [Ch]2[PAA]. For each sample 5 µg in 30 µL of enzyme was used.

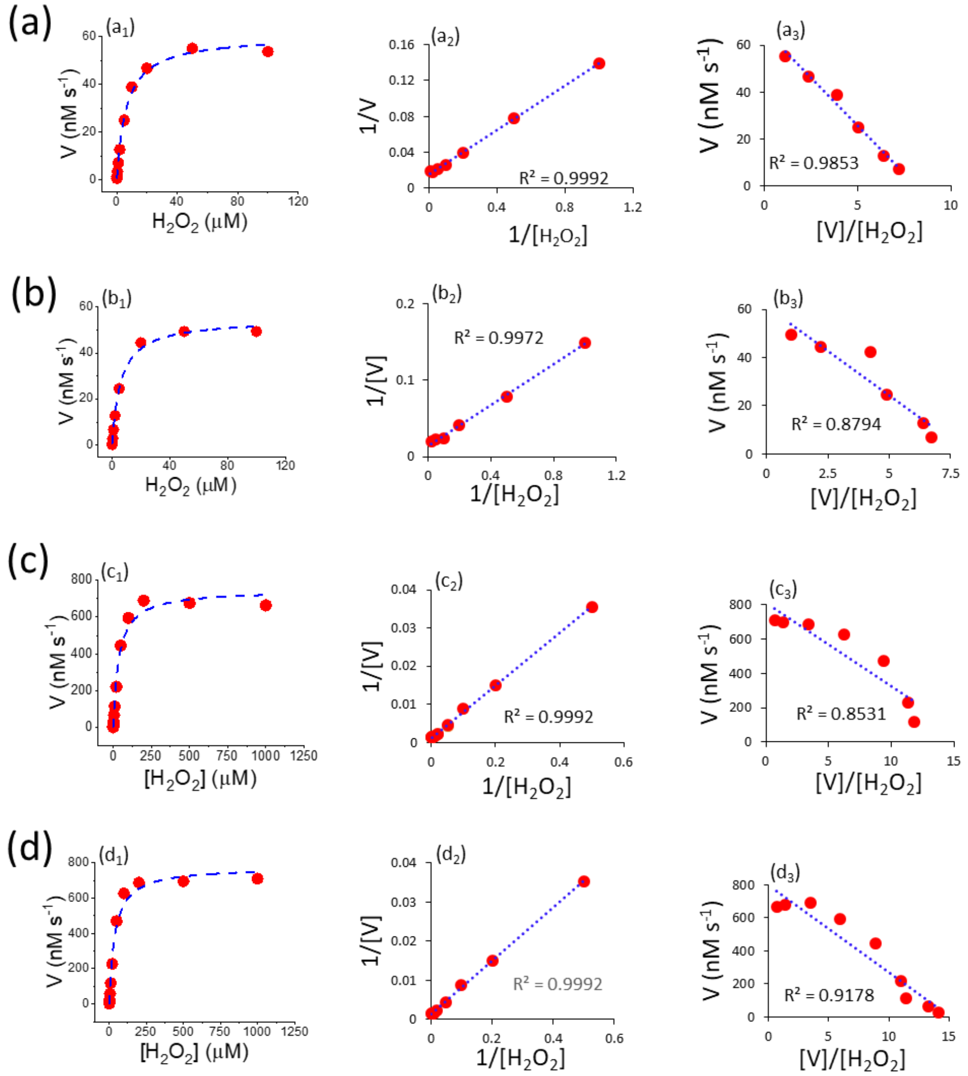

Figure S26: Kinetic parameters for HRP catalyzed reaction. Figure (a)-(d) shows all enzymatic kinetic model of PBS (pH 7.4), [Ch][Dhp], [Ch][Mal], and [Ch][PAA] respectively and suffix 1-3 represents validation of the obtained data using Michaelis-Menten (M-M), Lineweaver-Burk (L-B), and Eadie-Hofstee (E-H) model. The ILs concentration was fixed at 10 wt% for all the cases, the H_2O_2 concentration was varied from 0-1000 µM, reaction time was fixed to 3 minutes and incubation time was 5 minutes at 37 °C for all the cases.

The fitted data was then used to calculate the kinetic parameters such as maximum velocity for the enzymatic reaction (V_{max}), Michaelis-Menten constant (K_m), the catalytic rate constant (K_{cat}) and finally these obtained values were used to evaluate the catalytic efficiency (K_{cat}/K_m) for all the systems. All the obtained parameters are tabulated in Table S11-S14.

Table S11: Kinetic parameters for HRP in PBS

Kinetic parameters	PBS 7.4		
	M-M	L-B	E-H
V_{max} (nM s^{-1})	60.22±1.56	64.51±2.79	65.98±2.38
K_m (mM)	0.0064±0.0006	0.008±0.00059	0.0081±0.00049
K_{cat} (s^{-1})	60.22±1.56	64.51±2.79	65.98±2.38
K_{cat}/K_m (mM s^{-1})	9350.93	8058.02	8125.62

Table S12: Kinetic parameters for HRP in $[Ch]_2$[PAA]

Kinetic parameters	$[Ch]_2$[PAA] (2:1) 10%		
	M-M	L-B	E-H
V_{max} (nM s^{-1})	778.87±27.49	833.33±28.27	811.91±68.78
K_m (mM)	0.0387±0.0054	0.0517±0.006	0.0484±0.0089
K_{cat} (s^{-1})	778.87±27.49	833.33±28.27	811.91±68.78
K_{cat}/K_m (mM s^{-1})	20125.84	16129.03	16781.93

Table S13: Kinetic parameters for HRP in $[Ch]_2$[Mal]

Kinetic parameters	$[Ch]_2$[Mal] (2:1) 10%		
	M-M	L-B	E-H
V_{max} (nM s^{-1})	737.18±27.08	769.23±83.1	799.21±54
K_m (mM)	0.0374±0.0055	0.052769±0.0062	0.05272±0.0059
K_{cat} (s^{-1})	737.18±27.08	769.23±83.1	799.21±54
K_{cat}/K_m (mM s^{-1})	19689.64	14577.26	15159.52

Table S14: Kinetic parameters for HRP in $[Ch]_2$[Dhp]

Kinetic parameters	$[Ch]_2$[DhP] (2:1) 10%		
	M-M	L-B	E-H
V_{max} (nM s^{-1})	54.62±1.37	69.93±6.57	57.46±4.85
K_m (mM)	0.0061±0.0006	0.00932±0.0009	0.0073±0.001
K_{cat} (s^{-1})	54.62±1.37	69.93±6.57	57.46±4.85
K_{cat}/K_m (mM s^{-1})	8954.1	7503.22	8404.66

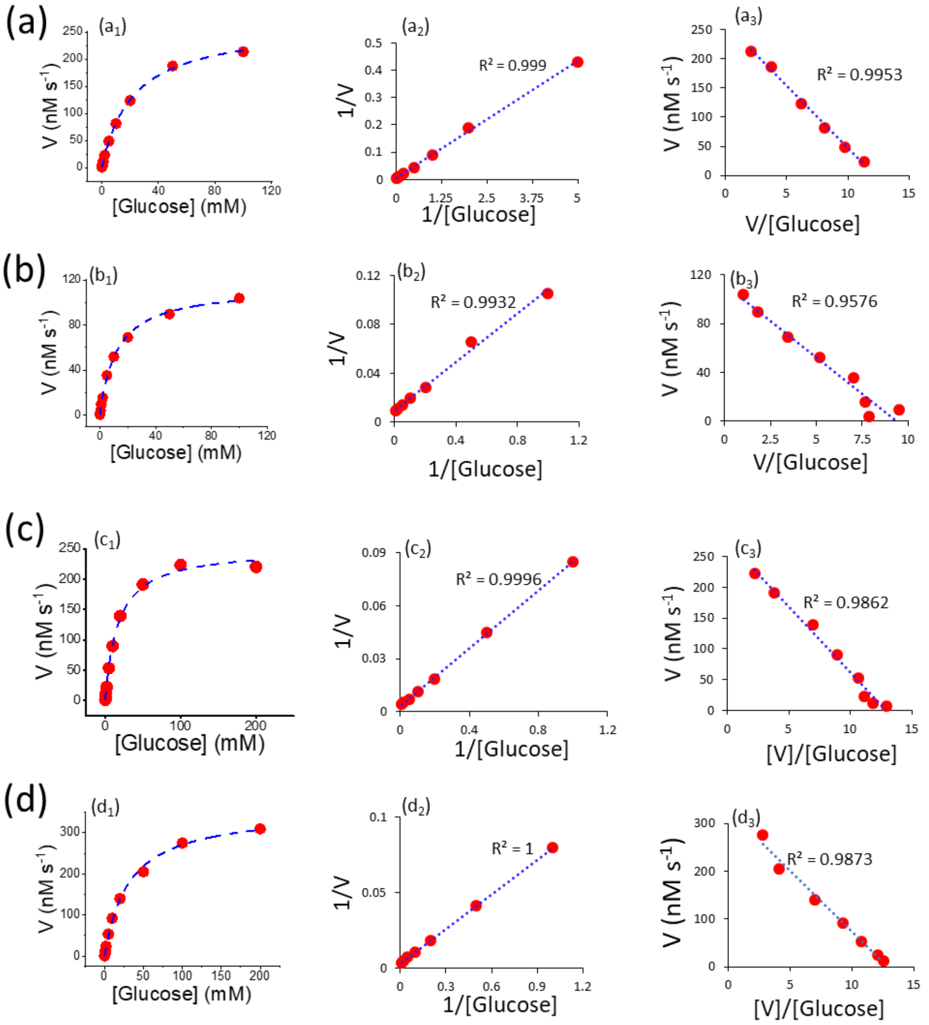

Figure S27: Kinetic parameters for GOx catalyzed reaction. Figure (a)-(d) shows all enzymatic kinetic model of PBS (pH 7.4), [Ch][Dhp], [Ch][Mal], and [Ch][PAA] respectively and suffix 1-3 represents validation of the obtained data using Michaelis-menten (M-M), Linweaver-Burk (L-B), Eadie-Hofstee (E-H) model. The ILs concentration was fixed at 10 wt% for all the cases, the D-glucose concentration was varied from 0-200 mM, reaction time was fixed to 3 minutes and incubation time was 5 minutes at 37 °C for all the cases.

The fitted data was then used to calculate the kinetic parameters such as maximum velocity for the enzymatic reaction (V_{max}), Michaelis-Menten constant (K_m), the catalytic rate constant (K_{cat}) and finally these obtained values were used to evaluate the catalytic efficiency (K_{cat}/K_m) for all the systems. All the obtained parameters are tabulated in Table S15-S18.

Table S15: Kinetic parameters for GOx in PBS

Kinetic parameters	PBS 7.4		
	M-M	L-B	E-H
V_{max} (nM s^{-1})	220±3.26	243.9±12.96	260.74±5.62
K_m (mM)	17.89±0.74	21.04±0.78	21.49±0.73
K_{cat} (s^{-1})	220±3.26	243.9±12.96	260.74±5.62
K_{cat}/K_m (mM s^{-1})	12.29	11.58	12.13

Table S16: Kinetic parameters for GOx in $[Ch]_2[PAA]$

Kinetic parameters	$[Ch]_2[PAA]$ (2:1) 10%		
	M-M	L-B	E-H
V_{max} (nM s^{-1})	286.12±7.76	294.11±18.36	316.28±6.43
K_m (mM)	19.86±1.98	21.64±1.02	24.32±0.75
K_{cat} (s^{-1})	286.12±7.76	294.11±19.56	316.28±6.43
K_{cat}/K_m (mM s^{-1})	14.41	13.58	13.01

Table S17: Kinetic parameters for GOx in $[Ch]_2[Mal]$

Kinetic parameters	$[Ch]_2[Mal]$ (2:1) 10%		
	M-M	L-B	E-H
V_{max} (nM s^{-1})	237.02±5.47	200±35.29	255.6±8.21
K_m (mM)	18.19±1.29	15.16±0.99	19.99±0.96
K_{cat} (s^{-1})	237.02±5.47	200±35.29	255.6±8.21
K_{cat}/K_m (mM s^{-1})	13.03	13.19	12.78

Table S18: Kinetic parameters for GOx in $[Ch]_2[Dhp]$

Kinetic parameters	$[Ch]_2[DhP]$ (2:1) 10%		
	M-M	L-B	E-H
V_{max} (nM s^{-1})	100±1.8	102.04±16.71	112.35±6.3
K_m (mM)	10±0.6	10.05±0.97	11.97±1.02
K_{cat} (s^{-1})	100±1.8	102.04±16.71	112.35±6.3
K_{cat}/K_m (mM s^{-1})	10.00	10.15	9.38

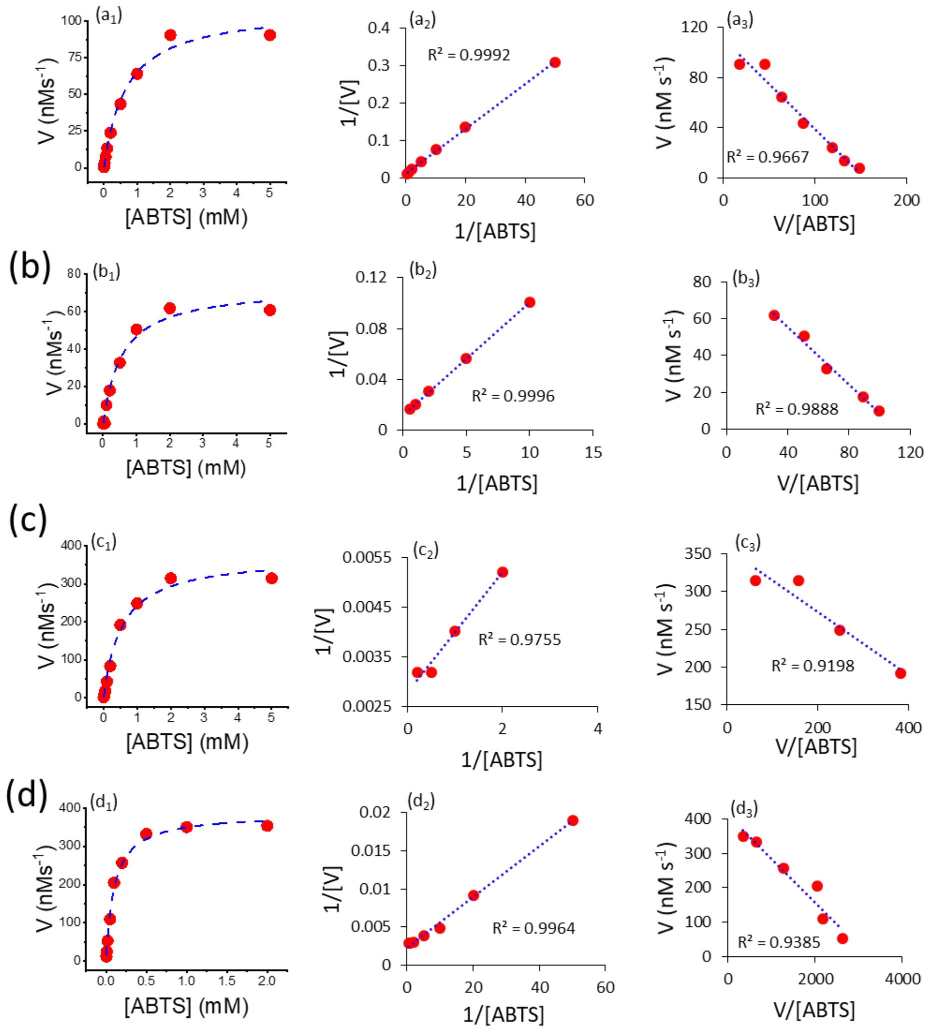

Figure S28: The figure represents the kinetic parameters obtained upon analysis of GOx-HRP Cascade reaction. Figure (a)-(d) shows all enzymatic kinetic model of PBS (pH 7.4), [Ch][Dhp], [Ch][Mal], and [Ch][PAA] respectively and suffix 1-3 represents validation of the obtained data using Michaelis-Menten (M-M), Lineweaver-Burk (L-B), Eadie-Hofstee (E-H) model. The ILs concentration was fixed at 10 wt% for all the cases, the ABTS concentration was varied till the saturation limit has achieved (0-5 mM), reaction time was fixed to 3 minutes and incubation time was 5 minutes at 37 °C for all the cases.

The fitted data was then used to calculate the kinetic parameters such as maximum velocity for the enzymatic reaction (V_{max}), Michaelis-Menten constant (K_m), the catalytic rate constant (K_{cat}) and finally these obtained values were used to evaluate the catalytic efficiency (K_{cat}/K_m) for all the systems. All the obtained parameters are tabulated in Table S19-S22.

Table S19: Kinetic parameters for GOx-HRP cascade system in PBS

Kinetic parameters	PBS 7.4		
	M-M	L-B	E-H
V_{max} (nM s^{-1})	102.95±5.19	86.2±10.01	110.43±5.8
K_m (mM)	0.65±0.09	0.51±0.079	0.7167±0.06
K_{cat} (s^{-1})	102.95±5.19	86.2±10.01	110.43±5.8
K_{cat}/K_m (mM s^{-1})	158.38	166.67	154.08

Table S20: Kinetic parameters for GOx-HRP cascade system in $[Ch]_2[PAA]$

Kinetic parameters	$[Ch]_2[PAA]$ (2:1) 10%		
	M-M	L-B	E-H
V_{max} (nM s^{-1})	386.31±10.02	434.78±22.42	412.96±28
K_m (mM)	0.104±0.01	0.13±0.0097	0.13±0.01
K_{cat} (s^{-1})	386.31±10.02	434.78±22.42	412.96±28
K_{cat}/K_m (mM s^{-1})	3714.51	3333.33	3231.29

Table S21: Kinetic parameters for GOx-HRP cascade system in $[Ch]_2[Mal]$

Kinetic parameters	$[Ch]_2[Mal]$ (2:1) 10%		
	M-M	L-B	E-H
V_{max} (nM s^{-1})	372.77±18.28	357.14±28.45	355.94±21.1
K_m (mM)	0.53±0.08	0.42±0.0779	0.41±0.08
K_{cat} (s^{-1})	372.77±18.28	357.14±28.45	355.94±21.8
K_{cat}/K_m (mM s^{-1})	691.59	833.33	857.89

Table S22: Kinetic parameters for GOx-HRP cascade system in $[Ch]_2[Dhp]$

Kinetic parameters	$[Ch]_2[DhP]$ (2:1) 10%		
	M-M	L-B	E-H
V_{max} (nM s^{-1})	73.25±4.3	84.74±5.29	86.3±3.39
K_m (mM)	0.55±0.1	0.75±0.068	0.77±0.04
K_{cat} (s^{-1})	73.25±4.3	84.74±5.29	86.3±3.39
K_{cat}/K_m (mM s^{-1})	133.18	112.35	111.8

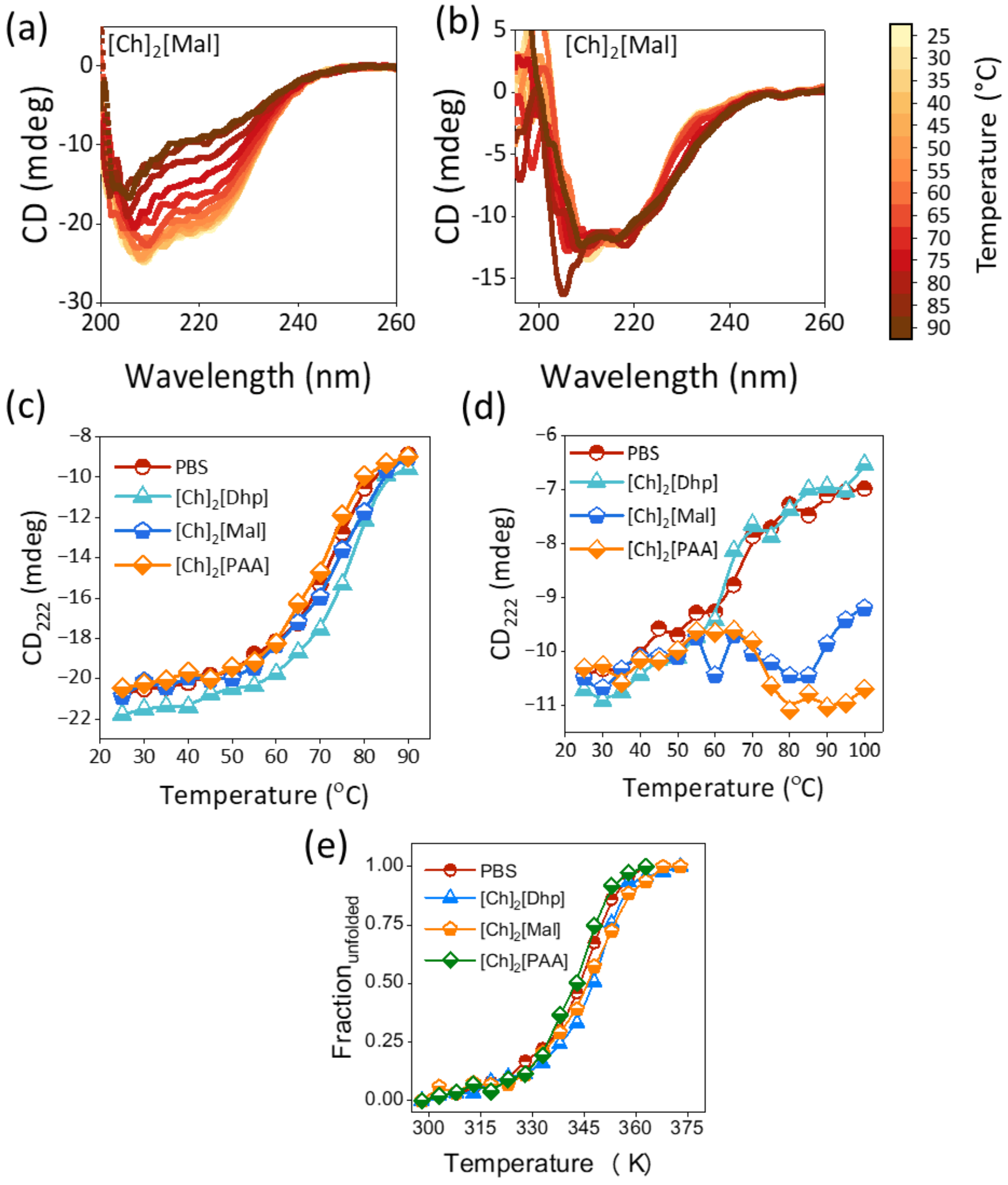

Figure S29: (a-b) Represent thermal denaturation CD spectra within the temperature range 25-90 °C of HRP and GOx, respectively, in $[Ch]_2[Mal]$. (c-d) Temperature vs change in CD value (mdeg) at 222 nm for HRP and GOx, respectively. (e) Fraction unfolded v/s temperature plot for HRP in different ILs.

Table S23: Literature comparison of GOx-HRP cascade in respect to kinetic efficiency and thermostability.

Entry	Scaffold name	K_{cat}/K_m (Fold enhanced compared to PBS, pH 7.4)	Ref
1	Scaffold-free dynamic modulation in 10 wt% $[Ch]_2[PAA]$ IL	**25**	**Present work**
2	DNA origami rectangle	10	23
3	Supramolecular cocaine-aptamer complex	13	24
4	dsDNA with azobenzene moieties	25	25
5	DNA strands synthesized using Rolling circle amplification (RCA)	18	26
6	Hexagon-like DNA strips	15.8	27
7	Rectangular and tubular DNA origami	8	28
8	Nanocage DNA origami	8	29
9	Metal–organic frameworks (ZIF8-NMOFs)	7.5	30
10	Polymerization-induced co-assembly (PICA) of poly(2-hydroxypropyl methacrylate) (PHPMA)	4.9	31
11	Suprastructures of SiO_2 or TiO_2 nanoparticle	2.9	32
12	Wireframe DNA origami scaffolds	9.1	33
13	Metal-Hollow Inorganic Spheres (M-HISs)	11.72	34
14	Super DNA-enzymes molecule (SDEM)	1.6	35
16	Tetrahedral DNA framework	5.9	36
17	Multi-shelled ZIF-8 MOFs	13.5	37
18	Smart polymer-UiO-66-NH_2	2.01-fold k_{cat}	38

Table S24: Melting temperature (T_m) and half-life temperature (T_{50}) of different enzymes in 10 wt% ILs solution.

	HRP-T_m (°C)	HRP-T_{50} (°C)	GOx-T_m (°C)	Cascade-T_{50} (°C)
PBS	74.79 ± 2.115	63.62 ± 0.346	62.85 ± 3.1	52.05 ± 0.07
$[Ch]_2$[Dhp]	78.08 ± 2.555	64.2 ± 1.146	66.38 ± 2.25	51.45 ± 0.25
$[Ch]_2$[Mal]	74.8 ± 1.705	64.09 ± 0.66	66.38 ± 2.25	53.07 ± 0.06
$[Ch]_2$[PAA]	74.8 ± 1.705	64.25 ± 0.208	73.31 ± 3.97	60.53 ± 0.29

Table S25: ΔG values of HRP and GOx and various temperature in 10 wt% ILs solution.

Temperature (K)	PBS ΔG ($KJmol^{-1}$)		$[Ch]_2$[Dhp] ΔG ($KJmol^{-1}$)		$[Ch]_2$[Mal] ΔG ($KJmol^{-1}$)		$[Ch]_2$[PAA] ΔG ($KJmol^{-1}$)	
	HRP	GOx	HRP	GOx	HRP	GOx	HRP	GOx
328	4.36	1.90	5.63	2.68	5.61	-1.43	5.59	-12.25
333	3.48	1.84	4.60	1.82	3.78	--	3.95	-9.55
338	2.40	0.18	3.21	-1.56	2.50	-1.82	1.54	--
343	0.45	-3.04	1.99	-3.06	1.27	0.88	-0.03	-5.10
348	-2.10	-3.82	-0.08	-2.38	-0.86	2.34	-3.13	2.78
353	-5.33	-7.02	-3.36	-4.22	-2.82	5.13	-7.08	--
358	-9.29	-5.33	-7.82	-6.36	-6.05	5.15	-10.74	4.56

References

1. Mukesh, C., Mondal, D., Sharma, M., and Prasad, K. (2013). Rapid dissolution of DNA in a novel bio-based ionic liquid with long-term structural and chemical stability: successful recycling of the ionic liquid for reuse in the process. Chemical Communications *49*, 6849–6851.
2. Zhang, Y., Tsitkov, S., and Hess, H. (2016). Proximity does not contribute to activity enhancement in the glucose oxidase–horseradish peroxidase cascade. Nature Communications *7*, 13982.
3. Bharadwaj, P., Barua, A., Bisht, M., Sarkar, D.K., Biswas, S., Franklin, G., and Mondal, D. (2024). Understanding the Effect of Ionic Liquid–Mediated Solvent Engineering on the Kinetics and Thermodynamic Stability of Phenylalanine Ammonia-Lyase. The Journal of Physical Chemistry B *128*, 9102–9110.
4. Laemmli, U.K. (1970). Cleavage of Structural Proteins during the Assembly of the Head of Bacteriophage T4. Nature *227*, 680–685.
5. Shmool, T.A., Martin, L.K., Matthews, R.P., and Hallett, J.P. (2022). Ionic liquid-based strategy for predicting protein aggregation propensity and thermodynamic stability. Jacs Au *2*, 2068–2080.
6. Eberhardt, J., Santos-Martins, D., Tillack, A.F., and Forli, S. (2021). AutoDock Vina 1.2. 0: new docking methods, expanded force field, and python bindings. Journal of chemical information and modeling *61*, 3891–3898.
7. Trott, O., and Olson, A.J. (2010). AutoDock Vina: Improving the speed and accuracy of docking with a new scoring function, efficient optimization, and multithreading. Journal of Computational Chemistry *31*, 455–461.
8. Schrödinger, L., and DeLano, W. (2020). PyMOL available at: http://www.pymol.org/pymol

9. Jo, S., Kim, T., Iyer, V.G., and Im, W. (2008). CHARMM-GUI: a web-based graphical user interface for CHARMM. Journal of computational chemistry *29*, 1859–1865.
10. Huang, L., and Roux, B. (2013). Automated force field parameterization for nonpolarizable and polarizable atomic models based on ab initio target data. Journal of chemical theory and computation *9*, 3543–3556.
11. Páll, S., and Hess, B. (2013). A flexible algorithm for calculating pair interactions on SIMD architectures. Computer Physics Communications *184*, 2641–2650.
12. Darden, T., York, D., and Pedersen, L. (1993). Particle mesh Ewald: An N log (N) method for Ewald sums in large systems. Journal of chemical physics *98*, 10089–10089.
13. Hess, B., Bekker, H., Berendsen, H.J., and Fraaije, J.G. (1997). LINCS: A linear constraint solver for molecular simulations. Journal of computational chemistry *18*, 1463–1472.
14. Miyamoto, S., and Kollman, P.A. (1992). Settle: An analytical version of the SHAKE and RATTLE algorithm for rigid water models. Journal of computational chemistry *13*, 952–962.
15. Schubert, E., Sander, J., Ester, M., Kriegel, H.P., and Xu, X. (2017). DBSCAN revisited, revisited: why and how you should (still) use DBSCAN. ACM Transactions on Database Systems (TODS) *42*, 1–21.
16. Pedregosa, F., Varoquaux, G., Gramfort, A., Michel, V., Thirion, B., Grisel, O., Blondel, M., Prettenhofer, P., Weiss, R., and Dubourg, V. (2011). Scikit-learn: Machine learning in Python. the Journal of machine Learning research *12*, 2825–2830.
17. Abramson, Josh, et al. "Accurate structure prediction of biomolecular interactions with AlphaFold 3." Nature 630.8016 (2024): 493-500. https://doi.org/10.1038/s41586-024-07487-w
18. Yan, Yumeng, et al. "The HDOCK server for integrated protein–protein docking." Nature protocols 15.5 (2020): 1829-1852. https://doi.org/10.1038/s41596-020-0312-x
19. Singh, Amar, et al. "GRAMM web server for protein docking." Computational drug discovery and design. New York, NY: Springer US, 2023. 101-112. https://doi.org/10.1007/978-1-0716-3441-7_5
20. Anandakrishnan, Ramu, Boris Aguilar, and Alexey V. Onufriev. "H++ 3.0: automating p K prediction and the preparation of biomolecular structures for atomistic molecular modeling and simulations." Nucleic acids research 40.W1 (2012): W537-W541. https://doi.org/10.1093/nar/gks375
21. https://ccsb.scripps.edu/mgltools/ (accessed 28th September 2025).
22. O'Boyle, Noel M., et al. "Open Babel: An open chemical toolbox." Journal of cheminformatics 3.1 (2011): 33. https://doi.org/10.1186/1758-2946-3-33
23. Vogele, K., List, J., Simmel, F.C., and Pirzer, T. (2018). Enhanced efficiency of an enzyme cascade on DNA-activated silica surfaces. Langmuir *34*, 14780–14786.
24. Freeman, R., Sharon, E., Tel-Vered, R., and Willner, I. (2009). Supramolecular cocaine–aptamer complexes activate biocatalytic cascades. Journal of the American Chemical Society *131*, 5028–5029.
25. You, M., Wang, R.-W., Zhang, X., Chen, Y., Wang, K., Peng, L., and Tan, W. (2011). Photon-regulated DNA-enzymatic nanostructures by molecular assembly. ACS nano *5*, 10090–10095.
26. Wilner, O.I., Shimron, S., Weizmann, Y., Wang, Z.-G., and Willner, I. (2009). Self-assembly of enzymes on DNA scaffolds: en route to biocatalytic cascades and the synthesis of metallic nanowires. Nano letters *9*, 2040–2043.

27. Wilner, O.I., Weizmann, Y., Gill, R., Lioubashevski, O., Freeman, R., and Willner, I. (2009). Enzyme cascades activated on topologically programmed DNA scaffolds. Nature nanotechnology *4*, 249–254.
28. Fu, Y., Zeng, D., Chao, J., Jin, Y., Zhang, Z., Liu, H., Li, D., Ma, H., Huang, Q., and Gothelf, K.V. (2013). Single-step rapid assembly of DNA origami nanostructures for addressable nanoscale bioreactors. Journal of the American Chemical Society *135*, 696–702.
29. Zhao, Z., Fu, J., Dhakal, S., Johnson-Buck, A., Liu, M., Zhang, T., Woodbury, N.W., Liu, Y., Walter, N.G., and Yan, H. (2016). Nanocaged enzymes with enhanced catalytic activity and increased stability against protease digestion. Nature communications *7*, 10619.
30. Chen, W.-H., Vázquez-González, M., Zoabi, A., Abu-Reziq, R., and Willner, I. (2018). Biocatalytic cascades driven by enzymes encapsulated in metal–organic framework nanoparticles. Nature Catalysis *1*, 689–695.
31. Chiang, C.-W., Liu, X., Sun, J., Guo, J., Tao, L., and Gao, W. (2019). Polymerization-induced coassembly of enzyme–polymer conjugates into comicelles with tunable and enhanced cascade activity. Nano Letters *20*, 1383–1387.
32. Jo, S.M., Kim, J., Lee, J.E., Wurm, F.R., Landfester, K., and Wooh, S. (2022). Multimodal Enzyme-Carrying Suprastructures for Rapid and Sensitive Biocatalytic Cascade Reactions. Advanced science *9*, 2104884.
33. Kahn, J.S., Xiong, Y., Huang, J., and Gang, O. (2022). Cascaded enzyme reactions over a three-dimensional, wireframe DNA origami scaffold. Jacs Au *2*, 357–366.
34. Han, S.Y., Kim, N., Yun, G., Lee, H., and Choi, I.S. (2023). Tandem-biocatalysis reactors constructed by topological evolution of CaCO3 particles into hollow metal hydroxide spheres. Nature Communications *14*, 6828.
35. Zhao, H., Xiu, X., Li, M., Dai, S., Gou, M., Tao, L., Zuo, X., Fan, C., Tian, Z., and Song, P. (2023). Programming Super DNA-Enzyme Molecules for On-Demand Enzyme Activity Modulation. Angewandte Chemie *135*, e202214450.
36. Cao, N., Guo, R., Song, P., Wang, S., Liu, G., Shi, J., Wang, L., Li, M., Zuo, X., Yang, X., et al. (2024). DNA Framework–Programmed Nanoscale Enzyme Assemblies. Nano Letters *24*, 4682–4690.
37. Man, T., Xu, C., Liu, X.-Y., Li, D., Tsung, C.-K., Pei, H., Wan, Y., and Li, L. (2022). Hierarchically encapsulating enzymes with multi-shelled metal-organic frameworks for tandem biocatalytic reactions. Nature Communications *13*, 305.
38. Yalong Cheng, Juan Qiao, Shidi Zhang, Li Qi (2025). Enhancing catalytic activity of smart polymer-UiO-66@GOx-HRP through temperature change for colorimetric detection of glucose. Microchemical Journal, 214, 113960.
